# Evaluation of clinical and genetic factors in the population pharmacokinetics of carbamazepine

**DOI:** 10.1111/bcp.14667

**Published:** 2020-12-14

**Authors:** Vincent L.M. Yip, Henry Pertinez, Xiaoli Meng, James L. Maggs, Daniel F. Carr, B. Kevin Park, Anthony G. Marson, Munir Pirmohamed

**Affiliations:** ^1^ MRC Centre for Drug Safety Science, Department of Molecular and Clinical Pharmacology The University of Liverpool Liverpool UK; ^2^ The Wolfson Centre for Personalised Medicine, Department of Molecular and Clinical Pharmacology The University of Liverpool UK; ^3^ Department of Molecular and Clinical Pharmacology The University of Liverpool Liverpool UK

**Keywords:** carbamazepine, population pharmacokinetics, single nucleotide polymorphisms

## Abstract

**Aims:**

Carbamazepine can cause hypersensitivity reactions in ~10% of patients. An immunogenic effect can be produced by the electrophilic 10,11‐epoxide metabolite but not by carbamazepine. Hypothetically, certain single nucleotide polymorphisms might increase the formation of immunogenic metabolites, leading ultimately to hypersensitivity reactions. This study explores the role of clinical and genetic factors in the pharmacokinetics (PK) of carbamazepine and 3 metabolites known to be chemically reactive or formed through reactive intermediates.

**Methods:**

A combination of rich and sparse PK samples were collected from healthy volunteers and epilepsy patients. All subjects were genotyped for 20 single nucleotide polymorphisms in 11 genes known to be involved in the metabolism or transport of carbamazepine and carbamazepine 10,11‐epoxide. Nonlinear mixed effects modelling was used to build a population‐PK model.

**Results:**

In total, 248 observations were collected from 80 subjects. A 1‐compartment PK model with first‐order absorption and elimination best described the parent carbamazepine data, with a total clearance of 1.96 L/h, central distribution volume of 164 L and absorption rate constant of 0.45 h^−1^. Total daily dose and coadministration of phenytoin were significant covariates for total clearance of carbamazepine. *EPHX1*‐416G/G genotype was a significant covariate for the clearance of carbamazepine 10,11‐epoxide.

**Conclusion:**

Our data indicate that carbamazepine clearance was affected by total dose and phenytoin coadministration, but not by genetic factors, while carbamazepine 10,11‐epoxide clearance was affected by a variant in the microsomal epoxide hydrolase gene. A much larger sample size would be required to fully evaluate the role of genetic variation in carbamazepine pharmacokinetics, and thereby predisposition to carbamazepine hypersensitivity.

**What is already known about the subject**

- Metabolism of carbamazepine leads to the generation of reactive metabolites, such as the 10,11‐epoxide and arene oxides, which have been implicated in the pathogenesis of carbamazepine hypersensitivity reactions such as toxic epidermal necrolysis.
- Genetic variants in enzymes of carbamazepine metabolism and drug transporter proteins have been shown to alter plasma drug and metabolite levels and dosage requirements in patients.
- Pharmacokinetic modelling can be used to investigate the role of clinical and genetic factors in the metabolism of carbamazepine and its metabolites.

**What this study adds**

- A population pharmacokinetic model with first‐order absorption was developed for carbamazepine and its metabolites carbamazepine 10,11‐epoxide, 2‐hydroxycarbamazepine and 3‐hydroxycarbamazepine.
- Genetic variation in microsomal epoxide hydrolase (EPHX1; rs2234922) was associated with reduced clearance of carbamazepine 10,11 epoxide in homozygous GG genotypes compared with GA and AA genotypes.
- Genetic variation in drug transporters (ABCB1, ABCC2), cytochrome P450 isoforms (CYP2B6, CYP2C8, CYP2C19, CYP3A4) and other enzymes of drug metabolism (uridine diphosphate glucuronosyltransferase, myeloperoxidase, P450 oxidoreductase) had no effect on clearance of carbamazepine and its metabolites.

## 1 INTRODUCTION

**FIGURE 1:**
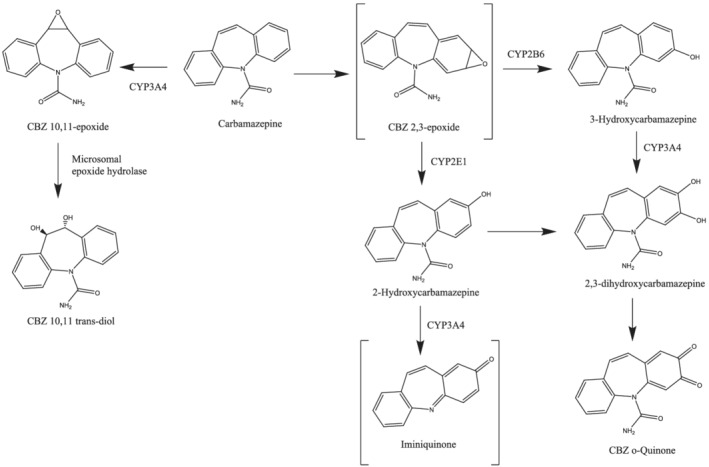
Proposed pathways for the oxidative bioactivation of carbamazepine in humans. The P450 isoforms shown are those reported to be the most active catalysts for the biotransformations. The depiction of the 2,3‐arene oxide as the sole product of aromatic epoxidation is purely representational; the number of arene oxides formed is unknown. The metabolic hydrolysis of carbamazepine 10,11‐epoxide is catalysed by microsomal epoxide hydrolase

Carbamazepine (CBZ) is used for the treatment of epilepsy, bipolar disorder and trigeminal neuralgia.^1^ Therapy with CBZ is complicated because of the drug's complex pharmacokinetic (PK) profile.^2^, ^3^, ^4^ CBZ is almost completely metabolised in the liver and the major oxidation route is conversion to carbamazepine 10,11‐epoxide (CBZE; Figure 1), which is pharmacologically active^2^ and electrophilic.^5^ Other metabolites include 2‐hydroxycarbamazepine (2OH‐CBZ), 3‐hydroxycarbamazepine (3OH‐CBZ),^4^ 2,3‐dihydroxycarbamazepine, and the o‐quinone of the catechol.^2^ Multiple cytochrome P450 (CYP) isoforms are involved in the formation of these metabolites.^4^, ^6^ Other enzymes involved in CBZ metabolism include uridine diphosphate glucuronosyltransferase (UGT2B7),^7^ microsomal epoxide hydrolase and myeloperoxidase.^8^

Clinically, CBZ has a narrow therapeutic index. It induces multiple CYP isoforms and transporters,^9^ and also induces its own metabolism. There is large interindividual variability in plasma levels of CBZ with poor correlation to dose. Patient factors that influence the PK of CBZ include sex, age and total body weight.^10^, ^11^, ^12^ Concomitant medications such as valproic acid, phenytoin, felbamate and phenobarbital have also been associated with variation in metabolism of CBZ.^13^, ^14^, ^15^

CBZ is generally well tolerated, but up to 10% of patients experience a hypersensitivity reaction.^1^ Carriage of HLA‐B*15:02 has been associated with increased risk of CBZ‐induced Stevens–Johnson syndrome (SJS) and toxic epidermal necrolysis (TEN) in patients from South‐East Asia while carriage of HLA‐A*31:01 has been associated with increased susceptibility to all phenotypes of CBZ hypersensitivity in European, Japanese and Korean populations.^16^ Phenytoin is an aromatic anticonvulsant that is also a known cause of SJS/TEN. Similar to CBZ, carriage of HLA‐B*15:02 is associated with increased susceptibility to hypersenstivity.^17^, ^18^ More recently, a genome‐wide association study reported a significant association between phenytoin‐induced serious cutaneous adverse reactions and carriage of CYP2C9*3.^19^ Delayed clearance of plasma phenytoin in patients with severe cutaneous reactions was identified as providing a mechanistic link to the manifestation of hypersensitivity.^20^

Conventionally, it is thought that chemically reactive drug metabolites, such as arene oxides and epoxides, can be cytotoxic or form neo‐antigens with cellular proteins, which activate the immune system, resulting in hypersensitivity reactions.^21^ Recently, however, carbamazepine 10,11‐epoxide has also been shown to have a specific immunogenic effect, namely alteration of selective peptide presentation by HLA‐B*15:02, through binding to the protein, a property not demonstrated by CBZ.^22^

Theoretically, genetic variants in CBZ metabolism could not only alter the routes of metabolism between different individuals, but they could also increase the formation of chemically reactive and otherwise immunogenic metabolites.^23^ Table 1 outlines the studies that have investigated effects of genetic polymorphisms on the metabolism of CBZ. No previous study has comprehensively characterised the effects of genetic variation on metabolism of carbamazepine and its metabolites through PK modelling.

**TABLE 1:** Studies that have investigated single nucleotide polymorphisms associated carbamazepine metabolism

Study reference	Population	No. of subjects	SNP ID	Gene	Nucleotide change/allele	Effect of polymorphism
Ma et al. 2015^24^	Chinese	166	rs2242480	*CYP3A4*	*1G	NS
			rs776746	*CYP3A5*	*3	NS
			rs1051740	*EPHX1*	c.337 T > C	Higher CBZ maintenance dose, lower CBZ natural logarithmic concentration–dose ratio and higher CBZE concentration
			rs7439366	UGT2B7	c.802 T > C	Higher CBZ maintenance dose if also a carrier of *ABCC2* c.1249G > A
			rs3740066	*ABCC2*	c.3972C > T	Higher CBZ maintenance dose and higher CBZE concentration Higher CBZ maintenance dose if also a carrier of *UGT2B7* c.802 T > C
			rs2273697		c.1249G > A	
Wang et al. 2015^25^	Chinese	88	rs4646440	*CYP3A4*	c.1023 + 608C > T	NS
			rs2242480		*1G	NS
			rs15524	*CYP3A5*	*1D	Higher dose adjusted CBZ and CBZE concentration in subjects on concomitant PHT/PB Higher dose adjusted CBZ and CBZE concentration in subjects on concomitant PHT/PB
					*3	
			rs776746			
			rs1045642	*ABCB1*	c.3435C > T	NS CBZ/CBZE ratio in subjects on concomitant PHT/PB CBZ/CBZE ratio in subjects on concomitant PHT/PB NS
			rs2032582		c.2667G > T	
			rs10234411		2,481 + 882A > T	
			rs1128503			
					c.1236 T > C	
Caruso et al. 2014^26^	Italian	50	rs1051740	*EPHX1*	c.337 T > C	NS NS
			rs2234922		c.416A > G	
			rs35599367	*CYP3A4*	*22	NS
Zhu et al. 2014^27^	Chinese	210	rs35599367	*CYP3A4*	*22	NS Lower adjusted levels of CBZ and CBZE
			rs2242480		*1G	
			rs776746	*CYP3A5*	*3	Higher dose adjusted levels of CBZ
			rs1057868	*POR*	*28	NS
			rs2234922	*EPHX1*	c.416A > G	Lower dose adjusted levels of DiOH‐CBZ and DiOH‐CBZ:CBZE ratio Higher DiOH‐CBZ:CBZE ratio NS
			rs3738046		c.128G > C	
			rs1051740		c.337 T > C	
Panomvana et al. 2013^28^	Thai	70	rs776746	*CYP3A5*	*3	Trend towards reduced clearance (not significant)
Puranik et al. 2013^29^	African‐American Caucasian	88	rs2740574	*CYP3A4*	*1B	Reduced clearance
			rs776746	*CYP3A5*	*3	Longer half‐life for CBZ (African Americans)
			rs1051740	*EPHX1*	c.337 T > C	Lower DiOH‐CBZ:CBZE ratio (African‐Americans) NS
			rs2234922		c.416A > G	
			rs7439366	*UGT2B7*	c.802 T > C	NS NS NS Increased clearance (African‐American) NS
			rs11302069		c.1090 + 155delA	
					c.735A > G	
			rs28365062			
					c.372A > G	
					c.1059C > G	
			rs28365063			
			rs4292394			
			rs1045642	*ABCB1*	c.3435C > T	NS Higher clearance (African‐Americans) NS NS Higher DiOH‐CBZ:CBZE ratio (African‐American) Higher DiOH‐CBZ:CBZE ratio (African‐American)
			rs1128503		c.1236 T > C	
			rs2032582		c.2677G > T	
			rs4148734		c.702 + 1789C > T	
			rs4148739		c.2482‐236A > G	
			rs4148740			
					c.2686‐1911 T > C	
			rs2273697	*ABCC2*	c.1249G > A	Higher clearance (Caucasians) Higher CBZE:CBZ ratio (Caucasian male) Reduced clearance and reduced CBZE:CBZ ratio (African‐American) NS
			rs3740066		c.3972C > A	
			rs4148386		208‐3523G > A	
			rs8187710		c.4544G > A	
			rs1523127	*NR1I2*	c.‐131C > A	NS NS Increased CBZE:CBZ ratio and reduced clearance (African‐American) Reduced clearance Increased clearance (African‐American) Increased CBZE:CBZ ratio
			rs1523130		c.‐1,663 T > C	
			rs2461817		c.‐22‐1425A > C	
			rs3814055		c.‐1135C > T	
			rs4688040		c.‐22‐1985G > T	
			rs7643645		c.‐22‐579A > G	
Yun et al. 2013^30^	Chinese	83	rs1051740	*EPHX1*	c.337 T > C	NS Higher adjusted plasma CBZ levels
					c.416A > G	
			rs2234922			
			rs2242480	*CYP3A4*	*1G	NS
Hung et al. 2012^31^	Taiwanese	234	rs1051740	*EPHX1*	c.337 T > C	Higher maintenance dosage NS
			rs2234922		c.416A > G	
			rs7668258	*UGT2B7*	c.‐161 T > C	NS NS NS NS
			rs7438135		c.‐900G > A	
			rs28365062		c.735A > G	
			rs7439366		c.802 T > C	
			rs1128503	*ABCB1*	c.1236 T > C	NS NS NS
			rs2032582		c.2677G > T	
			rs1045642		c.3435C > T	
			rs717620	*ABCC2*	c.‐24C > T	NS NS
					c.3972C > T	
			rs2273697			
Sterjev et al. 2012^32^	North Macedonian	162	rs1045642	*ABCB1*	c.3435C > T	Higher CBZ plasma levels
Meng et al. 2011^33^	Chinese	84	rs1045642	*ABCB1*	c.3435C > T	Lower plasma CBZ levels NS NS
			rs1128503		c.1236 T > C	
			rs2032582		c.2677G > T	
Park et al. 2009^34^	Korean	35	rs776746	*CYP3A5*	*3	Reduced clearance

CBZ: carbamazepine, CBZE: carbamazepine 10,11‐epoxide, DiOH‐CBZ: 10,11‐dihydro‐10,11‐*trans*‐dihydroxy‐carbamazepine, NS: not significant, PB: phenobarbital, PHT: phenytoin

The aim of this study was to develop a population PK model for CBZ and 3 metabolites, namely CBZE, 2OH‐CBZ and 3OH‐CBZ, in healthy adults and adult patients with epilepsy, to generate population clearance estimates for CBZ and the metabolites. Those metabolites were chosen because they are chemically reactive (CBZE) or their generation is via reactive arene oxide intermediates (2OH‐CBZ and 3OH‐CBZ).^5^ The model was used to investigate the influence of physiological factors, concomitant medications, and genetic variation in enzymes and transporters associated with the metabolism and disposition of CBZ.

## 2 METHODS

### 2.1 Ethical approval

Study participants were recruited from 2 different studies: PICME I (Pharmacokinetic Investigation into the formation of Carbamazepine Metabolites and carbamazepine‐protein conjugates in healthy volunteers) and PICME II (Pharmacokinetic Investigation into the formation of Carbamazepine Metabolites and carbamazepine–protein conjugates in epilepsy patients). Both studies were granted clinical trial authorisation by the Medicines and Healthcare products Regulatory Agency in the UK (EudraCT: 2012–004700‐35 and EudraCT: 2013–002743‐28). Ethical approval for PICME I was granted by the North West Research Ethics Committee—Greater Manchester (ethics reference: 12/NW/0780). Ethical approval for PICME II was granted by the North West Research Ethics Committee—Haydock (ethics reference: 13/NW/0503).

### 2.2 Study population

Healthy male adult volunteers were recruited at the Royal Liverpool and Broadgreen University Hospitals NHS Trust, UK, as part of the PICME I clinical study from September 2013 to November 2013. The PICME II clinical study was conducted at the Walton Centre NHS Foundation Trust, UK, from December 2013 to September 2014. Two groups of patient volunteers were recruited as part of the PICME II study: an autoinduction group and a maintenance group. The autoinduction group consisted of patients with epilepsy who were newly initiated on CBZ treatment. The maintenance group consisted of patients with epilepsy who had been established on a stable dose of CBZ for at least 4 weeks. Detailed inclusion and exclusion criteria are available in the supplementary material.

### 2.3 Dosing and sampling schedules

In PICME I, healthy volunteers were administered a single oral dose of 400 mg immediate release CBZ (Novartis Pharmaceuticals UK Ltd., London, UK). Blood samples (5 mL) were taken predose and at 0.25, 0.5, 1, 2, 4, 6, 8, 12, 24, 48 and 72 hours after drug administration. CBZ was administered by study clinicians and nurses, with dosing times recorded in case report forms.

In PICME II, patients with epilepsy in the autoinduction group, who were newly prescribed CBZ, received a single immediate release oral dose of 200 mg (Novartis Pharmaceuticals UK Ltd., London, UK). Blood samples (5 mL) were taken predose and at 1, 2, 4, 6, 8 and 24 hours after drug administration. Subjects were discharged and self‐administered 200 mg CBZ once daily at home. On day 14, subjects returned for further blood sampling predose and then at 1, 2, 4, 6 and 8 hours postdose. After this visit, the dose of CBZ was increased to 200 mg twice daily. On day 28, blood samples (5 mL) were collected at the same time points as on day 14. The dose of CBZ was increased to 200 mg in the morning and 400 mg at night. On day 42, blood samples (5 mL) were collected at the time points given above. This dose titration regimen is standard practice for initiation of CBZ therapy at the Walton Centre NHS Foundation Trust.

Participants in the maintenance group of the PICME II clinical trial underwent sparse PK sampling. Blood samples (5 mL) were collected during routine epilepsy clinic appointments. Postdose timing was calculated by asking the subject the timing and dosage of the previous dose of self‐administered CBZ. All PK blood samples were immediately centrifuged at 1500 *g* for 10 minutes at 4°C. Aliquots of plasma (300 μL) were stored at −80°C until further analysis. Subjects in the maintenance group obtained their CBZ prescription from the community so it was not possible to determine all the manufacturers.

### 2.4 Plasma drug and metabolite assays methods

CBZ and metabolites in plasma samples were assayed by liquid chromatography–tandem mass spectrometry using the method reported in Yip et al.^5^ The calibration ranges for the analytes were as follows: CBZ, 0.33–15.95 μg/mL; CBZE, 0.10–6.30 μg/mL; 2OH‐CBZ, 0.92–118.18 ng/mL; and 3OH‐CBZ, 4.73–605.28 ng/mL. The intraday coefficients of variation for the analytes were as follows: CBZ, 3.7%; CBZE, 3.0%; 2OH‐CBZ, 4.7%; and 3OH‐CBZ, 3.4%. The interday coefficients of variation for each analyte were: CBZ, 6.5%; CBZE, 6.5%; 2OH‐CBZ, 5.5%; and 3OH‐CBZ, 4.0%.

### 2.5 DNA extraction procedure

Blood samples (10 mL) for genetic analyses were collected from all participants at their first study visit and stored at −80°C. DNA was extracted from 4–5 mL of whole blood, thawed at room temperature, using a Chemagic magnetic separation module (Chemagen Biopolymer‐Technologie AG, Baeweiler, Germany) according to the manufacturer's protocol. The concentration of DNA was measured using a NanoDrop microvolume spectrophotometer (Thermo Scientific, Wilmington, USA). The extracted DNA was stored in aliquots at 4°C.

### 2.6 Selection of genetic polymorphisms

The effect of genetic variation on the PK of CBZ was investigated by analysis of single nucleotide polymorphisms (SNPs). The selection of genes and SNPs (Table 2) was based upon the known enzymology of CBZ's metabolism,^4^, ^5^, ^7^, ^46^ efflux transporter activity for CBZ and CBZE,^47^, ^48^ expected minor allele frequencies in the study population (>5%), and a review of previous polymorphism association studies (Table 1). P450 oxidoreductase (POR) supports reactions for all P450 enzymes and its most common variant (POR*28) has been associated with altered activity of CYPs such as CYP2C9^49^ and CYP3A.^50^ A total of 20 SNPs were chosen in 11 genes (Table 2).

**TABLE 2:** Selection of genes and single nucleotide polymorphisms for genotyping studies

SNP ID	Gene	Nucleotide change/allele	Potential change in function	SNP typing platform	References
rs1045642	*ABCB1*	3435C > T	Reduced expression	Taqman	^35^
rs1128503	*ABCB1*	1236 T > C	Inconclusive	MassArray	^35^
rs2032582	*ABCB1*	2677G > T	Inconclusive	MassArray	^35^
rs2273697	*ABCC2*	1249G > A	Increased activity	MassArray	^36^
rs3740066	*ABCC2*	3972C > T	Inconclusive	MassArray	^36^
rs717620	*ABCC2*	–24C > T	Reduced activity	Taqman	^36^
rs28365062	*UGT2B7*	735A > G	Increased activity	MassArray	^37^
rs28365063	*UGT2B7*	372A > G	Increased activity	MassArray	^29^
rs7438135	*UGT2B7*	–900G > A	Increase activity	MassArray	^38^
rs1051740	*EPHX1*	337 T > C	Increased activity	MassArray	^39^
rs2234922	*EPHX1*	416A > G	Reduced activity	MassArray	^39^
rs11572080	*CYP2C8*	*3	Inconclusive	MassArray	^40^
rs1058930	*CYP2C8*	*4	Reduced activity	MassArray	^40^
rs4244285	*CYP2C19*	*2	Loss of function	MassArray	^41^
rs192154563	*CYP2C19*	*17	Increase activity	MassArray	^41^
rs3745274	*CYP2B6*	*6	Reduced activity	Taqman	^42^
rs2242480	*CYP3A4*	*1G	Increased activity	MassArray	^27^
rs776746	*CYP3A5*	*3	Loss of function	MassArray	^43^
rs2333227	*MPO*	–463G > A	Reduced activity	Taqman	^44^
rs1057868	*POR*	*28	Increased activity	Taqman	^45^

### 2.7 Analysis of single nucleotide polymorphisms

SNPs were typed using either the Agena MassArray iPLEX platform (Agena Inc, San Diego, CA, USA) or manufacturer‐validated TaqMan real‐time PCR SNP genotyping assays (Life Technologies, Paisley, UK) according to the manufacturer's protocols (Table 2). The genotype frequencies of each SNP were examined for deviation from Hardy–Weinberg equilibrium (HWE) using the χ^2^ test.

### 2.8 Population PK modelling

**FIGURE 2:**
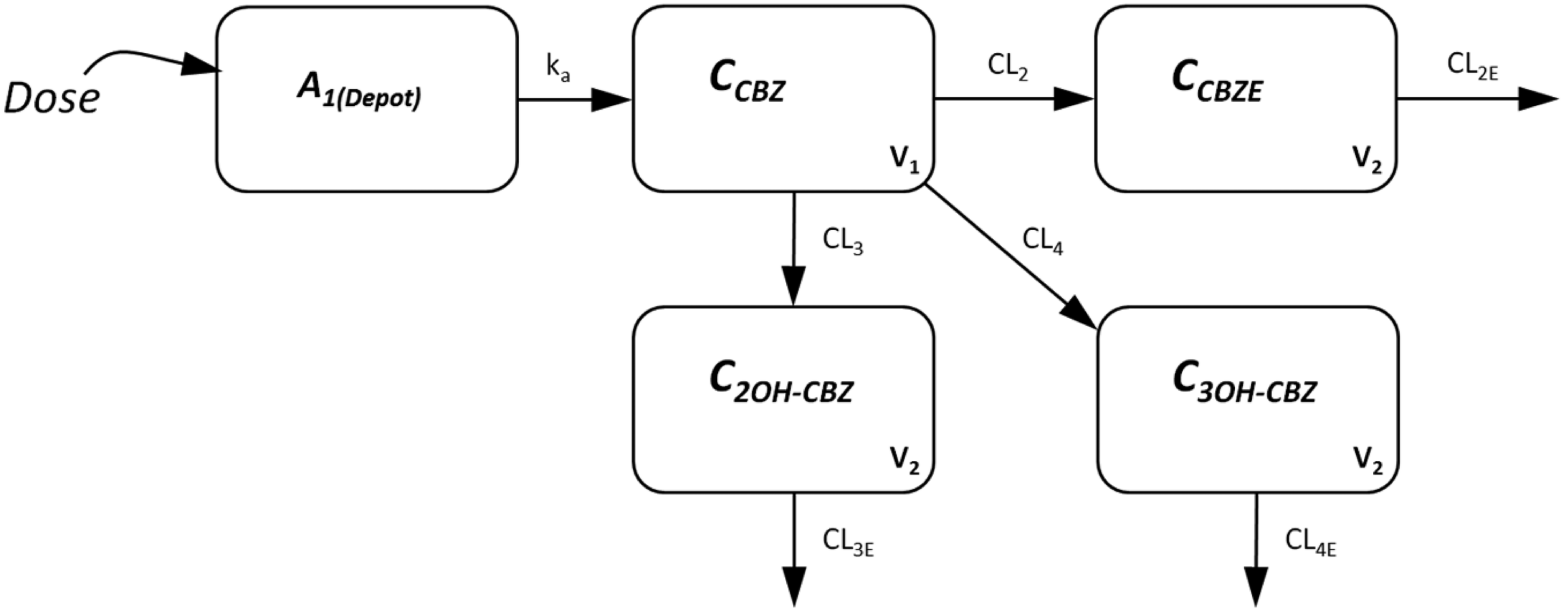
Schematic of the pharmacokinetic model for carbamazepine

Equations 1–9 are the final ordinary differential equations and associated (re)parameterisations for the structural PK model applied to concentration data from simultaneous assays of CBZ and the 3 metabolites in plasma. A schematic of the final model is given in Figure 2.

(1) $${\mathrm{dA}}_{1}/\mathrm{dt}=-\mathrm{KA}*A_{1}\;\text{dose oral dose depot amount}$$

(2) $${\mathrm{dC}}_{\mathrm{CBZ}}/\mathrm{dt}=\mathrm{KA}*A_{1}/V_{1}\;-\;{\mathrm{CL}}_{\mathrm{TOT}}*C_{\mathrm{CBZ}}/V_{1}\;\mathrm{CBZ}\;\text{plasma concentration}$$

(3) $${\mathrm{dC}}_{\text{CBZE}}/\mathrm{dt}={\mathrm{CL}}_{2}*C_{\mathrm{CBZ}}/V_{2}-{\mathrm{CL}}_{\text{2E}}*C_{\text{CBZE}}/V_{2}\;\text{CBZE plasma concentration}$$

(4) $${\mathrm{dC}}_{\text{2OH}-\mathrm{CBZ}}/\mathrm{dt}={\mathrm{CL}}_{3}*C_{\mathrm{CBZ}}/V_{2}-{\mathrm{CL}}_{\text{3E}}*C_{\text{2OH}-\mathrm{CBZ}}/V_{2}\;\text{2OH}-\mathrm{CBZ}\;\text{plasma concentration}$$

(5) $${\mathrm{dC}}_{\text{3OH}-\mathrm{CBZ}}/\mathrm{dt}={\mathrm{CL}}_{4}*C_{\mathrm{CBZ}}/V_{2}-{\mathrm{CL}}_{\text{4E}}*C_{\text{3OH}-\mathrm{CBZ}}/V_{2}\;\text{3OH}-\mathrm{CBZ}\;\text{plasma concentration}$$

Where:

(6) $${\mathrm{CL}}_{2}={\mathrm{CL}}_{\mathrm{TOT}}*\left(1-{\mathrm{FM}}_{1}\right)$$

(7) $${\mathrm{CL}}_{3}={\mathrm{CL}}_{\mathrm{TOT}}*{\mathrm{FM}}_{1}/2$$

(8) $${\mathrm{CL}}_{4}={\mathrm{CL}}_{\mathrm{TOT}}*{\mathrm{FM}}_{1}/2$$

(9) $$\mathrm{KA}={\mathrm{CL}}_{\mathrm{TOT}}/V_{1}\;+{\mathrm{KA}}_{\text{star}}$$

A 1‐compartment PK model with first‐order absorption and elimination was chosen for CBZ based on visual inspection of the raw data and previously published analyses of the PK of CBZ. Exploratory analyses involved 1‐compartment fitting to parent CBZ data alone to provide initial estimates for the final fitting. To account for CBZE, 2OH‐CBZ and 3OH‐CBZ metabolite production, the total clearance of CBZ (CL_TOT_) was partitioned into CL_2_, CL_3_, and CL_4_, respectively, via the FM_1_ parameter (Equations 6–8) and under the assumptions that production of the 3 observed metabolites accounted for approximately all the primary elimination of CBZ, and that CL_3_ equals CL_4._ ^3^, ^4^, ^51^ CBZE, 2OH‐CBZ and 3OH‐CBZ were all subject to first‐order elimination governed by CL_2E_, CL_3E_ and CL_4E_ parameters, respectively, with the same volume of distribution (V_2_) estimated for the 3 metabolites. To ensure identifiability of the model, FM_1_ was fixed to a value of 0.64 according to prior mass balance information regarding the metabolic fate of CBZ (i.e. 36% of parent CBZ is primarily converted to CBZE),^51^, ^52^ and, in addition, the absorption rate constant for CBZ (KA) was constrained to be greater than the elimination rate constant for CBZ by estimating a KA_star_ parameter and the use of Equation 9. Assuming the same volume of distribution was also necessary for the identifiability of the model and was deemed reasonable given the metabolites are isomerically similar and have similar physicochemical properties. The structural identifiability of the model was confirmed (7 PK parameters estimated: KA_star_, CL_TOT_, CL_2E_, CL_3E_, CL_4E_, V_1_, V_2_; 4 analytes/states observed) using the DAISY software package,^53^ and in the absence of IV data, estimates of CL and V parameters in the model are apparent in nature, i.e. CL/F, V/F etc. A schematic of the final model is provided in Figure 2.

Population PK analysis of the data for CBZ and its metabolites was performed with NONMEM (version 7.3, ICON plc, South County Business Park, Leopardstown, Dublin, Republic of Ireland), using the first‐order conditional estimation method (with interaction). Interindividual variability (assuming a log‐normal distribution across the population) was included in each of the structural model parameters, while residual error was accounted for using an exponential error model, with a separate residual error variance estimated for each of the 4 plasma analytes. In addition, to achieve shorter computational run times and improved model stability, the closed form analytical solutions of the model's differential equations were derived and implemented for model fitting, with multiple dosing super‐positioning (according to each individual dosing record) implemented via the DOWHILE/ILOOP functionality in NONMEM 7.4 abbreviated code. Subjects in the PICME II maintenance group were modelled with an assumed dosing record of 8 weeks BID dosing (at each individual's specific dose level) to replicate steady state conditions. The analytical solutions of the model equations are provided in the supplementary information.

The known auto‐induction effect of CBZ, was incorporated in the base model as a binary, categorical *induction status effect* on CL_TOT_ and CL_3E_. Data from PICME I and the first visit of the PICME II autoinduction group were categorised as having come from a *noninduced* period while data from the second visit of the PICME II autoinduction group and the PICME II maintenance group were categorised as from an *induced* period (i.e. a binary, noncontinuous time function)—reflecting the length of CBZ dosing up to the point of PK observation, with the latter groups having received at least 14 days daily dosing of CBZ, which would be expected to increase clearance. More sophisticated models for autoinduction as a *continuous* function of time (and drug exposure, taking the form of e.g. an autoinduction enzyme turnover model used previously for rifampicin were attempted in exploratory analysis, but failed to minimise successfully and/or give acceptable parameter estimate precision.^54^ We believe that this is at least in part due to the dataset lacking information on a continuous range of levels of induction over time among the patients, which a turnover autoinduction model might need for its parameters to be sufficiently well estimated: patient PK in this study was observed under the more binary condition of either showing the effects of induction after several weeks dosing or not. Exploratory analyses went on to test the induction effect on CL_TOT_, FM1 and metabolite clearances (CL_2E,3E,4E_) with the final base model retaining the effect on CL_TOT_ and CL_3E_, expressions for which are given in Equations 10 and 11, with *IND* as a dummy variable equal to 1 or 0 for an *induced* or *noninduced* period datapoint, θ_x_ the fixed effect values for the parameter or the induction effect in question, and η_x_ the log interindividual variability random effect component, drawn from a normal distribution with mean of 0 and variance ω_x_^2^. Data from PICME I and PICME II autoinduction visit 1 were IND = 0, while data from PICME II autoinduction visit 2 and PICME II maintenance were IND = 1. Further equations for covariate models are expressed in *typical value* format, omitting the random effect component for clarity.

(10) CLTOT,i=θCLTOT*θIND.EFFCT1^INDi*expηCLTOT,i

(11) CL3E,i=θCL3E*θIND.EFFCT2^INDi*expηCL3E,i

Exploratory analyses investigating incorporation of interoccasion variability as a random effect in the model failed to produce acceptable fittings, despite the potential for this to be required to describe the PICME II autoinduction group data in particular, where patients' PK was monitored on 2 occasions.

Age and weight were investigated as continuous covariates in the model on CL_TOT_, V_1_ and metabolite clearances (CL_2E,3E,4E_) using expressions of the form in Equation 12, where individual covariate values (cov_i_) were normalised to the population means (45.8 years for age or 80.8 kg for weight).

(12) TVθi=θpop*covi/covmean^θCOV

The daily dose of CBZ (DOSE) was investigated as a covariate on CL_TOT_ in a similar manner (Equation 13, where 400 mg was the most common daily dose) to allow, for example, for a potentially greater CL_TOT_ in patients on higher doses due to greater induction of metabolism.

(13) TVCLTOT_i=θCLTOT+lnDOSEi/400^θCOV

Smoking status, and whether or not a patient was administered a specific comedication, were examined as categorical covariate effects on CL_TOT_ using an expression of the form in Equation 14, with MED as an indicator equal to 1 or 0 if the comedication was present or absent, respectively. Comedications investigated included valproate, phenytoin, phenobarbital, clobazam, lamotrigine, levetiracetam, zonisamide, topiramate, lacosamide, pregabalin, perampanel, omeprazole, atorvastatin, pravastatin and simvastatin.

(14) TVCLTOT_i=θCLTOT*θCOV^MED

The potential effects of the SNPs genotyped in the subject population were generally examined as categorical covariate effects on CL_TOT_, using an expression of the form in Equation 15 with 2 θ_COV_ parameters (2 extra degrees of freedom) to allow characterisation of heterozygous mutant, *vs* homozygous mutant, *vs* wild type status. SNP_HOM_ and SNP_HET_ are dummy variables equal to 1 or 0 to record the genotype status as yes/no, respectively, with both equal to 0 for wild‐type individuals.

(15) TVCLTOT_i=θCLTOT*θCOV_HOM^SNPHOM*θCOV_HET^SNPHET

The exceptions to equation 14 among the SNPs investigated were the EPHXTC and EPHXAG genotypes (Table 2), which were investigated for potential effects on CL_2E_ as these genes are specifically associated with the metabolic clearance of CBZE.

SNP covariate effects were also examined for effect of homozygous mutant only *vs* combined wild type/heterozygous mutant group and for either homozygous or heterozygous mutant *vs* wild type. These analyses required only a single θ_COV_ degree of freedom parameter:

(16) TVCLTOT_i=θCLTOT*θCOV_HOM^SNPMUT

Covariate model selection adopted a standard forward addition/backward deletion approach.^55^ The covariates were initially examined in univariate analyses as single additions to the base model. They were deemed statistically significant (*P* < .05) based on a drop in objective function value compared to the base model of 3.8 or 6.0 (or greater) for 1 or 2 degrees of freedom, respectively. In terms of extra covariate effect parameters added to the model, −2 degrees of freedom were required, e.g. for genotype HOM *vs* HET *vs* WT covariate models. Final covariate model selection via backward deletion adopted a statistical significance criterion of *P* < .01, i.e. changes in objective function value of 6.6 or 9.2 for 1 or 2 degrees of freedom, respectively.

Standard goodness‐of‐fit plots (observed concentrations *vs* population‐ and individual‐predicted concentrations, as residual plots) and a prediction‐corrected visual predictive check (VPC, based on 1000 simulations of the dataset population) were used to evaluate the final model fitting to the dataset, with parametric standard errors (expressed as percentage relative standard errors) used to assess the precision of model parameter estimates.

### 2.9 Nomenclature of targets and ligands

Key protein targets and ligands are hyperlinked to corresponding entries in http://www.guidetopharmacology.com,^56^ and are permanently archived in the Concise Guide to Pharmacology 2019/20.^57^

## 3 RESULTS

### 3.1 Subject demographics and genetic polymorphisms

In total, 80 subjects were recruited into the studies (Table 3). Rich PK sampling data were obtained from 8 healthy volunteers who completed the PICME I study. Rich PK sampling data were also obtained from 3 patients with a new diagnosis of epilepsy who completed dose titration of CBZ as part of the autoinduction group of PICME II. Eighty sparse PK samples were collected from 69 patients who were recruited to the maintenance group of PICME II. In total, 248 sets of drug and metabolite assays were used in the analysis.

**TABLE 3:** Demographic and medication data for subjects included in population PK analysis. Data are expressed as mean values ± standard deviation (ranges)

	PICME I: Healthy volunteer	PICME II: Autoinduction group	PICME II: Maintenance group
Numbers of subjects	8	3	69
Sets of drug/metabolite assays	96	75	77
Gender (%):			
Male	8 (100%)	1 (33.3%)	37 (53.6%)
Female	0	2 (66.6%)	32 (46.4%)
Age (y)	27.4 ± 3.5 (21.7–32.8)	46.7 ± 4.4 (43.5–51.7)	47 ± 14.3 (25.9–75.8)
Total body weight (kg)	84.6 ± 11.2 (69.8–99.4)	95.2 ± 26.4 (73.1–124.5)	79.7 ± 18.8 (39.0–140.5)
Smoker (%):			
Yes	0 (0%)	0 (0%)	20 (29.0%)
No	8 (100%)	3 (100%)	49 (71.0%)
Sparse PK sampling time points (minutes)	NA	NA	337.5 ± 230.9 (70–1030)
CBZ dose (mg/d)	400‐mg single dose	200 mg once daily (days 1–14)	819 mg ± 367 (range: 200–1600 mg)
		200 mg twice daily (days 15–28)	
		200 mg AM and 400 mg PM (days 29–42)	
CBZ therapy without concomitant AEDs or CYP3A4 inducer/inhibitor (%)	8 (100%)	2 (66.6%)	16 (23.2%)
Concomitant with 1 AED (%)	0	0	26 (37.7%)
Concomitant with 2 AEDs (%)	0	0	16 (23.2%)
Concomitant with 3 AEDs (%)	0	0	5 (7.2%)
Concomitant with 1 CYP3A4 inducer/inhibitor (%)	0	1 (33.3%)	12 (17.4%)
Concomitant with 2 CYP3A4 inducer/inhibitors (%)	0	0	3 (4.3%)
Number of subjects for each AED:			
Levetiracetam	0	0	26
Clobazam	0	0	18
Lamotrigine	0	0	5
Zonisamide	0	0	5
Phenytoin	0	0	4
Topirmate	0	0	4
Pregabalin	0	0	4
Valproate	0	0	3
Lacosamide	0	0	3
Perampanel	0	0	1
Phenobarbital	0	0	1
Number of subjects for each CYP3A4			
Inducer/inhibitor:			
Statin	0	1	12
Omeprazole	0	0	6

* time points applicable only to sparse PK sampling group; AED: antiepileptic drug; CBZ: carbamazepine, PK: pharmacokinetic.

The distribution of the 20 SNPs amongst these subjects is recorded in Table 4. No subjects were carriers for CYP2C19*17 (rs192154563). Each genotype frequency was consistent with HWE, and minor allele frequencies ranged from 4 to 55% in keeping with polymorphism frequencies reported in the literature. No demographic covariates were significant in the final PK model.

**TABLE 4:** Distribution of single nucleotide polymorphisms (SNPs) amongst study subjects (*n* = 80)

SNP ID	Gene	Allele	Genotype frequency (%)			Minor allele frequency	Hardy–Weinberg equilibrium (*P*)
			Wild type	Heterozygote	Homozygote		
rs1045642	*ABCB1*	3435C > T	14 (17.5%)	44 (55%)	22 (27.5%)	0.55	.32
rs1128503	*ABCB1*	1236 T > C	14 (17.5%)	42 (52.5%)	24 (30%)	0.56	.55
rs2032582	*ABCB1*	2677G > T	24 (30.0%)	39 (48.8%)	17** (21.2%)	0.46	.88
rs2273697	*ABCC2*	1249G > A	49 (61.3%)	29 (36.3%)	2 (2.4%)	0.21	.34
rs3740066	*ABCC2*	3972C > T	25 (31.3%)	42 (52.5%)	13 (16.2%)	0.43	.51
rs717620	*ABCC2*	–24C > T	50 (62.5%)	26 (32.5%)	4 (5.0%)	0.21	.80
rs28365062	*UGT2B7*	735A > G	62 (77.5%)	18 (22.5%)	0 (.0%)	0.11	.26
rs28365063	*UGT2B7*	372A > G	56 (70.0%)	24 (30.0%)	0 (.0%)	0.15	.11
rs7438135	*UGT2B7*	–900G > A	27 (33.8%)	39 (48.7%)	14 (7.5%)	0.42	.99
rs1051740	*EPHX1*	337 T > C	36 (45.0%)	37 (46.3%)	7 (8.7%)	0.32	.56
rs2234922	*EPHX1*	416A > G	53 (66.3%)	24 (30.0%)	3 (3.7%)	0.19	.89
rs11572080	*CYP2C8*	*3	62 (77.5%)	16 (20.0%)	2 (2.5%)	0.13	.44
rs1058930	*CYP2C8*	*4	70 (87.5%)	10 (12.5%)	0 (.0%)	0.06	.55
rs4244285	*CYP2C19*	*2	57 (71.3%)	19 (23.7%)	4 (5.0%)	0.17	.17
rs192154563	*CYP2C19*	*17	80 (100%)	0 (0.0%)	0 (.0%)	0.00	NA
rs3745274	*CYP2B6*	*6	52 (65.0%)	26 (32.5%)	2 (2.5%)	0.19	.55
rs2242480	*CYP3A4*	*1G	71 (88.8%)	9 (11.2%)	0 (.0%)	0.06	.59
rs776746	*CYP3A5*	*3	73 (91.3%)	7 (8.7%)	0 (.0%)	0.04	.68
rs2333227	*MPO*	–643G > A	12 (15%)	41 (51.3%)	27 (33.7%)	0.59	.58
rs1057868	*POR*	*28	40 (50.0%)	27 (33.8%)	13 (16.2%)	0.33	.53

** included 3 TA subjects, NA: not applicable.

### 3.2 Base model and covariate fitting

**FIGURE 3:**
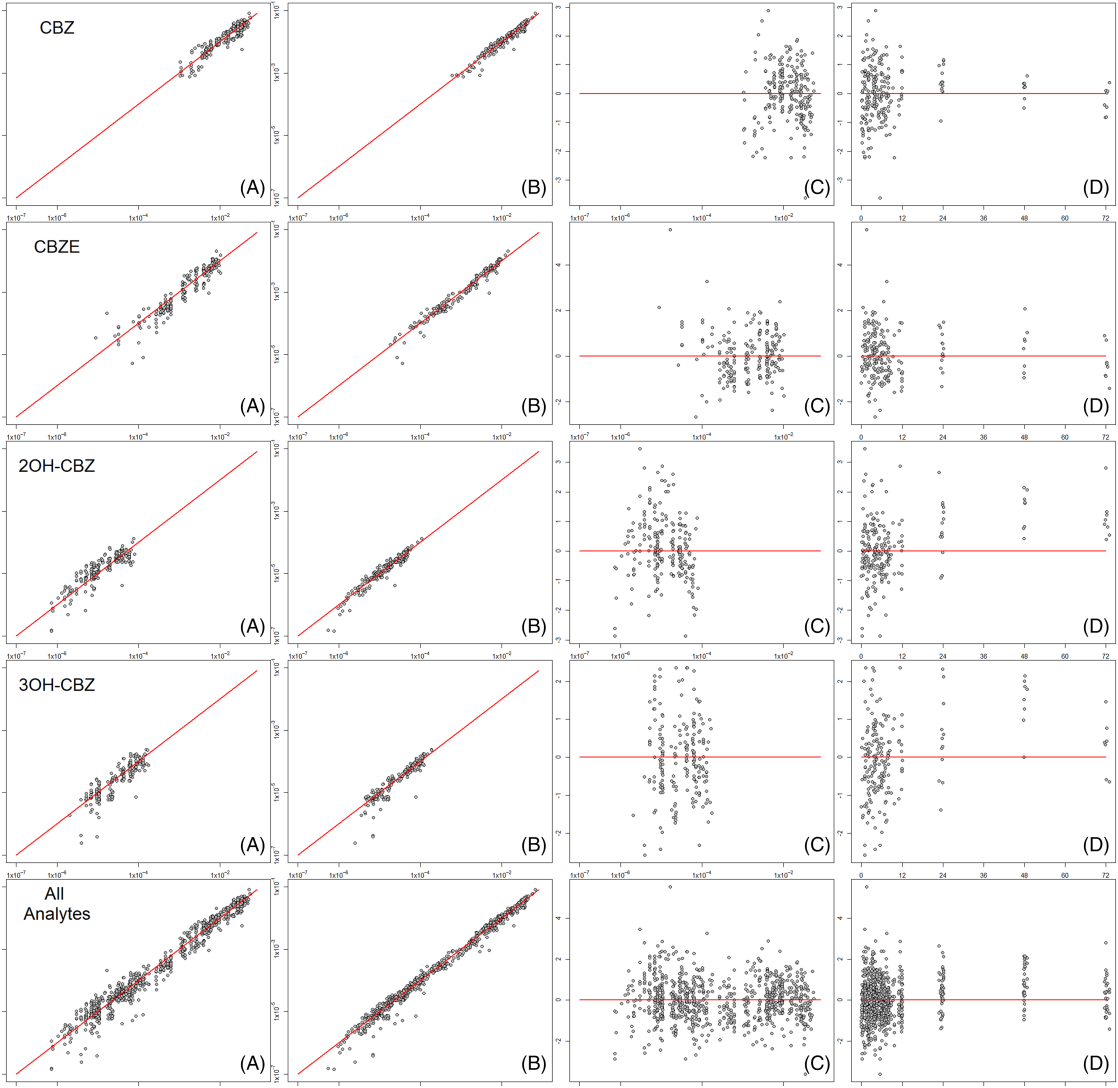
Goodness of fit diagnostic plots for of carbamazepine and metabolites (as labelled by row): (A) Observed concentrations (mM) *vs* population predicted concentrations (mM); (B) Observed concentrations (mM) *vs* individual predicted concentrations (mM); (C) Conditional weighted residuals *vs* population predicted concentrations (mM); (D) Conditional weighted residuals *vs* time after dose (h)

**FIGURE 4:**
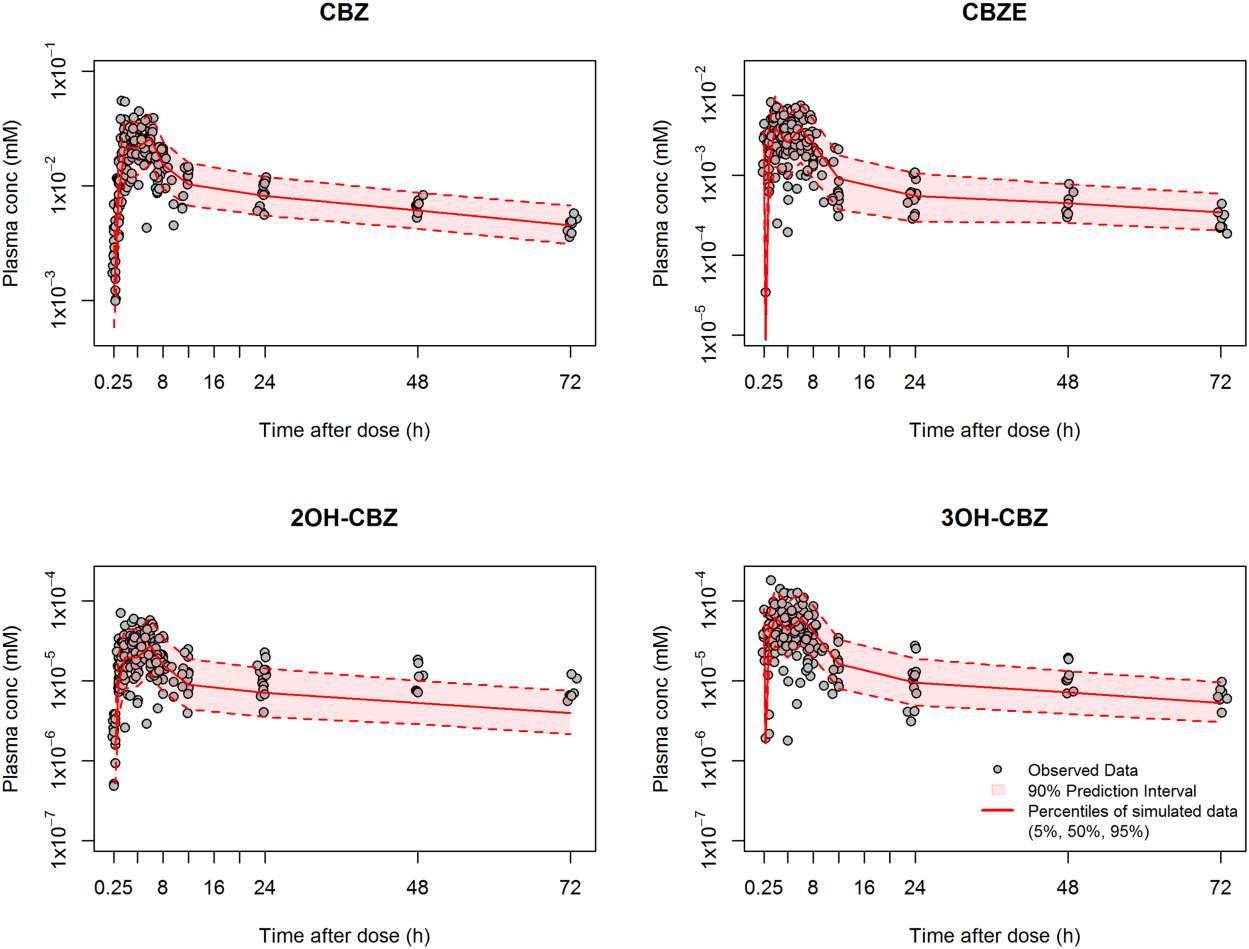
Prediction‐corrected visual predictive check for the final pharmacokinetic model fitting for each of the analytes. 90% prediction interval (broken line) and median population prediction (continuous line) determined from 1000 simulations for CBZ with the covariate values of those individuals used in the model building process. CBZ: carbamazepine; CBZE: carbamazepine‐10,11‐epoxide; 2OH‐CBZ: 2‐hydroxy‐carbamazepine; 3OH‐CBZ ‐ 3‐hydroxy‐carbamazepine

As illustrated by the VPC and diagnostic plots (Figures 3 and 4), the PK profiles for CBZ and its 3 assayed metabolites, and their variability, were adequately described by the applied final model. VPC plots stratified according to the study group (PICME I, PICME II autoinduction, and PICME II maintenance) are provided in the Supplementary Material with plots of raw data and mean observed profiles where applicable. There was, however, some apparent underprediction of 2OH‐CBZ concentrations at time points later than 24 h. These 48 and 72 h timepoints all originate from the 8 patients of the PICME I study group, with the majority of the exposure dataset coming from the 72 patients of PICME II (autoinduction and maintenance); it is feasible therefore that the apparent poor fit at 48 h and 72 h for 2‐OH reflects that the overall population fitting is weighted to the description of the 90% of overall subjects in the PICME II study groups, where a higher CL3E is more typical, as accounted for at least in part by the 1.7‐fold increased induction effect on CL3E retained in the final model. The result would be a higher population CL3E estimate than is ideal to describe the late timepoints of PICME I profiles; however, with the PICME II subjects in turn having no 48‐ and 72‐h observations to balance out the VPC plot at the later timepoints, the apparent underprediction can be accounted for then as a result of the compromise resulting from the combined analysis of the complete dataset.

The population mean estimates for CL_TOT_, V1 and KA of CBZ are provided in Table 5. The CL_TOT_ value in our model was 1.96 L/h which falls in the centre of previously published CL_TOT_ values which range between 1.15 and 3.58 L/h.^10^, ^11^, ^13^, ^14^ The CL value for CBZE in our model was 9.71 L/h. In a previous population PK model, which included CBZE the CL estimate for CBZE in a 70 ‐kg patient prescribed 400 mg CBZ was 28 L/h.^58^ A significant part of the discrepancy can be explained by the assumed conversion ratio between CBZ and CBZE. Our model assumes that 0.36 of CBZ is converted to CBZE^51^, ^59^ whilst the model of Jiao et al. assumes that 0.65 of CBZ is converted to CBZE.

**TABLE 5:** Final model parameters

Parameter	Estimated value [%RSE] (EPS‐shrinkage %)		% Interindividual variability [%RSE] (ETA‐shrinkage %)	
CL_TOT_ (L/h)	1.96	[18]	25.3	[23] (20)
FM_1_	0.64	(fixed)	6.3	[62] (65)
CL_2E_ (L/h)	9.71	[5]	27.9	[16] (29)
CL_3E_ (L/h)	726	[25]	42.3	[16] (14)
CL_4E_ (L/h)	543	[6]	34.5	[14] (23)
KA_STAR_ (h^−1^)	0.409	[24]	75.3	[19] (63)
V_1_ (L)	161	[7.0]	13.6	[27] (22)
V_2_ (L)	39.2	[45]	149.7	[30] (64)
CL_TOT_ induction effect	1.73	[19]	‐	‐
CL3E induction effect	1.63	[27]	‐	‐
Cov.Eff.(DOSE on CL_TOT_)	1.36	[10]	‐	‐
Cov.Eff.(EPHXAG_HOM_ on CL2E)	0.502	[14]		
Cov.Eff.(PHT on CL_TOT_)	1.93	[25]	‐	‐
CBZ resid err.Sd	0.221	[10] (12)	‐	‐
CBZE resid err.Sd	0.310	[6.0] (12)	‐	‐
2OH‐CBZ resid err.Sd	0.297	[8.0] (13)	‐	‐
3OH‐CBZ resid err.Sd	0.362	[12.0] (11)	‐	‐

Cov.eff: covariate effect; FM: metabolite fraction; PHT: phenytoin; %RSE: percentage relative standard error

A summary of relevant computational runs for building the final covariate model is given in Table 6, with the final model including the effects of total daily dose (DGRP), EPHX1‐416G/G (homozygous mutant) genotype and coadministration of phenytoin as significant covariates on CBZ total clearance. None of the other SNP genotypes investigated showed a statistically significant effect on clearance of either CBZ or CBZE during the backward deletion component of model selection. A table of changes in objective function following incorporation of the various covariates investigated during model building is provided in the Supplementary Material.

**TABLE 6:** Covariate model building

Covariate model	Objective function value	Difference in objective function value
Base model	−14 739.0	
DOSE on CL_TOT_	−14 804.3	−65.3
EPHXAG_HOM_ on CL_2E_	−14 747.7	−8.7
PHT on CL_TOT_	−14 744.3	−5.3
DOSE on CL_TOT_ & EPHXAG_HOM_ on CL_2E_	−14 813.0	−8.6
DOSE & PHT on CL_TOT_	−14 810.2	−5.9
DOSE & PHT on CL_TOT_ & EPHXAG_HOM_ on CL_2E_	−14 819.2	−6.2

PHT: phenytoin

The final model equation for an individual subject's CL_TOT_ and CL_3E_ are given in equations 17 and 18:

(17) CLTOTi=θCLTOT*θIND.EFFCT1^OCCi*θCOV_PHT^PHTi+lnDOSEi/400^θCOV_DOSE*expηCLTOT,i

(18) CL3E,i=θCL3E*θCOV_MUT^EPHXAGHOM*expηCL3E,i

## 4 DISCUSSION

A population PK model was developed to investigate the effects of SNPs in genes involved with metabolism and efflux transport of CBZ on the plasma PK of CBZ and 3 metabolites in healthy volunteers and epilepsy patients. The influence of demographic and clinical characteristics of the patients on the clearance of CBZ was also examined. The model revealed that the *EPHX1*‐416G/G genotype was a significant covariate for the clearance of CBZE. Concomitant therapy with phenytoin and the total daily dose of CBZ were also significant covariates for the clearance of CBZ. However, none of the other SNPs investigated exerted a demonstrable effect on CBZ PK.

The gene *EPHX1* encodes microsomal epoxide hydrolase, which catalyses hydrolysis of the electrophilic CBZE to DiOH‐CBZ.^6^ The SNP *EPHX1 c.416A > G* is associated with reduced epoxide hydrolase activity.^39^ Three subjects in our model possessed the *EPHX1*‐416G/G genotype. When compared with *EPHX1*‐416G/A and ‐416A/A genotype subjects, their clearance of CBZE was reduced by approximately 50%. CBZE retains anticonvulsant activity but elevated plasma levels, secondary to concurrent brivaracetam therapy, have been associated with reduced tolerability to CBZ treatment and an increase in adverse events such as blurred vision, dizziness and fatigue.^60^ Brivaracetam inhibits microsomal epoxide hydrolase and led to a rise in CBZE levels by a median of 83.9% (range 57.9–341.7%), which highlights that a 50% reduction in clearance that has been associated with *EPHX ‐416G/G* genotype may also be clinically relevant. One previous investigation reported that patients with the *EPHX c.416A > G* SNP had higher adjusted plasma CBZ levels^30^ whilst another identified lower adjusted levels of DiOH‐CBZ^27^ in patients receiving CBZ. However, 3 studies were unable to detect differences between plasma levels of either CBZ or its metabolites in patients with the *EPHX1 c. 416A > G* polymorphism.^26^, ^29^, ^31^

A second SNP in *EPHX1*, namely *c.337 T > C*, was not associated with altered clearance of CBZE in our model. A study in Han Chinese patients with CBZ‐induced SJS/TEN found a significant association between *EPHX1 c.337 T > C* polymorphisms and the development of SJS/TEN.^61^ The authors postulate that the polymorphism may contribute to the risk of CBZ‐induced SJS/TEN by increasing the plasma concentration of CBZE. The same SNP in *EPHX1* was associated with increased levels of CBZE in 1 study^24^ but no differences were detected in the current study or in studies by others.^26^, ^27^, ^30^ In our model, SNPs in genes of CYPs, other enzymes (including UGT2B7, generally regarded as a detoxification enzyme) and drug transporters were not significant covariates of the PK of CBZ.

The influence of phenytoin therapy on CBZ's metabolism has been recognised in several other population PK models.^11^, ^12^, ^13^, ^15^ Phenytoin increases the metabolism of many drugs, including immunosuppressants,^62^ chemotherapeutic agents^63^ and antiretroviral drugs,^64^ through induction of multiple CYP isoforms and upregulation of P‐gp.^65^ Patients who are coprescribed CBZ with phenytoin potentially require larger doses of CBZ to maintain plasma levels.

The total daily dose of CBZ was another significant covariate in the population PK model. All subjects in the study were prescribed controlled release formulations of CBZ. There was a positive correlation between total daily dose and clearance of the drug. The effect of dose on clearance may be explained by a reduction in bioavailability and increase in clearance through greater autoinduction at higher doses.^66^ Several other population PK models of CBZ have reported that the total daily dose is a significant covariate.^10^, ^67^, ^68^, ^69^, ^70^

Concomitant treatments with sodium valproate,^11^, ^12^, ^14^, ^69^ phenobarbital^11^, ^12^, ^13^, ^67^, ^69^, ^71^ and felbamate^13^ have been reported to be significant covariates in population PK models of CBZ. None of those antiepileptic drugs (AEDs) achieved a significant effect in the current model. This is most likely to have been due to the small numbers of subjects receiving any of these drugs. No other AEDs were significant in our model. Concomitant treatment with omeprazole (*n =* 6) and statins (*n =* 13) were also investigated as covariates in the population PK model. Omeprazole is a proton‐pump inhibitor that is associated with drug–drug interactions secondary to its inhibition of CYP2C19 and CYP3A4.^72^ Statins have been reported to inhibit CYP enzymes,^73^ and they act as substrates of the organic anion transporters OATP1B1 and OATP1B3 and of P‐gp.^74^ Neither was found to affect metabolism of CBZ significantly.

There is considerable uncertainty regarding the influence of genetic variation on metabolism of CBZ, with conflicting results from several studies (Table 1). Up to 1/3 of patients with epilepsy do not respond to AED therapy and the transporter hypothesis proposes that over expression of efflux transporters such as ABCB1 in the blood–brain barrier limits access of AEDs to the epileptic focus.^75^ Earlier studies have reported the requirement for higher CBZ maintenance doses,^24^ increased clearance of CBZ^29^ as well as both higher^32^ and lower^33^ plasma levels of CBZ in patients with SNPs in *ABCB1* and *ABCC2*. In our population PK model 6 SNPs in the genes *ABCB1* and *ABCC2*, which both encode drug efflux transporters, did not demonstrate a significant effect on the PK of CBZ.

CYP3A4 is the major CYP isoform responsible for oxidative metabolism (aromatic hydroxylation and 10,11‐epoxidation) of CBZ.^4^, ^76^ The allele CYP3A4*1G has been associated with lower serum levels of CBZ and CBZE in Chinese patients with epilepsy.^27^ However, 3 other analyses were not able to replicate this association,^24^, ^25^, ^30^ and our own population PK model did not detect a significant association between SNPs in CYP isoforms and clearance of CBZ. It is possible that the multiple enzymatic pathways involved in CBZ metabolism,^5^ and the multiplicity of CYP isoforms catalysing each of those pathways,^4^, ^5^ may compensate for the altered functioning of the gene for a particular enzyme, thereby limiting the effect on total drug clearance of individual SNPs. Furthermore, most of the subjects in our study who carried a variant were heterozygotes; their functional allele will have limited the consequences of the polymorphism. Collectively, the effect of the PK gene variants investigated in this study might be relatively small, and thus much larger sample sizes would be required to detect the effect.

HLA‐B*15:02 and HLA‐A*31:01 are recognised as biomarkers for susceptibility to CBZ‐induced hypersensitivity reactions.^16^ However, carriage of these *HLA* alone may be insufficient to trigger a hypersensitivity reaction. A recent experimental study has demonstrated that it is CBZE, and not CBZ, which binds to the HLA‐B*15:02 molecule and alters its peptide‐binding motif for B*15:02‐restricted peptides.^22^ It is possible that susceptibility to CBZ hypersensitivity reactions is dependent on the presence of specific immunogenetic factors, such as carriage of a risk *HLA* genotype, and combinations of allelic enzyme variants in the metabolic pathways of CBZ that generate above threshold levels of immunoreactive metabolites. For example, 1 study reported that patients required higher dosages of CBZ only if they possessed a combination of SNPs in both *UGT2B7* and *ABCC2*.^24^ Similarly, combined *HLA* and CYP2C9*3 pharmacogenetic screening for phenytoin hypersensitivity improved the sensitivity and specificity of predictive testing.^20^

We have previously reported that chemically reactive metabolites of CBZ, namely CBZE and arene oxides, are able to form covalent adducts with human serum albumin.^5^We hypothesise that reduced clearance of CBZE in subjects with the *EPHX1‐416G/G* genotype leads to greater formation of covalent protein adducts, ultimately resulting in an increased frequency of immune‐mediated hypersensitivity reactions, especially in those expressing the *HLA* risk allele.

Limitations of the current study include the relatively small number of subjects and the limited number of elderly patients. Consequently, the power of the study might have been insufficient to detect significant covariates with small effects or covariates affecting extremes of age only. In addition, several P450 isoforms known to be significant contributors to CBZ hydroxylation, such as CYP1A2,^4^ were not investigated as part of the present analyses. The *CYP1A2* (c.‐163C > A) genotype has been shown to affect CBZ's PK in children.^70^ Finally, the recent discovery of 856 SNPs in human *CYP3A4*,^77^ which codes the principal monoxygenase catalysing CBZ 10,11‐epoxidation,^76^ indicates PK might be influenced by many more genetic polymorphisms than were included in this or any other study. To make significant progress, future studies should attempt to characterise the genetic polymorphisms on a much larger scale in patients with CBZ hypersensitivity reactions in order to determine the influence of many more variants on metabolism pathways within a relevant, pathological, context.

In conclusion, a population PK model has been developed for CBZ which successfully incorporates certain clinical parameters of adult epilepsy patients. We identified carriage of the *EPHX1‐416G/G* genotype as being a significant covariate of CBZE clearance and concomitant treatment with phenytoin and CBZ dose as significant covariates in affecting the PK of CBZ.

## COMPETING INTERESTS

M.P. receives research funding from various organisations including the MRC, NIHR, EU Commission and Health Education England. He has also received partnership funding for the following: MRC Clinical Pharmacology Training Scheme (cofunded by MRC and Roche, UCB, Eli Lilly and Novartis); a PhD studentship jointly funded by EPSRC and Astra Zeneca; and grant funding from VistaGen Therapeutics. He has also unrestricted educational grant support for the UK Pharmacogenetics and Stratified Medicine Network from Bristol‐Myers Squibb and UCB. He has developed an HLA genotyping panel with MC Diagnostics, but does not benefit financially from this. None of the funding declared above has been used for the current paper.

## CONTRIBUTORS

*Study concept and design*: V.L.M.Y., B.K.P., A.G.M. and M.P. *Acquisition of Data*: V.L.M.Y., X.M., J.L.M., D.F.C. and A.G.M. *Model Development*: V.L.M.Y. and H.P. *Analysis or interpretation of Data*: All authors. *Drafting of the manuscript*: V.L.M.Y., H.P. and J.L.M. *Critical revision of the manuscript for important intellectual content*: All authors.

## Supporting information


**Table S1** Inclusion and exclusion criteria for PICME I and PICME II clinical studies
**Table S2** Objective function changes for selected covariate runs
**Figure S1** VPC for the final PK model for each analyte in PICME I

**Figure S2** VPC for the final PK model for each analyte in PICME II (autoinduction group)

**Figure S3** VPC for the final PK model for each analyte in PICME II (maintenance group)

**Figure S4** Raw concentration data for all analytes according to study group

**Figure S5** VPC for the final PK model for each analyte in all study groups

**Data S1** Closed form analytical solution for differential equations of structural model
**Data S2** Coefficients of exponential terms
**Data S3** Analytical solutions for structural model states as functions of dose and time

## Data Availability

The data that support the findings of this study are available from the corresponding author upon reasonable request.
