# Supplementary figures and images for: Evaluation of clinical and genetic factors in the population pharmacokinetics of carbamazepine
Source: Br J Clin Pharmacol. 2020 Dec 14;87(6):2572–88. doi: 10.1111/bcp.14667 (PMC8247401; doi:10.1111/bcp.14667)

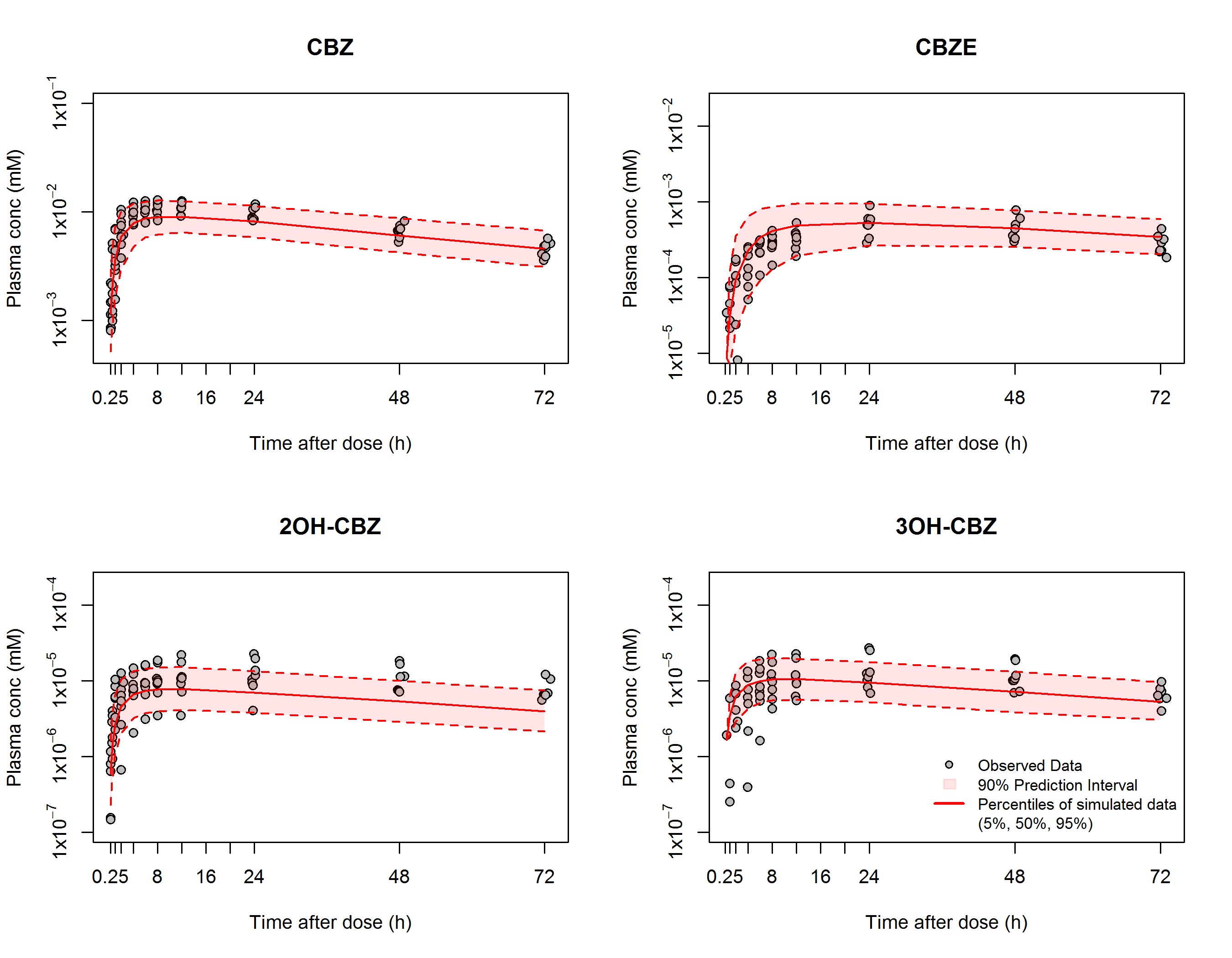

Supplement: Supplementary file 1 — Table S1 Inclusion and exclusion criteria for PICME I and PICME II clinical studies Table S2 Objective function changes for selected covariate runs Figure S1 VPC for the final PK model for each analyte in PICME I [file BCP-87-2572-s004.jpg]

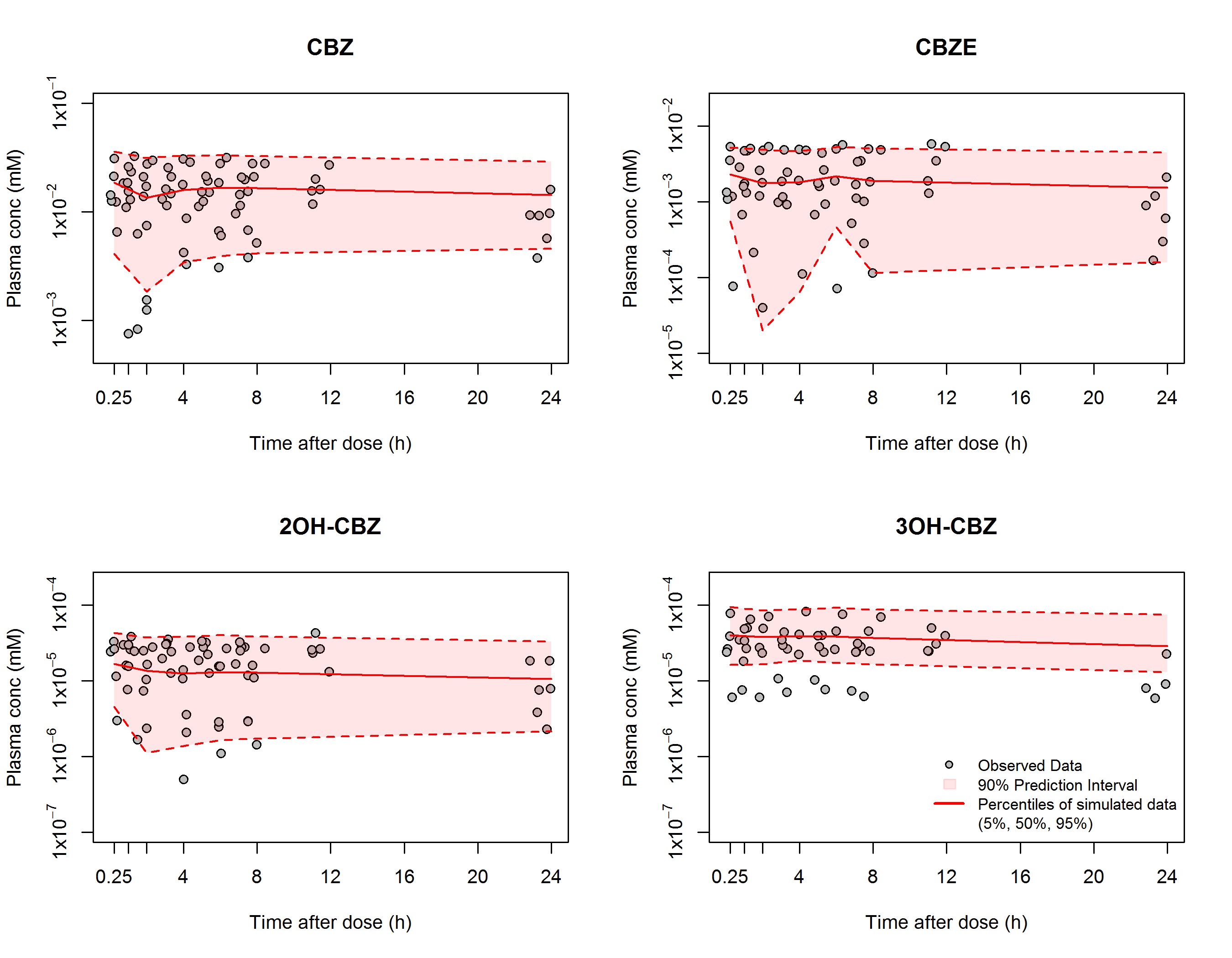

Supplement: Supplementary file 2 — Figure S2 VPC for the final PK model for each analyte in PICME II (autoinduction group) [file BCP-87-2572-s006.jpg]

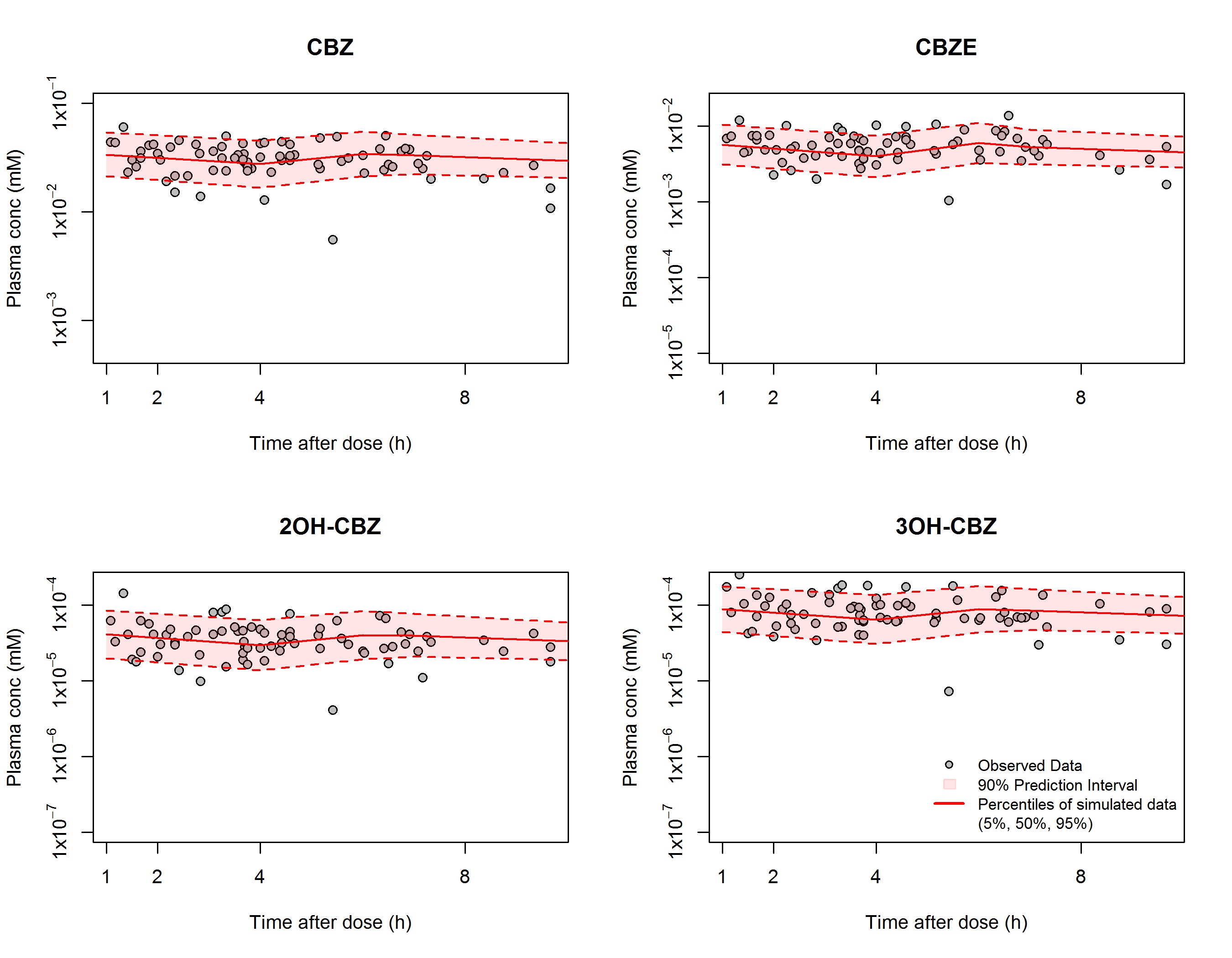

Supplement: Supplementary file 3 — Figure S3 VPC for the final PK model for each analyte in PICME II (maintenance group) [file BCP-87-2572-s003.jpg]

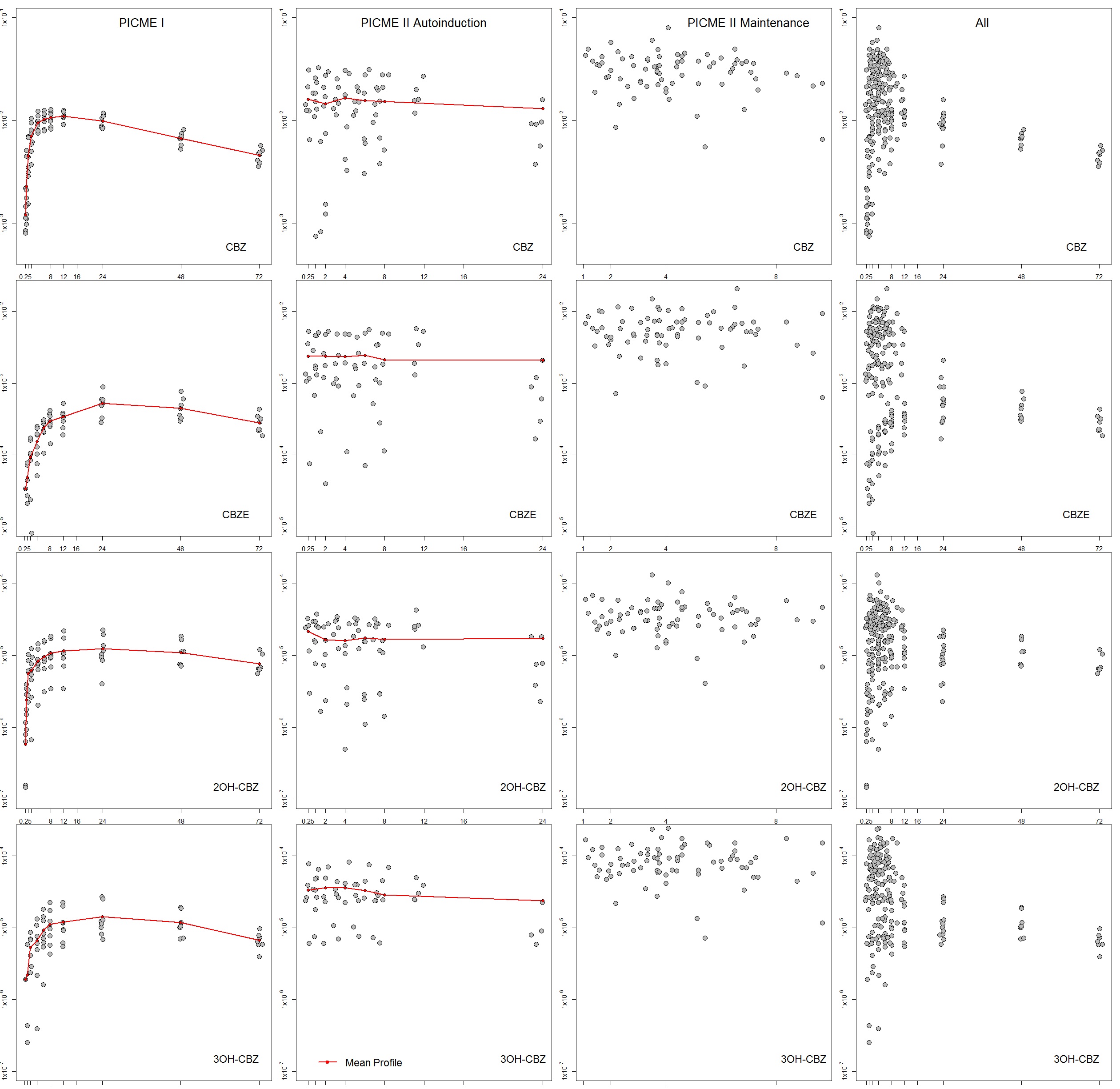

Supplement: Supplementary file 4 — Figure S4 Raw concentration data for all analytes according to study group [file BCP-87-2572-s005.jpg]

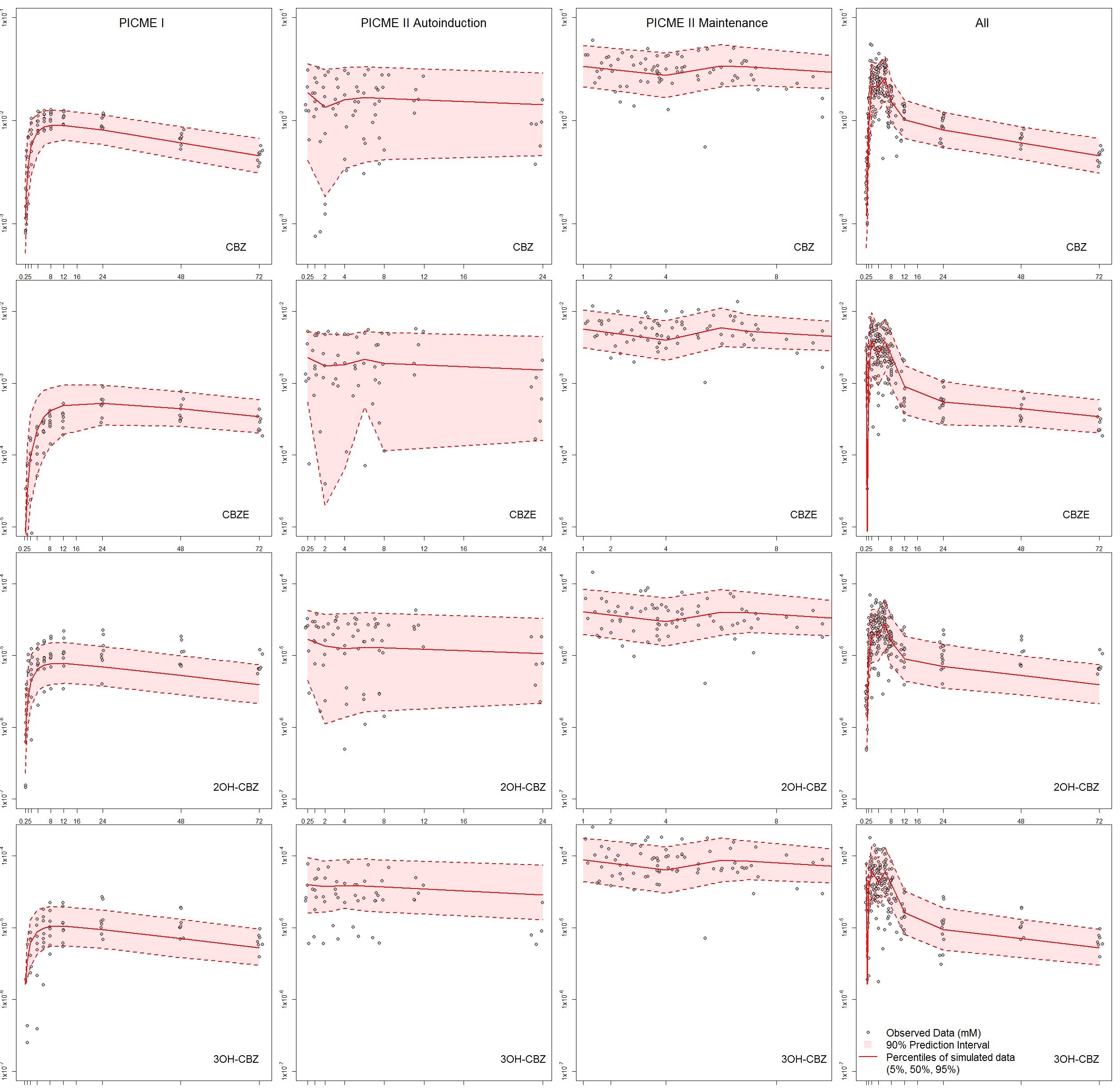

Supplement: Supplementary file 5 — Figure S5 VPC for the final PK model for each analyte in all study groups [file BCP-87-2572-s002.jpg]
