# Supplementary material for: Evaluation of clinical and genetic factors in the population pharmacokinetics of carbamazepine
Source: Br J Clin Pharmacol. 2020 Dec 14;87(6):2572–88. doi: 10.1111/bcp.14667 (PMC8247401; doi:10.1111/bcp.14667)
Supplement: Supplementary file 6 — Data S1 Closed form analytical solution for differential equations of structural model Data S2 Coefficients of exponential terms Data S3 Analytical solutions for structural model states as functions of dose and time [file BCP-87-2572-s001.docx]

**Supplementary Information**

Table 1 - Inclusion and Exclusion Criteria for PICME I and PICME II Clinical Studies

| **Inclusion Criteria** |
| --- |
| - Subject is willing and able to give written informed consent - Healthy male subjects between 18 and 55 years of age inclusive - Subject’s body weight is between 50 and 100 kg - Subject’s body mass index is between 18 and 32 kg/m^2^ |
| **Exclusion Criteria** |
| - Subject is not willing to take part or unable to give written informed consent - Subject has clinically significant abnormal medical history or physical exam - Subject has history of febrile illness within 4 weeks prior to admission - Subject has clinically significant abnormal laboratory test at screening including HBV/HCV/HIV - Subject has taken any interacting prescription or non-prescription drug, or dietary supplements within 2 weeks prior to study admission. Herbal supplements must be discontinued at least 4 weeks prior to admission to the clinical research facility - Subject possesses either the HLA-B*1502 or HLA-A*3101 genotype - Subject has a clinically significant ECG abnormality – prolonged corrected QT>450ms, 2^nd^ or 3^rd^ degree atrioventricular conduction block - Subject has known hypersensitivity to carbamazepine or structurally related drugs (e.g. tricyclic antidepressants) or any other component of the formulation - Subject with history of bone marrow depression - Subject with history of hepatic porphyrias (e.g. intermittent porphyria, variegate porphyria, porphyria cutanea tarda) - Subject has taken part on another research study within 90 days of commencement - Subject has any condition which in the opinion of the investigator will interfere with the study |

Table 2 Objective function changes for all covariate runs

| 1st Round | | |
| --- | --- | --- |
| Model | OFV | DOFV |
| BASE | -14739.0 |  |
| DGRP on CL | -14804.3 | -65.3 |
| AGE on CL | -14738.9 | 0.1 |
| GEN on CL | -14739.3 | -0.3 |
| WT on CL (est. allom. exp) | -14738.7 | 0.3 |
| WT on CL (allom exp fix 0.75) | -14713.4 | 25.6 |
| SMK on CL | -14740.1 | -1.1 |
| VAL on CL | -14739.6 | -0.6 |
| PHT on CL | -14744.3 | -5.3 |
| PHB on CL | -14739.2 | -0.2 |
| FEL on CL | -14739.0 | 0.0 |
| CLOB on CL | -14746.3 | -7.3 |
| LTG on CL | -14739.1 | -0.1 |
| LEV on CL | -14743.8 | -4.8 |
| ZON on CL | -14739.6 | -0.6 |
| TOP on CL | -14743.1 | -4.1 |
| LAC on CL | -14739.9 | -0.9 |
| PRE on CL | -14739.1 | -0.1 |
| PERA on CL | -14739.0 | 0.0 |
| OMEP on CL | -14739.3 | -0.3 |
| STATIN on CL | -14740.5 | -1.5 |
| ABCB1CT N.B. from here down; genotypes = 2 DOF. | -14740.6 | -1.6 |
| ABCB1TC | -14739.6 | -0.6 |
| ABCB1GT | -14739.2 | -0.3 |
| ABCC2GA | -14739.8 | -0.8 |
| ABCC2CT | -14741.6 | -2.6 |
| ABCC2CT2 | -14739.9 | -0.9 |
| UGTAG | -14739.4 | -0.4 |
| UGTAG2 | -14740.6 | -1.6 |
| UGTGA | -14748.7 | -9.7 |
| EPHXTC on CLtot | -14742.2 | -3.2 |
| EPHXTC on FM1 | -14739.1 | -0.1 |
| EPHXTC on CL2E | -14739.2 | -0.2 |
| EPHXAG on CLtot | -14742.0 | -3.0 |
| EPHXAG on FM1 | -14742.2 | -3.2 |
| EPHXAG on CL2E | -14747.7 | -8.8 |
| EPHXAGHOM on CL2E (1 DOF) | -14747.7 | -8.7 |
| 2C83 | -14742.6 | -3.6 |
| 2C84 | -14740.3 | -1.3 |
| 2C192 | -14739.9 | -0.9 |
| 2B6 | -14739.1 | -0.1 |
| 3A4 | -14740.1 | -1.1 |
| 3A5 | -14741.8 | -2.8 |
| MPO | -14745.5 | -6.5 |
| POR | -14740.1 | -1.1 |

| Selected 2nd and 3rd Round | | |
| --- | --- | --- |
| Model | OFV | DOFV |
| Base + DGRP | -14804.3 |  |
| Base + DGRP + PHT | -14810.2 | -5.9 |
| Base + DGRP + CLOB | -14808.1 | -3.8 |
| Base + DGRP + LEV | -14807.5 | -3.1 |
| Base + DGRP + TOP | -14807.5 | -3.2 |
| Base + DGRP + UGTGA (2xD.O.F) | -14805.7 | -1.4 |
| Base + DGRP + EPHXAG (1xD.O.F) | -14813.0 | -8.6 |
| Base + DGRP + EPHXAG (2xD.O.F) | -14813.1 | -8.8 |
|  |  |  |
| Base + DGRP + EPHXAG (1xD.O.F) | -14813.0 |  |
| Base + DGRP + EPHXAG (1xD.O.F) + PHT | -14819.2 | -6.2 |
| Base + DGRP + EPHXAG (1xD.O.F) + CLOB | -14816.8 | -3.8 |
| Base + DGRP + EPHXAG (1xD.O.F) + LEV | -14816.1 | -3.1 |
| Base + DGRP + EPHXAG (1xD.O.F) + TOP | -14816.1 | -3.2 |
| Base + DGRP + EPHXAG (1xD.O.F) + UGTGA (2XD.O.F) | -14814.3 | -1.4 |

**Supplementary Figures**

Supplementary Fig. 1

Prediction corrected visual predictive check for the final pharmacokinetic model fitting for each of the analytes in PICME I (healthy volunteer group). 90% prediction interval (broken line) and median population prediction (continuous line) determined from 1000 simulations for CBZ with the covariate values of those individuals used in the model building process. CBZ – carbamazepine; CBZE – carbamazepine-10,11-epoxide; 2OH-CBZ – 2-hydroxy-carbamazepine; 3OH-CBZ - 3-hydroxy-carbamazepine

Supplementary Fig. 2

Prediction corrected visual predictive check for the final pharmacokinetic model fitting for each of the analytes in PICME II (autoinduction group). 90% prediction interval (broken line) and median population prediction (continuous line) determined from 1000 simulations for CBZ with the covariate values of those individuals used in the model building process. CBZ – carbamazepine; CBZE – carbamazepine-10,11-epoxide; 2OH-CBZ – 2-hydroxy-carbamazepine; 3OH-CBZ - 3-hydroxy-carbamazepine

Supplementary Fig. 3

Prediction corrected visual predictive check for the final pharmacokinetic model fitting for each of the analytes in PICME II (maintenance group). 90% prediction interval (broken line) and median population prediction (continuous line) determined from 1000 simulations for CBZ with the covariate values of those individuals used in the model building process. CBZ – carbamazepine; CBZE – carbamazepine-10,11-epoxide; 2OH-CBZ – 2-hydroxy-carbamazepine; 3OH-CBZ - 3-hydroxy-carbamazepine

Supplementary Fig. 4

Raw concentration data for CBZ and other analytes according to each study group. The red line represents the mean concentration. CBZ – carbamazepine; CBZE – carbamazepine-10,11-epoxide; 2OH-CBZ – 2-hydroxy-carbamazepine; 3OH-CBZ - 3-hydroxy-carbamazepine

Supplementary Fig. 5

Prediction corrected visual predictive check for the final pharmacokinetic model fitting for each of the analytes in each study group. 90% prediction interval (broken line) and median population prediction (continuous line) determined from 1000 simulations for CBZ with the covariate values of those individuals used in the model building process. CBZ – carbamazepine; CBZE – carbamazepine-10,11-epoxide; 2OH-CBZ – 2-hydroxy-carbamazepine; 3OH-CBZ - 3-hydroxy-carbamazepine

**Data S1 Closed form analytical solution for differential equations of structural model**

**Reparameterisations:**

KEL=CL_tot_/V1 KA=KEL+KA_star

KEL2=(1-FM_1_)*KEL KEL2E=CL_2E_/V2

KEL3=FM_1_/2*KEL KEL3E=CL_3E_/V2

KEL4=FM_1_/2*KEL KEL4E=CL_4E_/V2

**Data S2** **Coefficients of exponential terms:**

A=1/((KEL-KA)*(KEL2E-KA)) B=1/((KA-KEL)*(KEL2E-KEL)) C=1/((KA-KEL2E)*(KEL-KEL2E))

D=1/((KEL-KA)*(KEL3E-KA)) E=1/((KA-KEL)*(KEL3E-KEL)) F=1/((KA-KEL3E)*(KEL-KEL3E))

G=1/((KEL-KA)*(KEL4E-KA)) H=1/((KA-KEL)*(KEL4E-KEL)) I=1/((KA-KEL4E)*(KEL-KEL4E))

**Data S3 Analytical solutions for structural model states as functions of dose and time (t):**

A_DEPOT_=dose*exp(-KA*t)

C_CBZ_=(dose/V1)*(KA/(KA-KEL))*(exp(-KEL*t)-exp(-KA*t))

C_CBZE_=((dose*KA*KEL2)/V2)*(A*exp(-KA*t)+B*exp(-KEL*t)+C*exp(-KEL2E*t))

C_2OH-CBZ_=((dose*KA*KEL3)/V2)*(D*exp(-KA*t)+E*exp(-KEL*t)+F*exp(-KEL3E*t))

C_3OH-CBZ_ =((dose*KA*KEL4)/V2)*(G*exp(-KA*t)+H*exp(-KEL*t)+I*exp(-KEL4E*t))

**Final Model NONMEM Results File**

;; 1. Based on: CBZ_CMTs_2356_MET_2018-02-23_run009

;; 2. Description: PK model

$SIZES PD=100 ;Allow 100 columns in datafile

;NO=300

;DIMTMP=10000

;DIMCNS=10000

;DIMNEW=20000

;DIMQ=1000

;DIMVRB=1000

$PROBLEM PK of Carbamazepine and metabolites

$ABBR DERIV2=NO

$ABBR DECLARE DOSETIME(169)

$ABBR DECLARE DOWHILE ILOOP

$ABBR DECLARE DOWHILE NDOSE

$INPUT ID TAD TIME CMT DV AMT1 OFF1 AMT2 OFF2 AMT3 OFF3 AMT4 OFF4

AMT5 OFF5 AMT6 OFF6 AMT7 OFF7 AMT8 OFF8 AMT9 OFF9 AMT10 OFF10 AMT11

OFF11 AMT12 OFF12 AGE GEN WT HT DGRP GRP IND SMK VAL PHT

PHB FEL CLOB LTG LEV ZON TOP LAC PRE PERA OMEP STATIN ABCB1CT_HOM

ABCB1CT_HET ABCB1TC_HOM ABCB1TC_HET ABCB1GT_HOM ABCB1GT_HET ABCC2GA_HOM ABCC2GA_HET

ABCC2CT_HOM ABCC2CT_HET ABCC2CT2_HOM ABCC2CT2_HET UGTAG_HOM UGTAG_HET UGTAG2_HOM

UGTAG2_HET UGTGA_HOM UGTGA_HET EPHXTC_HOM EPHXTC_HET EPHXAG_HOM EPHXAG_HET

HOM_2C83 HET_2C83 HOM_2C84 HET_2C84 HOM_2C192 HET_2C192 HOM_2B6

HET_2B6 HOM_3A4 HET_3A4 HOM_3A5 HET_3A5 MPO_HOM MPO_HET POR_HOM POR_HET

$EST METHOD=1 INTER MAXEVAL=99999 SIGDIG=3 PRINT=4 NOABORT POSTHOC

$COV UNCONDITIONAL

;$SIM (12345) ONLYSIM

$TABLE ID TAD TIME DV Y PRED IPRED CMT CLtot FM1 CL2E CL3E CL4E V1 V2 KA RES WRES CWRES AA BB CC DD EE FF GG HH JJ

NOAPPEND NOPRINT ONEHEADER FILE=CBZ_CMT_2356_run010_res1.tab

$TABLE ID TAD TIME DV PRED IPRED CMT CLtot FM1 CL2E CL3E CL4E V1 V2 KA DGRP GRP

NOAPPEND NOPRINT ONEHEADER FILE=CBZ_CMT_2356_run010_res2.tab

$TABLE ID TAD CLtot FM1 CL2E CL3E CL4E V1 V2 KA AGE GEN WT HT DGRP GRP IND

NOPRINT NOAPPEND FIRSTONLY FILE=CBZ_CMT_2356_run010_param1.tab

$DATA PICME_I_II_III_CMT_2356_mM_full_covariates_2018_02_07_no_zero.csv IGNORE=#

$PRED

; parent CBZ

TVCLtot=THETA(1)*(THETA(9)**IND)*(THETA(17)**PHT)+LOG((DGRP/400)**THETA(15))

CLtot=TVCLtot*EXP(ETA(1))

TVV1=THETA(2)

V1=TVV1*EXP(ETA(2))

TVKA_star=THETA(3)

KA_star=TVKA_star*EXP(ETA(3))

;metabolite formation

TVFM1=THETA(4)

FM1=TVFM1*EXP(ETA(4))

;metabolite elimination clearances

TVCL2E=THETA(5)*(THETA(16)**EPHXAG_HOM)

CL2E=TVCL2E*EXP(ETA(5))

TVCL3E=THETA(6)*(THETA(10)**IND)

CL3E=TVCL3E*EXP(ETA(6))

TVCL4E=THETA(7)

CL4E=TVCL4E*EXP(ETA(7))

; metabolite volume

TVV2=THETA(8)

V2=TVV2*EXP(ETA(8))

;reparameterisation

KEL=CLtot/V1

KA=KEL+KA_star ; fix KA to be faster than KEL

KEL2=(1-FM1)*KEL ;

KEL2E=CL2E/V2 ; loss of CBZE

KEL3=FM1/2*KEL ;PRODUCTION OF 2OH

KEL4=FM1/2*KEL ;PRODUCTION OF 3OH

KEL3E=CL3E/V2 ; assume same. V2 as physchem is similar

KEL4E=CL4E/V2 ;

;scaling

S2=V1 ; CBZ parent

S3=V2 ; CBZE

S5=V2 ; CBZ-2-OH

S6=V2 ; CBZ-3-OH

AA=1/((KEL-KA)*(KEL2E-KA))

BB=1/((KA-KEL)*(KEL2E-KEL))

CC=1/((KA-KEL2E)*(KEL-KEL2E))

DD=1/((KEL-KA)*(KEL3E-KA))

EE=1/((KA-KEL)*(KEL3E-KEL))

FF=1/((KA-KEL3E)*(KEL-KEL3E))

GG=1/((KEL-KA)*(KEL4E-KA))

HH=1/((KA-KEL)*(KEL4E-KEL))

JJ=1/((KA-KEL4E)*(KEL-KEL4E))

C2_B1=0

C3_B1=0

C5_B1=0

C6_B1=0

DOSETIME(1)=TIME-((1-1)*12+OFF1)

IF(DOSETIME(1).LT.0)DOSETIME(1)=0

DOSETIME(2)=TIME-((2-1)*12+OFF2)

IF(DOSETIME(2).LT.0)DOSETIME(2)=0

DOSETIME(3)=TIME-((3-1)*12+OFF1)

IF(DOSETIME(3).LT.0)DOSETIME(3)=0

DOSETIME(4)=TIME-((4-1)*12+OFF2)

IF(DOSETIME(4).LT.0)DOSETIME(4)=0

DOSETIME(5)=TIME-((5-1)*12+OFF1)

IF(DOSETIME(5).LT.0)DOSETIME(5)=0

DOSETIME(6)=TIME-((6-1)*12+OFF2)

IF(DOSETIME(6).LT.0)DOSETIME(6)=0

DOSETIME(7)=TIME-((7-1)*12+OFF1)

IF(DOSETIME(7).LT.0)DOSETIME(7)=0

DOSETIME(8)=TIME-((8-1)*12+OFF2)

IF(DOSETIME(8).LT.0)DOSETIME(8)=0

DOSETIME(9)=TIME-((9-1)*12+OFF1)

IF(DOSETIME(9).LT.0)DOSETIME(9)=0

DOSETIME(10)=TIME-((10-1)*12+OFF2)

IF(DOSETIME(10).LT.0)DOSETIME(10)=0

DOSETIME(11)=TIME-((11-1)*12+OFF1)

IF(DOSETIME(11).LT.0)DOSETIME(11)=0

DOSETIME(12)=TIME-((12-1)*12+OFF2)

IF(DOSETIME(12).LT.0)DOSETIME(12)=0

DOSETIME(13)=TIME-((13-1)*12+OFF1)

IF(DOSETIME(13).LT.0)DOSETIME(13)=0

DOSETIME(14)=TIME-((14-1)*12+OFF2)

IF(DOSETIME(14).LT.0)DOSETIME(14)=0

DOSETIME(15)=TIME-((15-1)*12+OFF1)

IF(DOSETIME(15).LT.0)DOSETIME(15)=0

DOSETIME(16)=TIME-((16-1)*12+OFF2)

IF(DOSETIME(16).LT.0)DOSETIME(16)=0

DOSETIME(17)=TIME-((17-1)*12+OFF1)

IF(DOSETIME(17).LT.0)DOSETIME(17)=0

DOSETIME(18)=TIME-((18-1)*12+OFF2)

IF(DOSETIME(18).LT.0)DOSETIME(18)=0

DOSETIME(19)=TIME-((19-1)*12+OFF1)

IF(DOSETIME(19).LT.0)DOSETIME(19)=0

DOSETIME(20)=TIME-((20-1)*12+OFF2)

IF(DOSETIME(20).LT.0)DOSETIME(20)=0

DOSETIME(21)=TIME-((21-1)*12+OFF1)

IF(DOSETIME(21).LT.0)DOSETIME(21)=0

DOSETIME(22)=TIME-((22-1)*12+OFF2)

IF(DOSETIME(22).LT.0)DOSETIME(22)=0

DOSETIME(23)=TIME-((23-1)*12+OFF1)

IF(DOSETIME(23).LT.0)DOSETIME(23)=0

DOSETIME(24)=TIME-((24-1)*12+OFF2)

IF(DOSETIME(24).LT.0)DOSETIME(24)=0

DOSETIME(25)=TIME-((25-1)*12+OFF1)

IF(DOSETIME(25).LT.0)DOSETIME(25)=0

DOSETIME(26)=TIME-((26-1)*12+OFF2)

IF(DOSETIME(26).LT.0)DOSETIME(26)=0

DOSETIME(27)=TIME-((27-1)*12+OFF1)

IF(DOSETIME(27).LT.0)DOSETIME(27)=0

DOSETIME(28)=TIME-((28-1)*12+OFF2)

IF(DOSETIME(28).LT.0)DOSETIME(28)=0

DOSETIME(29)=TIME-((29-1)*12+OFF3)

IF(DOSETIME(29).LT.0)DOSETIME(29)=0

DOSETIME(30)=TIME-((30-1)*12+OFF4)

IF(DOSETIME(30).LT.0)DOSETIME(30)=0

DOSETIME(31)=TIME-((31-1)*12+OFF3)

IF(DOSETIME(31).LT.0)DOSETIME(31)=0

DOSETIME(32)=TIME-((32-1)*12+OFF4)

IF(DOSETIME(32).LT.0)DOSETIME(32)=0

DOSETIME(33)=TIME-((33-1)*12+OFF3)

IF(DOSETIME(33).LT.0)DOSETIME(33)=0

DOSETIME(34)=TIME-((34-1)*12+OFF4)

IF(DOSETIME(34).LT.0)DOSETIME(34)=0

DOSETIME(35)=TIME-((35-1)*12+OFF3)

IF(DOSETIME(35).LT.0)DOSETIME(35)=0

DOSETIME(36)=TIME-((36-1)*12+OFF4)

IF(DOSETIME(36).LT.0)DOSETIME(36)=0

DOSETIME(37)=TIME-((37-1)*12+OFF3)

IF(DOSETIME(37).LT.0)DOSETIME(37)=0

DOSETIME(38)=TIME-((38-1)*12+OFF4)

IF(DOSETIME(38).LT.0)DOSETIME(38)=0

DOSETIME(39)=TIME-((39-1)*12+OFF3)

IF(DOSETIME(39).LT.0)DOSETIME(39)=0

DOSETIME(40)=TIME-((40-1)*12+OFF4)

IF(DOSETIME(40).LT.0)DOSETIME(40)=0

DOSETIME(41)=TIME-((41-1)*12+OFF3)

IF(DOSETIME(41).LT.0)DOSETIME(41)=0

DOSETIME(42)=TIME-((42-1)*12+OFF4)

IF(DOSETIME(42).LT.0)DOSETIME(42)=0

DOSETIME(43)=TIME-((43-1)*12+OFF3)

IF(DOSETIME(43).LT.0)DOSETIME(43)=0

DOSETIME(44)=TIME-((44-1)*12+OFF4)

IF(DOSETIME(44).LT.0)DOSETIME(44)=0

DOSETIME(45)=TIME-((45-1)*12+OFF3)

IF(DOSETIME(45).LT.0)DOSETIME(45)=0

DOSETIME(46)=TIME-((46-1)*12+OFF4)

IF(DOSETIME(46).LT.0)DOSETIME(46)=0

DOSETIME(47)=TIME-((47-1)*12+OFF3)

IF(DOSETIME(47).LT.0)DOSETIME(47)=0

DOSETIME(48)=TIME-((48-1)*12+OFF4)

IF(DOSETIME(48).LT.0)DOSETIME(48)=0

DOSETIME(49)=TIME-((49-1)*12+OFF3)

IF(DOSETIME(49).LT.0)DOSETIME(49)=0

DOSETIME(50)=TIME-((50-1)*12+OFF4)

IF(DOSETIME(50).LT.0)DOSETIME(50)=0

DOSETIME(51)=TIME-((51-1)*12+OFF3)

IF(DOSETIME(51).LT.0)DOSETIME(51)=0

DOSETIME(52)=TIME-((52-1)*12+OFF4)

IF(DOSETIME(52).LT.0)DOSETIME(52)=0

DOSETIME(53)=TIME-((53-1)*12+OFF3)

IF(DOSETIME(53).LT.0)DOSETIME(53)=0

DOSETIME(54)=TIME-((54-1)*12+OFF4)

IF(DOSETIME(54).LT.0)DOSETIME(54)=0

DOSETIME(55)=TIME-((55-1)*12+OFF3)

IF(DOSETIME(55).LT.0)DOSETIME(55)=0

DOSETIME(56)=TIME-((56-1)*12+OFF4)

IF(DOSETIME(56).LT.0)DOSETIME(56)=0

DOSETIME(57)=TIME-((57-1)*12+OFF5)

IF(DOSETIME(57).LT.0)DOSETIME(57)=0

DOSETIME(58)=TIME-((58-1)*12+OFF6)

IF(DOSETIME(58).LT.0)DOSETIME(58)=0

DOSETIME(59)=TIME-((59-1)*12+OFF5)

IF(DOSETIME(59).LT.0)DOSETIME(59)=0

DOSETIME(60)=TIME-((60-1)*12+OFF6)

IF(DOSETIME(60).LT.0)DOSETIME(60)=0

DOSETIME(61)=TIME-((61-1)*12+OFF5)

IF(DOSETIME(61).LT.0)DOSETIME(61)=0

DOSETIME(62)=TIME-((62-1)*12+OFF6)

IF(DOSETIME(62).LT.0)DOSETIME(62)=0

DOSETIME(63)=TIME-((63-1)*12+OFF5)

IF(DOSETIME(63).LT.0)DOSETIME(63)=0

DOSETIME(64)=TIME-((64-1)*12+OFF6)

IF(DOSETIME(64).LT.0)DOSETIME(64)=0

DOSETIME(65)=TIME-((65-1)*12+OFF5)

IF(DOSETIME(65).LT.0)DOSETIME(65)=0

DOSETIME(66)=TIME-((66-1)*12+OFF6)

IF(DOSETIME(66).LT.0)DOSETIME(66)=0

DOSETIME(67)=TIME-((67-1)*12+OFF5)

IF(DOSETIME(67).LT.0)DOSETIME(67)=0

DOSETIME(68)=TIME-((68-1)*12+OFF6)

IF(DOSETIME(68).LT.0)DOSETIME(68)=0

DOSETIME(69)=TIME-((69-1)*12+OFF5)

IF(DOSETIME(69).LT.0)DOSETIME(69)=0

DOSETIME(70)=TIME-((70-1)*12+OFF6)

IF(DOSETIME(70).LT.0)DOSETIME(70)=0

DOSETIME(71)=TIME-((71-1)*12+OFF5)

IF(DOSETIME(71).LT.0)DOSETIME(71)=0

DOSETIME(72)=TIME-((72-1)*12+OFF6)

IF(DOSETIME(72).LT.0)DOSETIME(72)=0

DOSETIME(73)=TIME-((73-1)*12+OFF5)

IF(DOSETIME(73).LT.0)DOSETIME(73)=0

DOSETIME(74)=TIME-((74-1)*12+OFF6)

IF(DOSETIME(74).LT.0)DOSETIME(74)=0

DOSETIME(75)=TIME-((75-1)*12+OFF5)

IF(DOSETIME(75).LT.0)DOSETIME(75)=0

DOSETIME(76)=TIME-((76-1)*12+OFF6)

IF(DOSETIME(76).LT.0)DOSETIME(76)=0

DOSETIME(77)=TIME-((77-1)*12+OFF5)

IF(DOSETIME(77).LT.0)DOSETIME(77)=0

DOSETIME(78)=TIME-((78-1)*12+OFF6)

IF(DOSETIME(78).LT.0)DOSETIME(78)=0

DOSETIME(79)=TIME-((79-1)*12+OFF5)

IF(DOSETIME(79).LT.0)DOSETIME(79)=0

DOSETIME(80)=TIME-((80-1)*12+OFF6)

IF(DOSETIME(80).LT.0)DOSETIME(80)=0

DOSETIME(81)=TIME-((81-1)*12+OFF5)

IF(DOSETIME(81).LT.0)DOSETIME(81)=0

DOSETIME(82)=TIME-((82-1)*12+OFF6)

IF(DOSETIME(82).LT.0)DOSETIME(82)=0

DOSETIME(83)=TIME-((83-1)*12+OFF5)

IF(DOSETIME(83).LT.0)DOSETIME(83)=0

DOSETIME(84)=TIME-((84-1)*12+OFF6)

IF(DOSETIME(84).LT.0)DOSETIME(84)=0

DOSETIME(85)=TIME-((85-1)*12+OFF7)

IF(DOSETIME(85).LT.0)DOSETIME(85)=0

DOSETIME(86)=TIME-((86-1)*12+OFF8)

IF(DOSETIME(86).LT.0)DOSETIME(86)=0

DOSETIME(87)=TIME-((87-1)*12+OFF7)

IF(DOSETIME(87).LT.0)DOSETIME(87)=0

DOSETIME(88)=TIME-((88-1)*12+OFF8)

IF(DOSETIME(88).LT.0)DOSETIME(88)=0

DOSETIME(89)=TIME-((89-1)*12+OFF7)

IF(DOSETIME(89).LT.0)DOSETIME(89)=0

DOSETIME(90)=TIME-((90-1)*12+OFF8)

IF(DOSETIME(90).LT.0)DOSETIME(90)=0

DOSETIME(91)=TIME-((91-1)*12+OFF7)

IF(DOSETIME(91).LT.0)DOSETIME(91)=0

DOSETIME(92)=TIME-((92-1)*12+OFF8)

IF(DOSETIME(92).LT.0)DOSETIME(92)=0

DOSETIME(93)=TIME-((93-1)*12+OFF7)

IF(DOSETIME(93).LT.0)DOSETIME(93)=0

DOSETIME(94)=TIME-((94-1)*12+OFF8)

IF(DOSETIME(94).LT.0)DOSETIME(94)=0

DOSETIME(95)=TIME-((95-1)*12+OFF7)

IF(DOSETIME(95).LT.0)DOSETIME(95)=0

DOSETIME(96)=TIME-((96-1)*12+OFF8)

IF(DOSETIME(96).LT.0)DOSETIME(96)=0

DOSETIME(97)=TIME-((97-1)*12+OFF7)

IF(DOSETIME(97).LT.0)DOSETIME(97)=0

DOSETIME(98)=TIME-((98-1)*12+OFF8)

IF(DOSETIME(98).LT.0)DOSETIME(98)=0

DOSETIME(99)=TIME-((99-1)*12+OFF7)

IF(DOSETIME(99).LT.0)DOSETIME(99)=0

DOSETIME(100)=TIME-((100-1)*12+OFF8)

IF(DOSETIME(100).LT.0)DOSETIME(100)=0

DOSETIME(101)=TIME-((101-1)*12+OFF7)

IF(DOSETIME(101).LT.0)DOSETIME(101)=0

DOSETIME(102)=TIME-((102-1)*12+OFF8)

IF(DOSETIME(102).LT.0)DOSETIME(102)=0

DOSETIME(103)=TIME-((103-1)*12+OFF7)

IF(DOSETIME(103).LT.0)DOSETIME(103)=0

DOSETIME(104)=TIME-((104-1)*12+OFF8)

IF(DOSETIME(104).LT.0)DOSETIME(104)=0

DOSETIME(105)=TIME-((105-1)*12+OFF7)

IF(DOSETIME(105).LT.0)DOSETIME(105)=0

DOSETIME(106)=TIME-((106-1)*12+OFF8)

IF(DOSETIME(106).LT.0)DOSETIME(106)=0

DOSETIME(107)=TIME-((107-1)*12+OFF7)

IF(DOSETIME(107).LT.0)DOSETIME(107)=0

DOSETIME(108)=TIME-((108-1)*12+OFF8)

IF(DOSETIME(108).LT.0)DOSETIME(108)=0

DOSETIME(109)=TIME-((109-1)*12+OFF7)

IF(DOSETIME(109).LT.0)DOSETIME(109)=0

DOSETIME(110)=TIME-((110-1)*12+OFF8)

IF(DOSETIME(110).LT.0)DOSETIME(110)=0

DOSETIME(111)=TIME-((111-1)*12+OFF7)

IF(DOSETIME(111).LT.0)DOSETIME(111)=0

DOSETIME(112)=TIME-((112-1)*12+OFF8)

IF(DOSETIME(112).LT.0)DOSETIME(112)=0

DOSETIME(113)=TIME-((113-1)*12+OFF9)

IF(DOSETIME(113).LT.0)DOSETIME(113)=0

DOSETIME(114)=TIME-((114-1)*12+OFF10)

IF(DOSETIME(114).LT.0)DOSETIME(114)=0

DOSETIME(115)=TIME-((115-1)*12+OFF9)

IF(DOSETIME(115).LT.0)DOSETIME(115)=0

DOSETIME(116)=TIME-((116-1)*12+OFF10)

IF(DOSETIME(116).LT.0)DOSETIME(116)=0

DOSETIME(117)=TIME-((117-1)*12+OFF9)

IF(DOSETIME(117).LT.0)DOSETIME(117)=0

DOSETIME(118)=TIME-((118-1)*12+OFF10)

IF(DOSETIME(118).LT.0)DOSETIME(118)=0

DOSETIME(119)=TIME-((119-1)*12+OFF9)

IF(DOSETIME(119).LT.0)DOSETIME(119)=0

DOSETIME(120)=TIME-((120-1)*12+OFF10)

IF(DOSETIME(120).LT.0)DOSETIME(120)=0

DOSETIME(121)=TIME-((121-1)*12+OFF9)

IF(DOSETIME(121).LT.0)DOSETIME(121)=0

DOSETIME(122)=TIME-((122-1)*12+OFF10)

IF(DOSETIME(122).LT.0)DOSETIME(122)=0

DOSETIME(123)=TIME-((123-1)*12+OFF9)

IF(DOSETIME(123).LT.0)DOSETIME(123)=0

DOSETIME(124)=TIME-((124-1)*12+OFF10)

IF(DOSETIME(124).LT.0)DOSETIME(124)=0

DOSETIME(125)=TIME-((125-1)*12+OFF9)

IF(DOSETIME(125).LT.0)DOSETIME(125)=0

DOSETIME(126)=TIME-((126-1)*12+OFF10)

IF(DOSETIME(126).LT.0)DOSETIME(126)=0

DOSETIME(127)=TIME-((127-1)*12+OFF9)

IF(DOSETIME(127).LT.0)DOSETIME(127)=0

DOSETIME(128)=TIME-((128-1)*12+OFF10)

IF(DOSETIME(128).LT.0)DOSETIME(128)=0

DOSETIME(129)=TIME-((129-1)*12+OFF9)

IF(DOSETIME(129).LT.0)DOSETIME(129)=0

DOSETIME(130)=TIME-((130-1)*12+OFF10)

IF(DOSETIME(130).LT.0)DOSETIME(130)=0

DOSETIME(131)=TIME-((131-1)*12+OFF9)

IF(DOSETIME(131).LT.0)DOSETIME(131)=0

DOSETIME(132)=TIME-((132-1)*12+OFF10)

IF(DOSETIME(132).LT.0)DOSETIME(132)=0

DOSETIME(133)=TIME-((133-1)*12+OFF9)

IF(DOSETIME(133).LT.0)DOSETIME(133)=0

DOSETIME(134)=TIME-((134-1)*12+OFF10)

IF(DOSETIME(134).LT.0)DOSETIME(134)=0

DOSETIME(135)=TIME-((135-1)*12+OFF9)

IF(DOSETIME(135).LT.0)DOSETIME(135)=0

DOSETIME(136)=TIME-((136-1)*12+OFF10)

IF(DOSETIME(136).LT.0)DOSETIME(136)=0

DOSETIME(137)=TIME-((137-1)*12+OFF9)

IF(DOSETIME(137).LT.0)DOSETIME(137)=0

DOSETIME(138)=TIME-((138-1)*12+OFF10)

IF(DOSETIME(138).LT.0)DOSETIME(138)=0

DOSETIME(139)=TIME-((139-1)*12+OFF9)

IF(DOSETIME(139).LT.0)DOSETIME(139)=0

DOSETIME(140)=TIME-((140-1)*12+OFF10)

IF(DOSETIME(140).LT.0)DOSETIME(140)=0

DOSETIME(141)=TIME-((141-1)*12+OFF11)

IF(DOSETIME(141).LT.0)DOSETIME(141)=0

DOSETIME(142)=TIME-((142-1)*12+OFF12)

IF(DOSETIME(142).LT.0)DOSETIME(142)=0

DOSETIME(143)=TIME-((143-1)*12+OFF11)

IF(DOSETIME(143).LT.0)DOSETIME(143)=0

DOSETIME(144)=TIME-((144-1)*12+OFF12)

IF(DOSETIME(144).LT.0)DOSETIME(144)=0

DOSETIME(145)=TIME-((145-1)*12+OFF11)

IF(DOSETIME(145).LT.0)DOSETIME(145)=0

DOSETIME(146)=TIME-((146-1)*12+OFF12)

IF(DOSETIME(146).LT.0)DOSETIME(146)=0

DOSETIME(147)=TIME-((147-1)*12+OFF11)

IF(DOSETIME(147).LT.0)DOSETIME(147)=0

DOSETIME(148)=TIME-((148-1)*12+OFF12)

IF(DOSETIME(148).LT.0)DOSETIME(148)=0

DOSETIME(149)=TIME-((149-1)*12+OFF11)

IF(DOSETIME(149).LT.0)DOSETIME(149)=0

DOSETIME(150)=TIME-((150-1)*12+OFF12)

IF(DOSETIME(150).LT.0)DOSETIME(150)=0

DOSETIME(151)=TIME-((151-1)*12+OFF11)

IF(DOSETIME(151).LT.0)DOSETIME(151)=0

DOSETIME(152)=TIME-((152-1)*12+OFF12)

IF(DOSETIME(152).LT.0)DOSETIME(152)=0

DOSETIME(153)=TIME-((153-1)*12+OFF11)

IF(DOSETIME(153).LT.0)DOSETIME(153)=0

DOSETIME(154)=TIME-((154-1)*12+OFF12)

IF(DOSETIME(154).LT.0)DOSETIME(154)=0

DOSETIME(155)=TIME-((155-1)*12+OFF11)

IF(DOSETIME(155).LT.0)DOSETIME(155)=0

DOSETIME(156)=TIME-((156-1)*12+OFF12)

IF(DOSETIME(156).LT.0)DOSETIME(156)=0

DOSETIME(157)=TIME-((157-1)*12+OFF11)

IF(DOSETIME(157).LT.0)DOSETIME(157)=0

DOSETIME(158)=TIME-((158-1)*12+OFF12)

IF(DOSETIME(158).LT.0)DOSETIME(158)=0

DOSETIME(159)=TIME-((159-1)*12+OFF11)

IF(DOSETIME(159).LT.0)DOSETIME(159)=0

DOSETIME(160)=TIME-((160-1)*12+OFF12)

IF(DOSETIME(160).LT.0)DOSETIME(160)=0

DOSETIME(161)=TIME-((161-1)*12+OFF11)

IF(DOSETIME(161).LT.0)DOSETIME(161)=0

DOSETIME(162)=TIME-((162-1)*12+OFF12)

IF(DOSETIME(162).LT.0)DOSETIME(162)=0

DOSETIME(163)=TIME-((163-1)*12+OFF11)

IF(DOSETIME(163).LT.0)DOSETIME(163)=0

DOSETIME(164)=TIME-((164-1)*12+OFF12)

IF(DOSETIME(164).LT.0)DOSETIME(164)=0

DOSETIME(165)=TIME-((165-1)*12+OFF11)

IF(DOSETIME(165).LT.0)DOSETIME(165)=0

DOSETIME(166)=TIME-((166-1)*12+OFF12)

IF(DOSETIME(166).LT.0)DOSETIME(166)=0

DOSETIME(167)=TIME-((167-1)*12+OFF11)

IF(DOSETIME(167).LT.0)DOSETIME(167)=0

DOSETIME(168)=TIME-((168-1)*12+OFF12)

IF(DOSETIME(168).LT.0)DOSETIME(168)=0

DOSETIME(169)=TIME-((169-1)*12+OFF11)

IF(DOSETIME(169).LT.0)DOSETIME(169)=0

ILOOP=1

DO WHILE(ILOOP<=14)

C2_B1=C2_B1+(AMT1/V1)*(KA/(KA-KEL))*(EXP(-KEL*DOSETIME(ILOOP*2-1))-EXP(-KA*DOSETIME(ILOOP*2-1))) &

+(AMT2/V1)*(KA/(KA-KEL))*(EXP(-KEL*DOSETIME(ILOOP*2))-EXP(-KA*DOSETIME(ILOOP*2)))

C3_B1=C3_B1+((AMT1*KA*KEL2)/V2)*(AA*EXP(-KA*DOSETIME(ILOOP*2-1))+BB*EXP(-KEL*DOSETIME(ILOOP*2-1))+CC*EXP(-KEL2E*DOSETIME(ILOOP*2-1))) &

+((AMT2*KA*KEL2)/V2)*(AA*EXP(-KA*DOSETIME(ILOOP*2))+BB*EXP(-KEL*DOSETIME(ILOOP*2))+CC*EXP(-KEL2E*DOSETIME(ILOOP*2)))

C5_B1=C5_B1+((AMT1*KA*KEL3)/V2)*(DD*EXP(-KA*DOSETIME(ILOOP*2-1))+EE*EXP(-KEL*DOSETIME(ILOOP*2-1))+FF*EXP(-KEL3E*DOSETIME(ILOOP*2-1))) &

+((AMT2*KA*KEL3)/V2)*(DD*EXP(-KA*DOSETIME(ILOOP*2))+EE*EXP(-KEL*DOSETIME(ILOOP*2))+FF*EXP(-KEL3E*DOSETIME(ILOOP*2)))

C6_B1=C6_B1+((AMT1*KA*KEL4)/V2)*(GG*EXP(-KA*DOSETIME(ILOOP*2-1))+HH*EXP(-KEL*DOSETIME(ILOOP*2-1))+JJ*EXP(-KEL4E*DOSETIME(ILOOP*2-1))) &

+((AMT2*KA*KEL4)/V2)*(GG*EXP(-KA*DOSETIME(ILOOP*2))+HH*EXP(-KEL*DOSETIME(ILOOP*2))+JJ*EXP(-KEL4E*DOSETIME(ILOOP*2)))

ILOOP=ILOOP+1

ENDDO

C2_B2=0

C3_B2=0

C5_B2=0

C6_B2=0

ILOOP=15

DO WHILE(ILOOP<=28)

C2_B2=C2_B2+(AMT3/V1)*(KA/(KA-KEL))*(EXP(-KEL*DOSETIME(ILOOP*2-1))-EXP(-KA*DOSETIME(ILOOP*2-1))) &

+(AMT4/V1)*(KA/(KA-KEL))*(EXP(-KEL*DOSETIME(ILOOP*2))-EXP(-KA*DOSETIME(ILOOP*2)))

C3_B2=C3_B2+((AMT3*KA*KEL2)/V2)*(AA*EXP(-KA*DOSETIME(ILOOP*2-1))+BB*EXP(-KEL*DOSETIME(ILOOP*2-1))+CC*EXP(-KEL2E*DOSETIME(ILOOP*2-1))) &

+((AMT4*KA*KEL2)/V2)*(AA*EXP(-KA*DOSETIME(ILOOP*2))+BB*EXP(-KEL*DOSETIME(ILOOP*2))+CC*EXP(-KEL2E*DOSETIME(ILOOP*2)))

C5_B2=C5_B2+((AMT3*KA*KEL3)/V2)*(DD*EXP(-KA*DOSETIME(ILOOP*2-1))+EE*EXP(-KEL*DOSETIME(ILOOP*2-1))+FF*EXP(-KEL3E*DOSETIME(ILOOP*2-1))) &

+((AMT4*KA*KEL3)/V2)*(DD*EXP(-KA*DOSETIME(ILOOP*2))+EE*EXP(-KEL*DOSETIME(ILOOP*2))+FF*EXP(-KEL3E*DOSETIME(ILOOP*2)))

C6_B2=C6_B2+((AMT3*KA*KEL4)/V2)*(GG*EXP(-KA*DOSETIME(ILOOP*2-1))+HH*EXP(-KEL*DOSETIME(ILOOP*2-1))+JJ*EXP(-KEL4E*DOSETIME(ILOOP*2-1))) &

+((AMT4*KA*KEL4)/V2)*(GG*EXP(-KA*DOSETIME(ILOOP*2))+HH*EXP(-KEL*DOSETIME(ILOOP*2))+JJ*EXP(-KEL4E*DOSETIME(ILOOP*2)))

ILOOP=ILOOP+1

ENDDO

C2_B3=0

C3_B3=0

C5_B3=0

C6_B3=0

ILOOP=29

DO WHILE(ILOOP<=42)

C2_B3=C2_B3+(AMT5/V1)*(KA/(KA-KEL))*(EXP(-KEL*DOSETIME(ILOOP*2-1))-EXP(-KA*DOSETIME(ILOOP*2-1))) &

+(AMT6/V1)*(KA/(KA-KEL))*(EXP(-KEL*DOSETIME(ILOOP*2))-EXP(-KA*DOSETIME(ILOOP*2)))

C3_B3=C3_B3+((AMT5*KA*KEL2)/V2)*(AA*EXP(-KA*DOSETIME(ILOOP*2-1))+BB*EXP(-KEL*DOSETIME(ILOOP*2-1))+CC*EXP(-KEL2E*DOSETIME(ILOOP*2-1))) &

+((AMT6*KA*KEL2)/V2)*(AA*EXP(-KA*DOSETIME(ILOOP*2))+BB*EXP(-KEL*DOSETIME(ILOOP*2))+CC*EXP(-KEL2E*DOSETIME(ILOOP*2)))

C5_B3=C5_B3+((AMT5*KA*KEL3)/V2)*(DD*EXP(-KA*DOSETIME(ILOOP*2-1))+EE*EXP(-KEL*DOSETIME(ILOOP*2-1))+FF*EXP(-KEL3E*DOSETIME(ILOOP*2-1))) &

+((AMT6*KA*KEL3)/V2)*(DD*EXP(-KA*DOSETIME(ILOOP*2))+EE*EXP(-KEL*DOSETIME(ILOOP*2))+FF*EXP(-KEL3E*DOSETIME(ILOOP*2)))

C6_B3=C6_B3+((AMT5*KA*KEL4)/V2)*(GG*EXP(-KA*DOSETIME(ILOOP*2-1))+HH*EXP(-KEL*DOSETIME(ILOOP*2-1))+JJ*EXP(-KEL4E*DOSETIME(ILOOP*2-1))) &

+((AMT6*KA*KEL4)/V2)*(GG*EXP(-KA*DOSETIME(ILOOP*2))+HH*EXP(-KEL*DOSETIME(ILOOP*2))+JJ*EXP(-KEL4E*DOSETIME(ILOOP*2)))

ILOOP=ILOOP+1

ENDDO

C2_B4=0

C3_B4=0

C5_B4=0

C6_B4=0

ILOOP=43

DO WHILE(ILOOP<=56)

C2_B4=C2_B4+(AMT7/V1)*(KA/(KA-KEL))*(EXP(-KEL*DOSETIME(ILOOP*2-1))-EXP(-KA*DOSETIME(ILOOP*2-1))) &

+(AMT8/V1)*(KA/(KA-KEL))*(EXP(-KEL*DOSETIME(ILOOP*2))-EXP(-KA*DOSETIME(ILOOP*2)))

C3_B4=C3_B4+((AMT7*KA*KEL2)/V2)*(AA*EXP(-KA*DOSETIME(ILOOP*2-1))+BB*EXP(-KEL*DOSETIME(ILOOP*2-1))+CC*EXP(-KEL2E*DOSETIME(ILOOP*2-1))) &

+((AMT8*KA*KEL2)/V2)*(AA*EXP(-KA*DOSETIME(ILOOP*2))+BB*EXP(-KEL*DOSETIME(ILOOP*2))+CC*EXP(-KEL2E*DOSETIME(ILOOP*2)))

C5_B4=C5_B4+((AMT7*KA*KEL3)/V2)*(DD*EXP(-KA*DOSETIME(ILOOP*2-1))+EE*EXP(-KEL*DOSETIME(ILOOP*2-1))+FF*EXP(-KEL3E*DOSETIME(ILOOP*2-1))) &

+((AMT8*KA*KEL3)/V2)*(DD*EXP(-KA*DOSETIME(ILOOP*2))+EE*EXP(-KEL*DOSETIME(ILOOP*2))+FF*EXP(-KEL3E*DOSETIME(ILOOP*2)))

C6_B4=C6_B4+((AMT7*KA*KEL4)/V2)*(GG*EXP(-KA*DOSETIME(ILOOP*2-1))+HH*EXP(-KEL*DOSETIME(ILOOP*2-1))+JJ*EXP(-KEL4E*DOSETIME(ILOOP*2-1))) &

+((AMT8*KA*KEL4)/V2)*(GG*EXP(-KA*DOSETIME(ILOOP*2))+HH*EXP(-KEL*DOSETIME(ILOOP*2))+JJ*EXP(-KEL4E*DOSETIME(ILOOP*2)))

ILOOP=ILOOP+1

ENDDO

C2_B5=0

C3_B5=0

C5_B5=0

C6_B5=0

ILOOP=57

DO WHILE(ILOOP<=70)

C2_B5=C2_B5+(AMT9/V1)*(KA/(KA-KEL))*(EXP(-KEL*DOSETIME(ILOOP*2-1))-EXP(-KA*DOSETIME(ILOOP*2-1))) &

+(AMT10/V1)*(KA/(KA-KEL))*(EXP(-KEL*DOSETIME(ILOOP*2))-EXP(-KA*DOSETIME(ILOOP*2)))

C3_B5=C3_B5+((AMT9*KA*KEL2)/V2)*(AA*EXP(-KA*DOSETIME(ILOOP*2-1))+BB*EXP(-KEL*DOSETIME(ILOOP*2-1))+CC*EXP(-KEL2E*DOSETIME(ILOOP*2-1))) &

+((AMT10*KA*KEL2)/V2)*(AA*EXP(-KA*DOSETIME(ILOOP*2))+BB*EXP(-KEL*DOSETIME(ILOOP*2))+CC*EXP(-KEL2E*DOSETIME(ILOOP*2)))

C5_B5=C5_B5+((AMT9*KA*KEL3)/V2)*(DD*EXP(-KA*DOSETIME(ILOOP*2-1))+EE*EXP(-KEL*DOSETIME(ILOOP*2-1))+FF*EXP(-KEL3E*DOSETIME(ILOOP*2-1))) &

+((AMT10*KA*KEL3)/V2)*(DD*EXP(-KA*DOSETIME(ILOOP*2))+EE*EXP(-KEL*DOSETIME(ILOOP*2))+FF*EXP(-KEL3E*DOSETIME(ILOOP*2)))

C6_B5=C6_B5+((AMT9*KA*KEL4)/V2)*(GG*EXP(-KA*DOSETIME(ILOOP*2-1))+HH*EXP(-KEL*DOSETIME(ILOOP*2-1))+JJ*EXP(-KEL4E*DOSETIME(ILOOP*2-1))) &

+((AMT10*KA*KEL4)/V2)*(GG*EXP(-KA*DOSETIME(ILOOP*2))+HH*EXP(-KEL*DOSETIME(ILOOP*2))+JJ*EXP(-KEL4E*DOSETIME(ILOOP*2)))

ILOOP=ILOOP+1

ENDDO

C2_B6=0

C3_B6=0

C5_B6=0

C6_B6=0

ILOOP=71

DO WHILE(ILOOP<=85)

C2_B6=C2_B6+(AMT11/V1)*(KA/(KA-KEL))*(EXP(-KEL*DOSETIME(ILOOP*2-1))-EXP(-KA*DOSETIME(ILOOP*2-1))) &

+(AMT12/V1)*(KA/(KA-KEL))*(EXP(-KEL*DOSETIME(ILOOP*2))-EXP(-KA*DOSETIME(ILOOP*2)))

C3_B6=C3_B6+((AMT11*KA*KEL2)/V2)*(AA*EXP(-KA*DOSETIME(ILOOP*2-1))+BB*EXP(-KEL*DOSETIME(ILOOP*2-1))+CC*EXP(-KEL2E*DOSETIME(ILOOP*2-1))) &

+((AMT12*KA*KEL2)/V2)*(AA*EXP(-KA*DOSETIME(ILOOP*2))+BB*EXP(-KEL*DOSETIME(ILOOP*2))+CC*EXP(-KEL2E*DOSETIME(ILOOP*2)))

C5_B6=C5_B6+((AMT11*KA*KEL3)/V2)*(DD*EXP(-KA*DOSETIME(ILOOP*2-1))+EE*EXP(-KEL*DOSETIME(ILOOP*2-1))+FF*EXP(-KEL3E*DOSETIME(ILOOP*2-1))) &

+((AMT12*KA*KEL3)/V2)*(DD*EXP(-KA*DOSETIME(ILOOP*2))+EE*EXP(-KEL*DOSETIME(ILOOP*2))+FF*EXP(-KEL3E*DOSETIME(ILOOP*2)))

C6_B6=C6_B6+((AMT11*KA*KEL4)/V2)*(GG*EXP(-KA*DOSETIME(ILOOP*2-1))+HH*EXP(-KEL*DOSETIME(ILOOP*2-1))+JJ*EXP(-KEL4E*DOSETIME(ILOOP*2-1))) &

+((AMT12*KA*KEL4)/V2)*(GG*EXP(-KA*DOSETIME(ILOOP*2))+HH*EXP(-KEL*DOSETIME(ILOOP*2))+JJ*EXP(-KEL4E*DOSETIME(ILOOP*2)))

ILOOP=ILOOP+1

ENDDO

C2=C2_B1+C2_B2+C2_B3+C2_B4+C2_B5+C2_B6

C3=C3_B1+C3_B2+C3_B3+C3_B4+C3_B5+C3_B6

C5=C5_B1+C5_B2+C5_B3+C5_B4+C5_B5+C5_B6

C6=C6_B1+C6_B2+C6_B3+C6_B4+C6_B5+C6_B6

IF(GRP.EQ.1) THEN

C2=(AMT1/V1)*(KA/(KA-KEL))*(EXP(-KEL*TIME)-EXP(-KA*TIME))

C3=((AMT1*KA*KEL2)/V2)*(AA*EXP(-KA*TIME)+BB*EXP(-KEL*TIME)+CC*EXP(-KEL2E*TIME))

C5=((AMT1*KA*KEL3)/V2)*(DD*EXP(-KA*TIME)+EE*EXP(-KEL*TIME)+FF*EXP(-KEL3E*TIME))

C6=((AMT1*KA*KEL4)/V2)*(GG*EXP(-KA*TIME)+HH*EXP(-KEL*TIME)+JJ*EXP(-KEL4E*TIME))

ENDIF

; error models (separate SIGMA for each metabolite)

IF(CMT.EQ.2) THEN

Y=C2*EXP(THETA(11)*EPS(1)) ;exponential error model

IPRED=C2

ENDIF

IF(CMT.EQ.3) THEN

Y=C3*EXP(THETA(12)*EPS(2)) ;exponential error model

IPRED=C3

ENDIF

IF(CMT.EQ.5) THEN

Y=C5*EXP(THETA(13)*EPS(3)) ;exponential error model

IPRED=C5

ENDIF

IF(CMT.EQ.6) THEN

Y=C6*EXP(THETA(14)*EPS(4)) ;exponential error model

IPRED=C6

ENDIF

$THETA (0, 2) ; CLtot

(0, 160) ; V1

(0, 0.5) ; KA_star

(0.64) FIX ; FM1

(0, 19) ; CL2E

(0, 300) ; CL3E

(0, 250) ; CL4E

(0, 70) ; V2

(0, 1.5) ; Ind.effect1

(0, 2) ; Ind.effect2

;;;;;;;;;;;;;;;;;;;;;;;;;;

(0.222); CMT2 resid err.

(0.317); CMT3 resid err.

(0.295); CMT5 resid err.

(0.34); CMT6 resid err.

;;;;;;;;;;;;;;;;;;;;;;;;;;

(0,1.36); cov.eff.1.DGRP

(0,0.466); cov.eff.2

(0,1.96); cov.eff.3

$OMEGA BLOCK(2) 0.065 ; IIV CLtot

0.03 0.022 ; IIV V1

$OMEGA

0.716 ; IIV KA_star

0.128 ; IIV FM1

0.121 ; IIV CL2E

0.031 ; IIV CL3E

0.056 ; IIV CL4E

1.42 ; IIV V2

$SIGMA

1 FIX

1 FIX

1 FIX

1 FIX

NM-TRAN MESSAGES

WARNINGS AND ERRORS (IF ANY) FOR PROBLEM 1

(WARNING 2) NM-TRAN INFERS THAT THE DATA ARE POPULATION.

(WARNING 3) THERE MAY BE AN ERROR IN THE ABBREVIATED CODE. THE FOLLOWING

ONE OR MORE RANDOM VARIABLES ARE DEFINED WITH "IF" STATEMENTS THAT DO NOT

PROVIDE DEFINITIONS FOR BOTH THE "THEN" AND "ELSE" CASES. IF ALL

CONDITIONS FAIL, THE VALUES OF THESE VARIABLES WILL BE ZERO.

IPRED Y

(WARNING 13) WITH USER-WRITTEN PRED OR $PRED, NM-TRAN CANNOT APPEND THE

MDV DATA ITEM.

License Registered to: University of Liverpool

Expiration Date: 14 FEB 2021

Current Date: 13 OCT 2020

Days until program expires : 126

1NONLINEAR MIXED EFFECTS MODEL PROGRAM (NONMEM) VERSION 7.3.0

ORIGINALLY DEVELOPED BY STUART BEAL, LEWIS SHEINER, AND ALISON BOECKMANN

CURRENT DEVELOPERS ARE ROBERT BAUER, ICON DEVELOPMENT SOLUTIONS,

AND ALISON BOECKMANN. IMPLEMENTATION, EFFICIENCY, AND STANDARDIZATION

PERFORMED BY NOUS INFOSYSTEMS.

PROBLEM NO.: 1

PK of Carbamazepine and metabolites

0DATA CHECKOUT RUN: NO

DATA SET LOCATED ON UNIT NO.: 2

THIS UNIT TO BE REWOUND: NO

NO. OF DATA RECS IN DATA SET: 864

NO. OF DATA ITEMS IN DATA SET: 89

ID DATA ITEM IS DATA ITEM NO.: 1

DEP VARIABLE IS DATA ITEM NO.: 5

0LABELS FOR DATA ITEMS:

ID TAD TIME CMT DV AMT1 OFF1 AMT2 OFF2 AMT3 OFF3 AMT4 OFF4 AMT5 OFF5 AMT6 OFF6 AMT7 OFF7 AMT8 OFF8 AMT9 OFF9 AMT10 OFF10

AMT11 OFF11 AMT12 OFF12 AGE GEN WT HT DGRP GRP IND SMK VAL PHT PHB FEL CLOB LTG LEV ZON TOP LAC PRE PERA OMEP STATIN

ABCB1CT_HOM ABCB1CT_HET ABCB1TC_HOM ABCB1TC_HET ABCB1GT_HOM ABCB1GT_HET ABCC2GA_HOM ABCC2GA_HET ABCC2CT_HOM ABCC2CT_HET

ABCC2CT2_HOM ABCC2CT2_HET UGTAG_HOM UGTAG_HET UGTAG2_HOM UGTAG2_HET UGTGA_HOM UGTGA_HET EPHXTC_HOM EPHXTC_HET EPHXAG_HOM

EPHXAG_HET HOM_2C83 HET_2C83 HOM_2C84 HET_2C84 HOM_2C192 HET_2C192 HOM_2B6 HET_2B6 HOM_3A4 HET_3A4 HOM_3A5 HET_3A5

MPO_HOM MPO_HET POR_HOM POR_HET

0(NONBLANK) LABELS FOR PRED-DEFINED ITEMS:

CLtot V1 FM1 CL2E CL3E CL4E V2 KA AA BB CC DD EE FF GG HH JJ Y IPRED

0FORMAT FOR DATA:

(17(5E13.0/),4E13.0)

TOT. NO. OF OBS RECS: 864

TOT. NO. OF INDIVIDUALS: 79

0LENGTH OF THETA: 17

0DEFAULT THETA BOUNDARY TEST OMITTED: NO

0OMEGA HAS BLOCK FORM:

1

1 1

0 0 2

0 0 0 3

0 0 0 0 4

0 0 0 0 0 5

0 0 0 0 0 0 6

0 0 0 0 0 0 0 7

0DEFAULT OMEGA BOUNDARY TEST OMITTED: NO

0SIGMA HAS SIMPLE DIAGONAL FORM WITH DIMENSION: 4

0DEFAULT SIGMA BOUNDARY TEST OMITTED: NO

0INITIAL ESTIMATE OF THETA:

LOWER BOUND INITIAL EST UPPER BOUND

0.0000E+00 0.2000E+01 0.1000E+07

0.0000E+00 0.1600E+03 0.1000E+07

0.0000E+00 0.5000E+00 0.1000E+07

0.6400E+00 0.6400E+00 0.6400E+00

0.0000E+00 0.1900E+02 0.1000E+07

0.0000E+00 0.3000E+03 0.1000E+07

0.0000E+00 0.2500E+03 0.1000E+07

0.0000E+00 0.7000E+02 0.1000E+07

0.0000E+00 0.1500E+01 0.1000E+07

0.0000E+00 0.2000E+01 0.1000E+07

-0.1000E+07 0.2220E+00 0.1000E+07

-0.1000E+07 0.3170E+00 0.1000E+07

-0.1000E+07 0.2950E+00 0.1000E+07

-0.1000E+07 0.3400E+00 0.1000E+07

0.0000E+00 0.1360E+01 0.1000E+07

0.0000E+00 0.4660E+00 0.1000E+07

0.0000E+00 0.1960E+01 0.1000E+07

0INITIAL ESTIMATE OF OMEGA:

BLOCK SET NO. BLOCK FIXED

1 NO

0.6500E-01

0.3000E-01 0.2200E-01

2 NO

0.7160E+00

3 NO

0.1280E+00

4 NO

0.1210E+00

5 NO

0.3100E-01

6 NO

0.5600E-01

7 NO

0.1420E+01

0INITIAL ESTIMATE OF SIGMA:

0.1000E+01

0.0000E+00 0.1000E+01

0.0000E+00 0.0000E+00 0.1000E+01

0.0000E+00 0.0000E+00 0.0000E+00 0.1000E+01

0SIGMA CONSTRAINED TO BE THIS INITIAL ESTIMATE

0COVARIANCE STEP OMITTED: NO

EIGENVLS. PRINTED: NO

SPECIAL COMPUTATION: NO

COMPRESSED FORMAT: NO

SIGDIGITS ETAHAT (SIGLO): -1

SIGDIGITS GRADIENTS (SIGL): -1

RELATIVE TOLERANCE (TOL): -1

ABSOLUTE TOLERANCE-ADVAN 9,13 ONLY (ATOL): -1

EXCLUDE COV FOR FOCE (NOFCOV): NO

RESUME COV ANALYSIS (RESUME): NO

0TABLES STEP OMITTED: NO

NO. OF TABLES: 3

SEED NUMBER (SEED): 11456

RANMETHOD:

MC SAMPLES (ESEED): 300

WRES SQUARE ROOT TYPE: EIGENVALUE

0-- TABLE 1 --

04 COLUMNS APPENDED: NO

PRINTED: NO

HEADER: YES

FILE TO BE FORWARDED: NO

FORMAT: S1PE11.4

LFORMAT:

RFORMAT:

0USER-CHOSEN ITEMS:

ID TAD TIME DV Y PRED IPRED CMT CLtot FM1 CL2E CL3E CL4E V1 V2 KA RES WRES CWRES AA BB CC DD EE FF GG HH JJ

0-- TABLE 2 --

04 COLUMNS APPENDED: NO

PRINTED: NO

HEADER: YES

FILE TO BE FORWARDED: NO

FORMAT: S1PE11.4

LFORMAT:

RFORMAT:

0USER-CHOSEN ITEMS:

ID TAD TIME DV PRED IPRED CMT CLtot FM1 CL2E CL3E CL4E V1 V2 KA DGRP GRP

0-- TABLE 3 --

0FIRST RECORDS ONLY: YES

04 COLUMNS APPENDED: NO

PRINTED: NO

HEADER: YES

FILE TO BE FORWARDED: NO

FORMAT: S1PE11.4

LFORMAT:

RFORMAT:

0USER-CHOSEN ITEMS:

ID TAD CLtot FM1 CL2E CL3E CL4E V1 V2 KA AGE GEN WT HT DGRP GRP IND

1

#TBLN: 1

#METH: First Order Conditional Estimation with Interaction

ESTIMATION STEP OMITTED: NO

ANALYSIS TYPE: POPULATION

CONDITIONAL ESTIMATES USED: YES

CENTERED ETA: NO

EPS-ETA INTERACTION: YES

LAPLACIAN OBJ. FUNC.: NO

NO. OF FUNCT. EVALS. ALLOWED: 99999

NO. OF SIG. FIGURES REQUIRED: 3

INTERMEDIATE PRINTOUT: YES

ESTIMATE OUTPUT TO MSF: NO

ABORT WITH PRED EXIT CODE 1: NO

IND. OBJ. FUNC. VALUES SORTED: NO

NUMERICAL DERIVATIVE

FILE REQUEST (NUMDER): NONE

MAP (ETAHAT) ESTIMATION METHOD (OPTMAP): 0

ETA HESSIAN EVALUATION METHOD (ETADER): 0

INITIAL ETA FOR MAP ESTIMATION (MCETA): 0

SIGDIGITS FOR MAP ESTIMATION (SIGLO): 100

GRADIENT SIGDIGITS OF

FIXED EFFECTS PARAMETERS (SIGL): 100

EXCLUDE TITLE (NOTITLE): NO

EXCLUDE COLUMN LABELS (NOLABEL): NO

NOPRIOR SETTING (NOPRIOR): OFF

NOCOV SETTING (NOCOV): OFF

DERCONT SETTING (DERCONT): OFF

ABSOLUTE TOLERANCE-ADVAN 9,13 ONLY(ATOL):-100

FINAL ETA RE-EVALUATION (FNLETA): ON

EXCLUDE NON-INFLUENTIAL (NON-INFL.) ETAS

IN SHRINKAGE (ETASTYPE): NO

NON-INFL. ETA CORRECTION (NONINFETA): OFF

FORMAT FOR ADDITIONAL FILES (FORMAT): S1PE12.5

PARAMETER ORDER FOR OUTPUTS (ORDER): TSOL

ADDITIONAL CONVERGENCE TEST (CTYPE=4)?: NO

EM OR BAYESIAN METHOD USED: NONE

THE FOLLOWING LABELS ARE EQUIVALENT

PRED=PREDI

RES=RESI

WRES=WRESI

IWRS=IWRESI

IPRD=IPREDI

IRS=IRESI

MONITORING OF SEARCH:

0ITERATION NO.: 0 OBJECTIVE VALUE: -14560.7499082555 NO. OF FUNC. EVALS.: 18

CUMULATIVE NO. OF FUNC. EVALS.: 18

NPARAMETR: 2.0000E+00 1.6000E+02 5.0000E-01 1.9000E+01 3.0000E+02 2.5000E+02 7.0000E+01 1.5000E+00 2.0000E+00 2.2200E-01

3.1700E-01 2.9500E-01 3.4000E-01 1.3600E+00 4.6600E-01 1.9600E+00 6.5000E-02 3.0000E-02 2.2000E-02 7.1600E-01

1.2800E-01 1.2100E-01 3.1000E-02 5.6000E-02 1.4200E+00

PARAMETER: 1.0000E-01 1.0000E-01 1.0000E-01 1.0000E-01 1.0000E-01 1.0000E-01 1.0000E-01 1.0000E-01 1.0000E-01 1.0000E-01

1.0000E-01 1.0000E-01 1.0000E-01 1.0000E-01 1.0000E-01 1.0000E-01 1.0000E-01 1.0000E-01 1.0000E-01 1.0000E-01

1.0000E-01 1.0000E-01 1.0000E-01 1.0000E-01 1.0000E-01

GRADIENT: -4.6784E+01 -5.9620E+01 6.5693E+00 1.3273E+02 -2.0558E+02 -2.9614E+02 1.3744E+00 -1.6068E+02 -1.2918E+02 -4.1787E+01

-2.0046E+01 -5.5589E+02 -9.7588E+00 -4.0279E+01 6.5840E+00 -3.4774E+00 -1.6505E+01 6.4880E+01 -1.9135E+00 -2.1852E+00

-2.6013E+02 -2.9316E+01 -6.9508E+00 -2.7667E+01 -3.3131E+00

0ITERATION NO.: 4 OBJECTIVE VALUE: -14675.7793914831 NO. OF FUNC. EVALS.: 20

CUMULATIVE NO. OF FUNC. EVALS.: 96

NPARAMETR: 2.1308E+00 1.7035E+02 4.9562E-01 1.5821E+01 3.8403E+02 3.7304E+02 6.9888E+01 1.8198E+00 2.3262E+00 2.3712E-01

3.3706E-01 2.4867E-01 2.9784E-01 1.4242E+00 4.6175E-01 1.9684E+00 6.7438E-02 9.8110E-03 9.6133E-03 7.1821E-01

2.4155E-01 1.3065E-01 3.1306E-02 6.0195E-02 1.4346E+00

PARAMETER: 1.6335E-01 1.6267E-01 9.1208E-02 -8.3105E-02 3.4694E-01 5.0022E-01 9.8397E-02 2.9329E-01 2.5110E-01 1.0681E-01

1.0633E-01 8.4296E-02 8.7600E-02 1.4615E-01 9.0835E-02 1.0429E-01 1.1841E-01 3.2107E-02 1.0196E-01 1.0154E-01

4.1752E-01 1.3837E-01 1.0491E-01 1.3612E-01 1.0512E-01

GRADIENT: 1.9606E+02 8.0957E+01 3.0955E+00 1.0545E+02 1.1625E+02 -3.6862E+01 4.0362E+00 9.6850E+01 1.7450E+02 -6.5718E+01

1.0045E+01 -1.7008E+03 -8.3257E+02 -2.9298E+01 8.1645E+00 5.1271E+00 -1.3059E+01 -2.3896E+02 -6.4215E+00 7.2928E-01

6.4016E+00 -4.4318E+01 -2.8848E+01 -1.7108E+00 -3.5281E+00

0ITERATION NO.: 8 OBJECTIVE VALUE: -14705.7453307750 NO. OF FUNC. EVALS.: 20

CUMULATIVE NO. OF FUNC. EVALS.: 177

NPARAMETR: 1.8678E+00 1.5762E+02 4.8377E-01 1.1453E+01 4.3870E+02 4.4457E+02 6.8819E+01 1.8289E+00 2.0684E+00 2.2866E-01

3.4641E-01 2.3437E-01 2.9784E-01 1.4680E+00 4.4865E-01 1.9632E+00 6.7034E-02 3.7315E-02 2.8929E-02 7.1728E-01

2.4630E-01 1.6539E-01 3.4397E-02 6.6530E-02 1.4976E+00

PARAMETER: 3.1602E-02 8.4994E-02 6.7002E-02 -4.0618E-01 4.8004E-01 6.7565E-01 8.2982E-02 2.9826E-01 1.3362E-01 1.0300E-01

1.0928E-01 7.9449E-02 8.7599E-02 1.7642E-01 6.2049E-02 1.0161E-01 1.1541E-01 1.2248E-01 1.0026E-01 1.0089E-01

4.2726E-01 2.5626E-01 1.5199E-01 1.8615E-01 1.2659E-01

GRADIENT: 3.2336E+01 -2.9543E+01 6.2799E+00 -5.5946E+01 -2.3371E+01 6.0179E+01 6.1864E+00 1.0321E+01 1.3064E+01 5.2607E+01

9.8244E+01 -1.8024E+03 -9.1107E+02 6.0991E+00 2.1782E+00 7.6146E-01 -8.7782E-01 2.5535E+01 -6.7548E-01 -3.0211E+00

4.6103E+01 -1.7186E+01 -2.2906E+01 2.4699E+00 -4.3681E+00

0ITERATION NO.: 12 OBJECTIVE VALUE: -14724.7979408353 NO. OF FUNC. EVALS.: 20

CUMULATIVE NO. OF FUNC. EVALS.: 257

NPARAMETR: 1.9944E+00 1.6494E+02 4.1204E-01 8.7411E+00 6.6893E+02 6.5599E+02 6.0261E+01 1.7555E+00 1.6326E+00 2.4635E-01

3.0821E-01 2.7770E-01 3.2173E-01 1.3109E+00 4.0411E-01 1.9532E+00 4.8758E-02 7.7619E-03 8.7242E-03 7.0357E-01

6.1635E-02 2.8162E-01 8.3717E-02 1.1979E-01 2.0192E+00

PARAMETER: 9.7211E-02 1.3039E-01 -9.3476E-02 -6.7640E-01 9.0189E-01 1.0647E+00 -4.9808E-02 2.5729E-01 -1.0297E-01 1.1097E-01

9.7228E-02 9.4137E-02 9.4627E-02 6.3267E-02 -4.2508E-02 9.6507E-02 -4.3755E-02 2.9873E-02 5.7444E-02 9.1245E-02

-2.6540E-01 5.2239E-01 5.9673E-01 4.8021E-01 2.7603E-01

GRADIENT: 5.1393E+01 1.0572E+02 -9.3947E-01 -5.2302E+01 -3.8573E+01 1.7121E+02 5.0645E+00 3.4734E+01 -4.9802E+01 1.9615E+02

1.0361E+02 -5.9466E+02 -8.0675E+02 -1.4219E+01 -1.0381E+00 2.2253E+00 -4.4168E+00 -2.1886E+02 -5.0749E+00 3.3204E+00

6.2022E+01 2.0073E+01 -2.4884E+01 -1.6108E+01 -2.1087E+00

0ITERATION NO.: 16 OBJECTIVE VALUE: -14742.7070236845 NO. OF FUNC. EVALS.: 19

CUMULATIVE NO. OF FUNC. EVALS.: 335

NPARAMETR: 2.4284E+00 1.6765E+02 3.4610E-01 9.3357E+00 9.8795E+02 7.1012E+02 3.7519E+01 1.4175E+00 1.1542E+00 2.3618E-01

3.0629E-01 2.8591E-01 3.3168E-01 1.6075E+00 3.8792E-01 1.9979E+00 7.9325E-02 2.8494E-02 1.4115E-02 5.8067E-01

4.6216E-02 1.5735E-01 2.1540E-01 2.4003E-01 3.1730E+00

PARAMETER: 2.9407E-01 1.4669E-01 -2.6787E-01 -6.1059E-01 1.2919E+00 1.1440E+00 -5.2366E-01 4.3425E-02 -4.4972E-01 1.0639E-01

9.6620E-02 9.6919E-02 9.7552E-02 2.6718E-01 -8.3386E-02 1.1916E-01 1.9958E-01 8.5977E-02 -2.7142E-01 -4.7445E-03

-4.0935E-01 2.3135E-01 1.0693E+00 8.2770E-01 5.0202E-01

GRADIENT: 2.8809E+01 5.7528E+01 -4.9242E+00 -3.6959E+01 -1.6832E+00 1.2766E+02 2.5430E-01 1.8272E+01 -4.3322E+01 3.1005E+02

1.9615E+02 -1.1137E+02 -5.2785E+02 2.4888E+01 -3.1009E+00 1.5877E+00 7.4346E-01 -9.9200E+01 -8.6815E-01 1.7392E+00

6.8727E+01 1.5191E+01 -4.0854E+00 1.3851E+01 3.8637E+00

0ITERATION NO.: 20 OBJECTIVE VALUE: -14772.3200182454 NO. OF FUNC. EVALS.: 19

CUMULATIVE NO. OF FUNC. EVALS.: 415

NPARAMETR: 2.0892E+00 1.6777E+02 5.0818E-01 1.0044E+01 8.1659E+02 6.3232E+02 2.1853E+01 1.6625E+00 1.4331E+00 2.3253E-01

3.0437E-01 2.9763E-01 3.6186E-01 1.4006E+00 4.4072E-01 1.7015E+00 7.9907E-02 4.9677E-02 3.1530E-02 1.0173E+00

4.2015E-02 1.3315E-01 2.0284E-01 1.3605E-01 2.0746E+00

PARAMETER: 1.4364E-01 1.4743E-01 1.1623E-01 -5.3747E-01 1.1014E+00 1.0279E+00 -1.0642E+00 2.0285E-01 -2.3329E-01 1.0474E-01

9.6017E-02 1.0089E-01 1.0643E-01 1.2941E-01 4.4232E-02 -4.1453E-02 2.0324E-01 1.4935E-01 -1.1676E+00 2.7563E-01

-4.5700E-01 1.4786E-01 1.0392E+00 5.4385E-01 2.8955E-01

GRADIENT: -1.5438E+01 3.7528E+01 5.8413E+00 1.8159E+00 5.2152E+00 1.1029E+02 -4.7233E+00 1.3232E+01 -1.6546E+01 2.7999E+02

-6.0927E+01 1.4932E+02 -1.1490E+02 1.6540E+01 4.8443E-01 -3.1957E+00 5.4237E+00 4.1162E+01 -1.6393E-01 5.4227E+00

6.3552E+01 8.6630E+00 9.6274E+00 -7.4011E+00 -4.6495E+00

0ITERATION NO.: 24 OBJECTIVE VALUE: -14799.3276253164 NO. OF FUNC. EVALS.: 19

CUMULATIVE NO. OF FUNC. EVALS.: 496

NPARAMETR: 1.8968E+00 1.5635E+02 4.2105E-01 1.0167E+01 6.6810E+02 5.7542E+02 3.5615E+01 1.8169E+00 1.8304E+00 2.1704E-01

3.0823E-01 3.2486E-01 3.8198E-01 1.3368E+00 4.9799E-01 2.0917E+00 6.7642E-02 3.4895E-02 2.2551E-02 4.7210E-01

2.0936E-02 8.7589E-02 1.3895E-01 1.0238E-01 1.8847E+00

PARAMETER: 4.7005E-02 7.6929E-02 -7.1866E-02 -5.2531E-01 9.0065E-01 9.3363E-01 -5.7572E-01 2.9166E-01 1.1384E-02 9.7764E-02

9.7232E-02 1.1012E-01 1.1235E-01 8.2774E-02 1.6638E-01 1.6504E-01 1.1992E-01 1.1402E-01 -1.9175E-01 -1.0825E-01

-8.0527E-01 -6.1569E-02 8.5006E-01 4.0169E-01 2.4157E-01

GRADIENT: 1.0934E+01 -5.1268E+00 1.8662E+00 3.0925E+01 2.4931E+01 5.1216E+01 -6.3177E-01 2.9486E+01 3.5241E+01 -7.4934E+01

-1.4589E+02 3.0045E+02 1.1824E+02 -6.9592E-01 3.3910E+00 3.2162E+00 5.7518E+00 4.0803E+00 1.0456E+00 -3.9414E+00

3.8718E+01 -2.1366E+00 -2.9191E+00 -9.5102E+00 -2.5606E+00

0ITERATION NO.: 28 OBJECTIVE VALUE: -14812.2587356914 NO. OF FUNC. EVALS.: 20

CUMULATIVE NO. OF FUNC. EVALS.: 574

NPARAMETR: 1.9353E+00 1.5849E+02 3.6761E-01 9.5900E+00 7.3211E+02 5.5064E+02 3.2474E+01 1.7546E+00 1.7177E+00 2.1326E-01

3.1145E-01 3.2236E-01 3.8012E-01 1.3056E+00 5.0084E-01 1.8606E+00 6.4111E-02 3.5694E-02 2.4179E-02 6.1847E-01

9.0011E-03 7.6843E-02 1.5932E-01 1.0242E-01 2.9019E+00

PARAMETER: 6.7118E-02 9.0504E-02 -2.0758E-01 -5.8372E-01 9.9215E-01 8.8963E-01 -6.6805E-01 2.5675E-01 -5.2144E-02 9.6063E-02

9.8249E-02 1.0928E-01 1.1180E-01 5.9165E-02 1.7210E-01 4.7945E-02 9.3111E-02 1.1980E-01 -2.1914E-01 2.6782E-02

-1.2273E+00 -1.2701E-01 9.1847E-01 4.0189E-01 4.5736E-01

GRADIENT: 2.9962E+00 -5.7867E-01 -3.5982E+00 -9.6705E+00 3.6160E+01 8.3226E+00 -6.2856E-01 -1.8320E+00 3.9933E+01 -1.7886E+02

2.3072E+01 2.9539E+02 1.6415E+02 -1.2924E+01 5.7508E-01 -1.6144E+00 -3.9089E-01 8.1566E+00 9.7743E-01 8.3771E-01

1.3213E+01 1.2392E+00 -2.0456E+00 -5.6873E+00 3.2045E+00

0ITERATION NO.: 32 OBJECTIVE VALUE: -14818.9161129959 NO. OF FUNC. EVALS.: 19

CUMULATIVE NO. OF FUNC. EVALS.: 650

NPARAMETR: 1.9567E+00 1.6054E+02 4.0889E-01 9.7100E+00 7.3379E+02 5.3793E+02 3.6371E+01 1.7294E+00 1.6338E+00 2.2111E-01

3.0994E-01 2.9809E-01 3.6025E-01 1.3642E+00 4.9654E-01 1.9432E+00 6.4116E-02 3.2736E-02 1.9594E-02 6.0528E-01

4.8207E-03 7.6873E-02 1.8141E-01 1.2247E-01 2.3320E+00

PARAMETER: 7.8108E-02 1.0334E-01 -1.0116E-01 -5.7128E-01 9.9444E-01 8.6627E-01 -5.5471E-01 2.4232E-01 -1.0222E-01 9.9599E-02

9.7774E-02 1.0105E-01 1.0596E-01 1.0305E-01 1.6347E-01 9.1386E-02 9.3155E-02 1.0987E-01 -4.2050E-01 1.6004E-02

-1.5396E+00 -1.2682E-01 9.8338E-01 4.9125E-01 3.4803E-01

GRADIENT: -5.6057E-01 2.0408E+00 1.8342E-01 1.6278E+00 9.3894E+00 -6.5982E+00 -2.6493E-01 2.7931E+00 5.6721E+00 -4.1169E+00

5.9797E+00 3.9158E+01 6.6827E+00 1.3642E+00 -2.3351E-01 2.2320E-01 1.4652E-01 -2.0299E+00 3.2614E-01 7.1937E-01

1.8203E+00 4.2367E-01 6.8416E-01 2.4772E+00 5.7104E-01

0ITERATION NO.: 36 OBJECTIVE VALUE: -14819.1540345773 NO. OF FUNC. EVALS.: 19

CUMULATIVE NO. OF FUNC. EVALS.: 726

NPARAMETR: 1.9510E+00 1.6081E+02 4.0708E-01 9.6890E+00 7.1056E+02 5.4213E+02 3.7894E+01 1.7329E+00 1.6619E+00 2.2135E-01

3.1046E-01 2.9637E-01 3.6100E-01 1.3600E+00 5.0295E-01 1.9394E+00 6.4239E-02 3.3502E-02 1.9004E-02 5.7375E-01

4.0480E-03 7.6830E-02 1.7641E-01 1.1861E-01 2.2353E+00

PARAMETER: 7.5180E-02 1.0506E-01 -1.0559E-01 -5.7345E-01 9.6227E-01 8.7404E-01 -5.1370E-01 2.4434E-01 -8.5174E-02 9.9705E-02

9.7938E-02 1.0047E-01 1.0618E-01 1.0002E-01 1.7630E-01 8.9441E-02 9.4108E-02 1.1233E-01 -7.3601E-01 -1.0747E-02

-1.6269E+00 -1.2709E-01 9.6943E-01 4.7526E-01 3.2687E-01

GRADIENT: -1.0938E-02 -8.7256E-01 -1.0534E-01 -1.9935E-01 -4.7487E-01 4.1328E-01 -6.2728E-02 -3.7112E-01 -4.0403E-01 1.8969E+00

4.2795E+00 -5.3295E+00 1.2672E-02 -9.6862E-02 4.6892E-03 1.2462E-01 9.3388E-02 1.7836E+00 4.6160E-02 1.4078E-02

1.3131E-01 -1.8536E-01 7.6582E-02 -1.4284E-01 -7.4198E-02

0ITERATION NO.: 40 OBJECTIVE VALUE: -14819.1635254409 NO. OF FUNC. EVALS.: 36

CUMULATIVE NO. OF FUNC. EVALS.: 820

NPARAMETR: 1.9519E+00 1.6093E+02 4.0869E-01 9.6935E+00 7.1079E+02 5.4174E+02 3.8261E+01 1.7329E+00 1.6630E+00 2.2138E-01

3.0994E-01 2.9696E-01 3.6112E-01 1.3601E+00 5.0328E-01 1.9309E+00 6.3952E-02 3.3289E-02 1.8613E-02 5.7051E-01

3.9216E-03 7.7357E-02 1.7617E-01 1.1893E-01 2.2441E+00

PARAMETER: 7.5631E-02 1.0580E-01 -1.0164E-01 -5.7298E-01 9.6260E-01 8.7333E-01 -5.0407E-01 2.4431E-01 -8.4510E-02 9.9723E-02

9.7773E-02 1.0066E-01 1.0621E-01 1.0006E-01 1.7696E-01 8.5032E-02 9.1876E-02 1.1187E-01 -8.2387E-01 -1.3572E-02

-1.6428E+00 -1.2368E-01 9.6873E-01 4.7658E-01 3.2883E-01

GRADIENT: -6.8121E-01 -5.2023E-01 -1.1646E-01 -9.9048E-01 -2.6915E+00 -1.8640E+00 -1.4792E-01 -7.0287E-01 -1.4489E-01 -4.0824E+00

-7.9577E+00 -3.3159E+00 -1.9542E+00 6.5951E-02 1.6146E-02 -1.8164E-02 -1.4171E-02 5.4751E-01 1.4183E-02 -2.9534E-03

-5.9168E-02 -1.0781E-01 -2.5335E-02 3.0961E-02 5.8392E-03

0ITERATION NO.: 44 OBJECTIVE VALUE: -14819.1966407758 NO. OF FUNC. EVALS.: 35

CUMULATIVE NO. OF FUNC. EVALS.: 962

NPARAMETR: 1.9613E+00 1.6104E+02 4.0943E-01 9.7075E+00 7.2555E+02 5.4346E+02 3.9164E+01 1.7258E+00 1.6346E+00 2.2136E-01

3.0997E-01 2.9692E-01 3.6156E-01 1.3584E+00 5.0223E-01 1.9315E+00 6.4142E-02 3.3464E-02 1.8630E-02 5.6734E-01

3.9619E-03 7.7860E-02 1.7922E-01 1.1914E-01 2.2411E+00

PARAMETER: 8.0444E-02 1.0647E-01 -9.9833E-02 -5.7154E-01 9.8315E-01 8.7649E-01 -4.8075E-01 2.4025E-01 -1.0175E-01 9.9714E-02

9.7782E-02 1.0065E-01 1.0634E-01 9.8802E-02 1.7488E-01 8.5346E-02 9.3354E-02 1.1229E-01 -8.7027E-01 -1.6364E-02

-1.6377E+00 -1.2044E-01 9.7732E-01 4.7749E-01 3.2814E-01

GRADIENT: -8.7381E-03 -5.1328E-03 -2.2269E-04 1.6971E-02 1.2046E-02 -7.2707E-03 -2.0517E-04 -1.1992E-02 6.5188E-03 3.6426E-02

-4.3103E-02 2.9877E-02 1.1924E-01 -5.5048E-03 2.2626E-04 -3.4059E-03 2.8145E-03 3.7329E-02 1.9912E-03 -5.4850E-04

-7.1494E-03 -5.6454E-04 -2.8860E-03 4.3395E-03 4.5743E-05

0ITERATION NO.: 48 OBJECTIVE VALUE: -14819.1966510754 NO. OF FUNC. EVALS.: 35

CUMULATIVE NO. OF FUNC. EVALS.: 1102

NPARAMETR: 1.9612E+00 1.6104E+02 4.0945E-01 9.7073E+00 7.2550E+02 5.4346E+02 3.9164E+01 1.7259E+00 1.6347E+00 2.2137E-01

3.0998E-01 2.9692E-01 3.6155E-01 1.3584E+00 5.0223E-01 1.9316E+00 6.4134E-02 3.3461E-02 1.8611E-02 5.6721E-01

3.9657E-03 7.7856E-02 1.7922E-01 1.1914E-01 2.2410E+00

PARAMETER: 8.0431E-02 1.0649E-01 -9.9797E-02 -5.7156E-01 9.8308E-01 8.7650E-01 -4.8074E-01 2.4027E-01 -1.0169E-01 9.9714E-02

9.7784E-02 1.0065E-01 1.0634E-01 9.8787E-02 1.7487E-01 8.5423E-02 9.3291E-02 1.1229E-01 -8.7831E-01 -1.6473E-02

-1.6372E+00 -1.2046E-01 9.7731E-01 4.7749E-01 3.2813E-01

GRADIENT: 9.0625E-04 2.4067E-05 -1.8126E-05 4.5399E-05 5.6273E-05 -2.8475E-04 2.0954E-05 4.1676E-05 -9.4035E-05 -3.5883E-03

1.1860E-03 5.0589E-03 4.3384E-03 -3.8815E-04 -8.8231E-05 2.4920E-05 -4.6735E-04 1.5254E-03 3.3004E-05 6.4293E-05

-5.9627E-05 1.0236E-04 -2.5399E-04 3.9412E-04 -1.1887E-04

0ITERATION NO.: 49 OBJECTIVE VALUE: -14819.1966510754 NO. OF FUNC. EVALS.: 32

CUMULATIVE NO. OF FUNC. EVALS.: 1134

NPARAMETR: 1.9612E+00 1.6104E+02 4.0945E-01 9.7073E+00 7.2550E+02 5.4346E+02 3.9164E+01 1.7259E+00 1.6347E+00 2.2137E-01

3.0998E-01 2.9692E-01 3.6155E-01 1.3584E+00 5.0223E-01 1.9316E+00 6.4134E-02 3.3461E-02 1.8611E-02 5.6721E-01

3.9657E-03 7.7856E-02 1.7922E-01 1.1914E-01 2.2410E+00

PARAMETER: 8.0431E-02 1.0649E-01 -9.9797E-02 -5.7156E-01 9.8308E-01 8.7650E-01 -4.8074E-01 2.4027E-01 -1.0169E-01 9.9714E-02

9.7784E-02 1.0065E-01 1.0634E-01 9.8787E-02 1.7487E-01 8.5423E-02 9.3291E-02 1.1229E-01 -8.7831E-01 -1.6473E-02

-1.6372E+00 -1.2046E-01 9.7731E-01 4.7749E-01 3.2813E-01

GRADIENT: 9.0625E-04 2.4067E-05 -1.8126E-05 4.5399E-05 5.6273E-05 -2.8475E-04 2.0954E-05 4.1676E-05 -9.4035E-05 -3.5883E-03

1.1860E-03 5.0589E-03 4.3384E-03 -3.8815E-04 -8.8231E-05 2.4920E-05 -4.6735E-04 1.5254E-03 3.3004E-05 6.4293E-05

-5.9627E-05 1.0236E-04 -2.5399E-04 3.9412E-04 -1.1887E-04

#TERM:

0MINIMIZATION SUCCESSFUL

NO. OF FUNCTION EVALUATIONS USED: 1134

NO. OF SIG. DIGITS IN FINAL EST.: 3.9

ETABAR IS THE ARITHMETIC MEAN OF THE ETA-ESTIMATES,

AND THE P-VALUE IS GIVEN FOR THE NULL HYPOTHESIS THAT THE TRUE MEAN IS 0.

ETABAR: 5.4279E-04 1.7569E-04 1.7342E-03 7.0694E-04 -1.4108E-03 6.4736E-04 5.7207E-04 3.0614E-03

SE: 2.2730E-02 1.1908E-02 3.1517E-02 2.4882E-03 2.2241E-02 4.0900E-02 2.9799E-02 5.9883E-02

N: 79 79 79 79 79 79 79 79

P VAL.: 9.8095E-01 9.8823E-01 9.5612E-01 7.7632E-01 9.4942E-01 9.8737E-01 9.8468E-01 9.5923E-01

ETAshrink(%): 1.9715E+01 2.1923E+01 6.2567E+01 6.4656E+01 2.8700E+01 1.3580E+01 2.2777E+01 6.4218E+01

EBVshrink(%): 2.0080E+01 2.2291E+01 6.2137E+01 6.5343E+01 2.8928E+01 1.4072E+01 2.3224E+01 6.4067E+01

EPSshrink(%): 1.2024E+01 1.1773E+01 1.2759E+01 1.0542E+01

#TERE:

Elapsed estimation time in seconds: 2686.13

Elapsed covariance time in seconds: 5029.53

1

************************************************************************************************************************

******************** ********************

******************** FIRST ORDER CONDITIONAL ESTIMATION WITH INTERACTION ********************

#OBJT:************** MINIMUM VALUE OF OBJECTIVE FUNCTION ********************

******************** ********************

************************************************************************************************************************

#OBJV:******************************************** -14819.197 **************************************************

1

************************************************************************************************************************

******************** ********************

******************** FIRST ORDER CONDITIONAL ESTIMATION WITH INTERACTION ********************

******************** FINAL PARAMETER ESTIMATE ********************

******************** ********************

************************************************************************************************************************

THETA - VECTOR OF FIXED EFFECTS PARAMETERS *********

TH 1 TH 2 TH 3 TH 4 TH 5 TH 6 TH 7 TH 8 TH 9 TH10 TH11 TH12

TH13 TH14 TH15 TH16 TH17

1.96E+00 1.61E+02 4.09E-01 6.40E-01 9.71E+00 7.26E+02 5.43E+02 3.92E+01 1.73E+00 1.63E+00 2.21E-01 3.10E-01

2.97E-01 3.62E-01 1.36E+00 5.02E-01 1.93E+00

OMEGA - COV MATRIX FOR RANDOM EFFECTS - ETAS ********

ETA1 ETA2 ETA3 ETA4 ETA5 ETA6 ETA7 ETA8

ETA1

+ 6.41E-02

ETA2

+ 3.35E-02 1.86E-02

ETA3

+ 0.00E+00 0.00E+00 5.67E-01

ETA4

+ 0.00E+00 0.00E+00 0.00E+00 3.97E-03

ETA5

+ 0.00E+00 0.00E+00 0.00E+00 0.00E+00 7.79E-02

ETA6

+ 0.00E+00 0.00E+00 0.00E+00 0.00E+00 0.00E+00 1.79E-01

ETA7

+ 0.00E+00 0.00E+00 0.00E+00 0.00E+00 0.00E+00 0.00E+00 1.19E-01

ETA8

+ 0.00E+00 0.00E+00 0.00E+00 0.00E+00 0.00E+00 0.00E+00 0.00E+00 2.24E+00

SIGMA - COV MATRIX FOR RANDOM EFFECTS - EPSILONS ****

EPS1 EPS2 EPS3 EPS4

EPS1

+ 1.00E+00

EPS2

+ 0.00E+00 1.00E+00

EPS3

+ 0.00E+00 0.00E+00 1.00E+00

EPS4

+ 0.00E+00 0.00E+00 0.00E+00 1.00E+00

1

OMEGA - CORR MATRIX FOR RANDOM EFFECTS - ETAS *******

ETA1 ETA2 ETA3 ETA4 ETA5 ETA6 ETA7 ETA8

ETA1

+ 2.53E-01

ETA2

+ 9.69E-01 1.36E-01

ETA3

+ 0.00E+00 0.00E+00 7.53E-01

ETA4

+ 0.00E+00 0.00E+00 0.00E+00 6.30E-02

ETA5

+ 0.00E+00 0.00E+00 0.00E+00 0.00E+00 2.79E-01

ETA6

+ 0.00E+00 0.00E+00 0.00E+00 0.00E+00 0.00E+00 4.23E-01

ETA7

+ 0.00E+00 0.00E+00 0.00E+00 0.00E+00 0.00E+00 0.00E+00 3.45E-01

ETA8

+ 0.00E+00 0.00E+00 0.00E+00 0.00E+00 0.00E+00 0.00E+00 0.00E+00 1.50E+00

SIGMA - CORR MATRIX FOR RANDOM EFFECTS - EPSILONS ***

EPS1 EPS2 EPS3 EPS4

EPS1

+ 1.00E+00

EPS2

+ 0.00E+00 1.00E+00

EPS3

+ 0.00E+00 0.00E+00 1.00E+00

EPS4

+ 0.00E+00 0.00E+00 0.00E+00 1.00E+00

1

************************************************************************************************************************

******************** ********************

******************** FIRST ORDER CONDITIONAL ESTIMATION WITH INTERACTION ********************

******************** STANDARD ERROR OF ESTIMATE ********************

******************** ********************

************************************************************************************************************************

THETA - VECTOR OF FIXED EFFECTS PARAMETERS *********

TH 1 TH 2 TH 3 TH 4 TH 5 TH 6 TH 7 TH 8 TH 9 TH10 TH11 TH12

TH13 TH14 TH15 TH16 TH17

3.60E-01 1.10E+01 9.92E-02 ......... 4.89E-01 1.82E+02 3.15E+01 1.77E+01 3.30E-01 4.33E-01 2.18E-02 1.92E-02

2.27E-02 4.19E-02 1.39E-01 7.22E-02 4.82E-01

OMEGA - COV MATRIX FOR RANDOM EFFECTS - ETAS ********

ETA1 ETA2 ETA3 ETA4 ETA5 ETA6 ETA7 ETA8

ETA1

+ 2.90E-02

ETA2

+ 1.63E-02 1.02E-02

ETA3

+ ......... ......... 2.18E-01

ETA4

+ ......... ......... ......... 4.94E-03

ETA5

+ ......... ......... ......... ......... 2.43E-02

ETA6

+ ......... ......... ......... ......... ......... 5.90E-02

ETA7

+ ......... ......... ......... ......... ......... ......... 3.27E-02

ETA8

+ ......... ......... ......... ......... ......... ......... ......... 1.36E+00

SIGMA - COV MATRIX FOR RANDOM EFFECTS - EPSILONS ****

EPS1 EPS2 EPS3 EPS4

EPS1

+ .........

EPS2

+ ......... .........

EPS3

+ ......... ......... .........

EPS4

+ ......... ......... ......... .........

1

OMEGA - CORR MATRIX FOR RANDOM EFFECTS - ETAS *******

ETA1 ETA2 ETA3 ETA4 ETA5 ETA6 ETA7 ETA8

ETA1

+ 5.73E-02

ETA2

+ 2.22E-01 3.73E-02

ETA3

+ ......... ......... 1.45E-01

ETA4

+ ......... ......... ......... 3.92E-02

ETA5

+ ......... ......... ......... ......... 4.36E-02

ETA6

+ ......... ......... ......... ......... ......... 6.97E-02

ETA7

+ ......... ......... ......... ......... ......... ......... 4.74E-02

ETA8

+ ......... ......... ......... ......... ......... ......... ......... 4.55E-01

SIGMA - CORR MATRIX FOR RANDOM EFFECTS - EPSILONS ***

EPS1 EPS2 EPS3 EPS4

EPS1

+ .........

EPS2

+ ......... .........

EPS3

+ ......... ......... .........

EPS4

+ ......... ......... ......... .........

1

************************************************************************************************************************

******************** ********************

******************** FIRST ORDER CONDITIONAL ESTIMATION WITH INTERACTION ********************

******************** COVARIANCE MATRIX OF ESTIMATE ********************

******************** ********************

************************************************************************************************************************

TH 1 TH 2 TH 3 TH 4 TH 5 TH 6 TH 7 TH 8 TH 9 TH10 TH11 TH12

TH13 TH14 TH15 TH16 TH17 OM11 OM12 OM13 OM14 OM15 OM16 OM17

OM18 OM22 OM23 OM24 OM25 OM26 OM27 OM28 OM33 OM34 OM35 OM36

OM37 OM38 OM44 OM45 OM46 OM47 OM48 OM55 OM56 OM57 OM58 OM66

OM67 OM68 OM77 OM78 OM88 SG11 SG12 SG13 SG14 SG22 SG23 SG24

SG33 SG34 SG44

TH 1

+ 1.30E-01

TH 2

+ 3.26E+00 1.22E+02

TH 3

+ 1.53E-02 6.85E-01 9.84E-03

TH 4

+ ......... ......... ......... .........

TH 5

+ 9.76E-02 2.99E+00 7.87E-03 ......... 2.39E-01

TH 6

+ 5.82E+01 1.37E+03 8.38E+00 ......... 3.75E+01 3.30E+04

TH 7

+ 5.87E+00 1.74E+02 1.05E+00 ......... 7.39E+00 3.12E+03 9.93E+02

TH 8

+ -2.61E+00 -3.97E+01 6.27E-01 ......... -1.88E+00 -9.71E+02 -3.14E+01 3.12E+02

TH 9

+ -1.16E-01 -2.72E+00 -1.36E-02 ......... -7.90E-02 -5.34E+01 -4.49E+00 2.58E+00 1.09E-01

TH10

+ -1.36E-01 -3.17E+00 -2.16E-02 ......... -7.77E-02 -7.64E+01 -5.59E+00 2.26E+00 1.29E-01 1.88E-01

TH11

+ -6.39E-04 5.27E-02 7.94E-04 ......... -9.57E-04 1.02E-01 1.16E-01 1.53E-01 1.06E-03 -2.22E-04 4.74E-04

TH12

+ 2.41E-03 6.28E-02 -6.08E-04 ......... 3.41E-03 1.75E-01 1.08E-01 -2.00E-01 -1.87E-03 -1.04E-04 -9.78E-05 3.70E-04

TH13

+ -6.54E-04 5.95E-03 -5.39E-04 ......... -9.95E-05 -7.53E-01 8.52E-02 -9.11E-03 9.14E-04 2.01E-03 2.44E-04 1.49E-04

5.17E-04

TH14

+ -5.87E-03 -4.40E-02 8.24E-04 ......... -3.06E-03 -3.66E+00 9.59E-02 4.21E-01 6.03E-03 8.53E-03 5.27E-04 -2.75E-05

4.38E-04 1.76E-03

TH15

+ 2.95E-02 4.89E-01 -5.36E-04 ......... 2.20E-02 8.13E+00 1.04E+00 -8.96E-01 -2.56E-02 -1.44E-02 -1.47E-03 1.33E-03

-4.20E-04 -2.01E-03 1.92E-02

TH16

+ -3.73E-03 -1.12E-01 -2.33E-04 ......... -1.22E-02 -1.24E+00 -1.71E-01 2.16E-02 3.04E-03 2.52E-03 9.79E-05 -1.52E-04

1.58E-05 5.12E-05 -8.60E-04 5.21E-03

TH17

+ 1.73E-02 3.68E-01 -2.51E-03 ......... 2.08E-02 7.81E+00 -9.68E-02 -6.70E-01 -2.05E-02 -2.10E-02 -7.48E-04 5.19E-04

-2.46E-04 -1.41E-03 4.92E-03 -8.48E-04 2.32E-01

OM11

+ 8.18E-03 2.18E-01 3.62E-04 ......... 7.16E-03 3.31E+00 2.94E-01 -2.56E-01 -7.25E-03 -7.81E-03 -1.19E-04 3.18E-04

6.00E-05 -4.26E-04 2.09E-03 -2.99E-04 3.06E-03 8.43E-04

1

TH 1 TH 2 TH 3 TH 4 TH 5 TH 6 TH 7 TH 8 TH 9 TH10 TH11 TH12

TH13 TH14 TH15 TH16 TH17 OM11 OM12 OM13 OM14 OM15 OM16 OM17

OM18 OM22 OM23 OM24 OM25 OM26 OM27 OM28 OM33 OM34 OM35 OM36

OM37 OM38 OM44 OM45 OM46 OM47 OM48 OM55 OM56 OM57 OM58 OM66

OM67 OM68 OM77 OM78 OM88 SG11 SG12 SG13 SG14 SG22 SG23 SG24

SG33 SG34 SG44

OM12

+ 4.67E-03 1.16E-01 1.14E-04 ......... 4.18E-03 1.96E+00 2.01E-01 -1.58E-01 -4.04E-03 -4.54E-03 -3.73E-05 1.99E-04

5.28E-05 -2.22E-04 1.09E-03 -1.70E-04 1.56E-03 4.52E-04 2.66E-04

OM13

+ ......... ......... ......... ......... ......... ......... ......... ......... ......... ......... ......... .........

......... ......... ......... ......... ......... ......... ......... .........

OM14

+ ......... ......... ......... ......... ......... ......... ......... ......... ......... ......... ......... .........

......... ......... ......... ......... ......... ......... ......... ......... .........

OM15

+ ......... ......... ......... ......... ......... ......... ......... ......... ......... ......... ......... .........

......... ......... ......... ......... ......... ......... ......... ......... ......... .........

OM16

+ ......... ......... ......... ......... ......... ......... ......... ......... ......... ......... ......... .........

......... ......... ......... ......... ......... ......... ......... ......... ......... ......... .........

OM17

+ ......... ......... ......... ......... ......... ......... ......... ......... ......... ......... ......... .........

......... ......... ......... ......... ......... ......... ......... ......... ......... ......... ......... .........

OM18

+ ......... ......... ......... ......... ......... ......... ......... ......... ......... ......... ......... .........

......... ......... ......... ......... ......... ......... ......... ......... ......... ......... ......... .........

.........

OM22

+ 1.26E-03 1.87E-02 -1.95E-04 ......... 1.50E-03 5.36E-01 6.39E-03 -9.59E-02 -1.06E-03 -1.14E-03 -9.30E-05 9.88E-05

-6.03E-05 -2.48E-04 5.73E-04 -3.75E-05 7.20E-04 1.79E-04 1.09E-04 ......... ......... ......... ......... .........

......... 1.03E-04

OM23

+ ......... ......... ......... ......... ......... ......... ......... ......... ......... ......... ......... .........

......... ......... ......... ......... ......... ......... ......... ......... ......... ......... ......... .........

......... ......... .........

OM24

+ ......... ......... ......... ......... ......... ......... ......... ......... ......... ......... ......... .........

......... ......... ......... ......... ......... ......... ......... ......... ......... ......... ......... .........

......... ......... ......... .........

OM25

+ ......... ......... ......... ......... ......... ......... ......... ......... ......... ......... ......... .........

......... ......... ......... ......... ......... ......... ......... ......... ......... ......... ......... .........

......... ......... ......... ......... .........

OM26

+ ......... ......... ......... ......... ......... ......... ......... ......... ......... ......... ......... .........

......... ......... ......... ......... ......... ......... ......... ......... ......... ......... ......... .........

......... ......... ......... ......... ......... .........

OM27

+ ......... ......... ......... ......... ......... ......... ......... ......... ......... ......... ......... .........

......... ......... ......... ......... ......... ......... ......... ......... ......... ......... ......... .........

......... ......... ......... ......... ......... ......... .........

1

TH 1 TH 2 TH 3 TH 4 TH 5 TH 6 TH 7 TH 8 TH 9 TH10 TH11 TH12

TH13 TH14 TH15 TH16 TH17 OM11 OM12 OM13 OM14 OM15 OM16 OM17

OM18 OM22 OM23 OM24 OM25 OM26 OM27 OM28 OM33 OM34 OM35 OM36

OM37 OM38 OM44 OM45 OM46 OM47 OM48 OM55 OM56 OM57 OM58 OM66

OM67 OM68 OM77 OM78 OM88 SG11 SG12 SG13 SG14 SG22 SG23 SG24

SG33 SG34 SG44

OM28

+ ......... ......... ......... ......... ......... ......... ......... ......... ......... ......... ......... .........

......... ......... ......... ......... ......... ......... ......... ......... ......... ......... ......... .........

......... ......... ......... ......... ......... ......... ......... .........

OM33

+ -2.51E-02 -2.67E-01 4.45E-03 ......... -3.12E-02 -6.63E-01 -2.92E-01 1.56E+00 2.48E-02 3.39E-03 2.89E-03 -2.13E-03

5.89E-04 5.90E-04 -1.65E-02 2.35E-03 -6.57E-04 -2.19E-03 -1.12E-03 ......... ......... ......... ......... .........

......... -2.26E-04 ......... ......... ......... ......... ......... ......... 4.74E-02

OM34

+ ......... ......... ......... ......... ......... ......... ......... ......... ......... ......... ......... .........

......... ......... ......... ......... ......... ......... ......... ......... ......... ......... ......... .........

......... ......... ......... ......... ......... ......... ......... ......... ......... .........

OM35

+ ......... ......... ......... ......... ......... ......... ......... ......... ......... ......... ......... .........

......... ......... ......... ......... ......... ......... ......... ......... ......... ......... ......... .........

......... ......... ......... ......... ......... ......... ......... ......... ......... ......... .........

OM36

+ ......... ......... ......... ......... ......... ......... ......... ......... ......... ......... ......... .........

......... ......... ......... ......... ......... ......... ......... ......... ......... ......... ......... .........

......... ......... ......... ......... ......... ......... ......... ......... ......... ......... ......... .........

OM37

+ ......... ......... ......... ......... ......... ......... ......... ......... ......... ......... ......... .........

......... ......... ......... ......... ......... ......... ......... ......... ......... ......... ......... .........

......... ......... ......... ......... ......... ......... ......... ......... ......... ......... ......... .........

.........

OM38

+ ......... ......... ......... ......... ......... ......... ......... ......... ......... ......... ......... .........

......... ......... ......... ......... ......... ......... ......... ......... ......... ......... ......... .........

......... ......... ......... ......... ......... ......... ......... ......... ......... ......... ......... .........

......... .........

OM44

+ -2.19E-06 -5.32E-03 -5.98E-05 ......... 2.04E-04 -1.18E-01 -1.29E-02 1.15E-02 -8.69E-06 2.12E-04 -2.70E-05 1.64E-05

5.39E-06 2.25E-05 9.83E-05 -7.99E-05 -5.02E-05 -9.35E-06 -5.01E-06 ......... ......... ......... ......... .........

......... -9.96E-06 ......... ......... ......... ......... ......... ......... -4.58E-04 ......... ......... .........

......... ......... 2.44E-05

OM45

+ ......... ......... ......... ......... ......... ......... ......... ......... ......... ......... ......... .........

......... ......... ......... ......... ......... ......... ......... ......... ......... ......... ......... .........

......... ......... ......... ......... ......... ......... ......... ......... ......... ......... ......... .........

......... ......... ......... .........

OM46

+ ......... ......... ......... ......... ......... ......... ......... ......... ......... ......... ......... .........

......... ......... ......... ......... ......... ......... ......... ......... ......... ......... ......... .........

......... ......... ......... ......... ......... ......... ......... ......... ......... ......... ......... .........

......... ......... ......... ......... .........

1

TH 1 TH 2 TH 3 TH 4 TH 5 TH 6 TH 7 TH 8 TH 9 TH10 TH11 TH12

TH13 TH14 TH15 TH16 TH17 OM11 OM12 OM13 OM14 OM15 OM16 OM17

OM18 OM22 OM23 OM24 OM25 OM26 OM27 OM28 OM33 OM34 OM35 OM36

OM37 OM38 OM44 OM45 OM46 OM47 OM48 OM55 OM56 OM57 OM58 OM66

OM67 OM68 OM77 OM78 OM88 SG11 SG12 SG13 SG14 SG22 SG23 SG24

SG33 SG34 SG44

OM47

+ ......... ......... ......... ......... ......... ......... ......... ......... ......... ......... ......... .........

......... ......... ......... ......... ......... ......... ......... ......... ......... ......... ......... .........

......... ......... ......... ......... ......... ......... ......... ......... ......... ......... ......... .........

......... ......... ......... ......... ......... .........

OM48

+ ......... ......... ......... ......... ......... ......... ......... ......... ......... ......... ......... .........

......... ......... ......... ......... ......... ......... ......... ......... ......... ......... ......... .........

......... ......... ......... ......... ......... ......... ......... ......... ......... ......... ......... .........

......... ......... ......... ......... ......... ......... .........

OM55

+ 4.83E-03 1.26E-01 9.49E-04 ......... 2.13E-03 2.70E+00 1.44E-01 -7.83E-02 -4.67E-03 -6.70E-03 -3.61E-05 -3.03E-05

-1.52E-04 -4.07E-04 6.06E-04 3.54E-05 1.96E-03 3.35E-04 1.63E-04 ......... ......... ......... ......... .........

......... 5.69E-05 ......... ......... ......... ......... ......... ......... 7.55E-06 ......... ......... .........

......... ......... -2.85E-05 ......... ......... ......... ......... 5.92E-04

OM56

+ ......... ......... ......... ......... ......... ......... ......... ......... ......... ......... ......... .........

......... ......... ......... ......... ......... ......... ......... ......... ......... ......... ......... .........

......... ......... ......... ......... ......... ......... ......... ......... ......... ......... ......... .........

......... ......... ......... ......... ......... ......... ......... ......... .........

OM57

+ ......... ......... ......... ......... ......... ......... ......... ......... ......... ......... ......... .........

......... ......... ......... ......... ......... ......... ......... ......... ......... ......... ......... .........

......... ......... ......... ......... ......... ......... ......... ......... ......... ......... ......... .........

......... ......... ......... ......... ......... ......... ......... ......... ......... .........

OM58

+ ......... ......... ......... ......... ......... ......... ......... ......... ......... ......... ......... .........

......... ......... ......... ......... ......... ......... ......... ......... ......... ......... ......... .........

......... ......... ......... ......... ......... ......... ......... ......... ......... ......... ......... .........

......... ......... ......... ......... ......... ......... ......... ......... ......... ......... .........

OM66

+ 1.07E-02 2.51E-01 2.25E-03 ......... 5.87E-03 6.50E+00 3.92E-01 -7.19E-02 -1.05E-02 -1.52E-02 -4.61E-05 -8.56E-05

-4.64E-04 -3.88E-04 1.74E-03 -1.57E-04 4.22E-03 7.41E-04 3.89E-04 ......... ......... ......... ......... .........

......... 1.30E-04 ......... ......... ......... ......... ......... ......... -1.84E-03 ......... ......... .........

......... ......... -1.96E-05 ......... ......... ......... ......... 7.75E-04 ......... ......... ......... 3.48E-03

OM67

+ ......... ......... ......... ......... ......... ......... ......... ......... ......... ......... ......... .........

......... ......... ......... ......... ......... ......... ......... ......... ......... ......... ......... .........

......... ......... ......... ......... ......... ......... ......... ......... ......... ......... ......... .........

......... ......... ......... ......... ......... ......... ......... ......... ......... ......... ......... .........

.........

OM68

+ ......... ......... ......... ......... ......... ......... ......... ......... ......... ......... ......... .........

......... ......... ......... ......... ......... ......... ......... ......... ......... ......... ......... .........

......... ......... ......... ......... ......... ......... ......... ......... ......... ......... ......... .........

......... ......... ......... ......... ......... ......... ......... ......... ......... ......... ......... .........

......... .........

1

TH 1 TH 2 TH 3 TH 4 TH 5 TH 6 TH 7 TH 8 TH 9 TH10 TH11 TH12

TH13 TH14 TH15 TH16 TH17 OM11 OM12 OM13 OM14 OM15 OM16 OM17

OM18 OM22 OM23 OM24 OM25 OM26 OM27 OM28 OM33 OM34 OM35 OM36

OM37 OM38 OM44 OM45 OM46 OM47 OM48 OM55 OM56 OM57 OM58 OM66

OM67 OM68 OM77 OM78 OM88 SG11 SG12 SG13 SG14 SG22 SG23 SG24

SG33 SG34 SG44

OM77

+ 7.89E-03 1.88E-01 1.22E-03 ......... 4.59E-03 4.15E+00 2.15E-01 -1.62E-01 -7.78E-03 -1.04E-02 -6.44E-05 2.02E-05

-1.41E-04 -4.13E-04 1.12E-03 -2.85E-04 2.31E-03 5.68E-04 3.00E-04 ......... ......... ......... ......... .........

......... 8.00E-05 ......... ......... ......... ......... ......... ......... -1.56E-03 ......... ......... .........

......... ......... -1.77E-05 ......... ......... ......... ......... 5.07E-04 ......... ......... ......... 1.54E-03

......... ......... 1.07E-03

OM78

+ ......... ......... ......... ......... ......... ......... ......... ......... ......... ......... ......... .........

......... ......... ......... ......... ......... ......... ......... ......... ......... ......... ......... .........

......... ......... ......... ......... ......... ......... ......... ......... ......... ......... ......... .........

......... ......... ......... ......... ......... ......... ......... ......... ......... ......... ......... .........

......... ......... ......... .........

OM88

+ 3.99E-01 1.11E+01 6.83E-02 ......... 2.45E-01 2.05E+02 2.19E+01 -9.67E+00 -3.61E-01 -4.97E-01 8.66E-03 5.28E-03

2.00E-03 -1.01E-02 3.11E-02 -7.89E-03 3.92E-02 2.05E-02 1.36E-02 ......... ......... ......... ......... .........

......... 1.61E-03 ......... ......... ......... ......... ......... ......... -1.01E-02 ......... ......... .........

......... ......... -5.07E-04 ......... ......... ......... ......... 1.40E-02 ......... ......... ......... 3.23E-02

......... ......... 2.49E-02 ......... 1.85E+00

SG11

+ ......... ......... ......... ......... ......... ......... ......... ......... ......... ......... ......... .........

......... ......... ......... ......... ......... ......... ......... ......... ......... ......... ......... .........

......... ......... ......... ......... ......... ......... ......... ......... ......... ......... ......... .........

......... ......... ......... ......... ......... ......... ......... ......... ......... ......... ......... .........

......... ......... ......... ......... ......... .........

SG12

+ ......... ......... ......... ......... ......... ......... ......... ......... ......... ......... ......... .........

......... ......... ......... ......... ......... ......... ......... ......... ......... ......... ......... .........

......... ......... ......... ......... ......... ......... ......... ......... ......... ......... ......... .........

......... ......... ......... ......... ......... ......... ......... ......... ......... ......... ......... .........

......... ......... ......... ......... ......... ......... .........

SG13

+ ......... ......... ......... ......... ......... ......... ......... ......... ......... ......... ......... .........

......... ......... ......... ......... ......... ......... ......... ......... ......... ......... ......... .........

......... ......... ......... ......... ......... ......... ......... ......... ......... ......... ......... .........

......... ......... ......... ......... ......... ......... ......... ......... ......... ......... ......... .........

......... ......... ......... ......... ......... ......... ......... .........

SG14

+ ......... ......... ......... ......... ......... ......... ......... ......... ......... ......... ......... .........

......... ......... ......... ......... ......... ......... ......... ......... ......... ......... ......... .........

......... ......... ......... ......... ......... ......... ......... ......... ......... ......... ......... .........

......... ......... ......... ......... ......... ......... ......... ......... ......... ......... ......... .........

......... ......... ......... ......... ......... ......... ......... ......... .........

SG22

+ ......... ......... ......... ......... ......... ......... ......... ......... ......... ......... ......... .........

......... ......... ......... ......... ......... ......... ......... ......... ......... ......... ......... .........

......... ......... ......... ......... ......... ......... ......... ......... ......... ......... ......... .........

......... ......... ......... ......... ......... ......... ......... ......... ......... ......... ......... .........

......... ......... ......... ......... ......... ......... ......... ......... ......... .........

1

TH 1 TH 2 TH 3 TH 4 TH 5 TH 6 TH 7 TH 8 TH 9 TH10 TH11 TH12

TH13 TH14 TH15 TH16 TH17 OM11 OM12 OM13 OM14 OM15 OM16 OM17

OM18 OM22 OM23 OM24 OM25 OM26 OM27 OM28 OM33 OM34 OM35 OM36

OM37 OM38 OM44 OM45 OM46 OM47 OM48 OM55 OM56 OM57 OM58 OM66

OM67 OM68 OM77 OM78 OM88 SG11 SG12 SG13 SG14 SG22 SG23 SG24

SG33 SG34 SG44

SG23

+ ......... ......... ......... ......... ......... ......... ......... ......... ......... ......... ......... .........

......... ......... ......... ......... ......... ......... ......... ......... ......... ......... ......... .........

......... ......... ......... ......... ......... ......... ......... ......... ......... ......... ......... .........

......... ......... ......... ......... ......... ......... ......... ......... ......... ......... ......... .........

......... ......... ......... ......... ......... ......... ......... ......... ......... ......... .........

SG24

+ ......... ......... ......... ......... ......... ......... ......... ......... ......... ......... ......... .........

......... ......... ......... ......... ......... ......... ......... ......... ......... ......... ......... .........

......... ......... ......... ......... ......... ......... ......... ......... ......... ......... ......... .........

......... ......... ......... ......... ......... ......... ......... ......... ......... ......... ......... .........

......... ......... ......... ......... ......... ......... ......... ......... ......... ......... ......... .........

SG33

+ ......... ......... ......... ......... ......... ......... ......... ......... ......... ......... ......... .........

......... ......... ......... ......... ......... ......... ......... ......... ......... ......... ......... .........

......... ......... ......... ......... ......... ......... ......... ......... ......... ......... ......... .........

......... ......... ......... ......... ......... ......... ......... ......... ......... ......... ......... .........

......... ......... ......... ......... ......... ......... ......... ......... ......... ......... ......... .........

.........

SG34

+ ......... ......... ......... ......... ......... ......... ......... ......... ......... ......... ......... .........

......... ......... ......... ......... ......... ......... ......... ......... ......... ......... ......... .........

......... ......... ......... ......... ......... ......... ......... ......... ......... ......... ......... .........

......... ......... ......... ......... ......... ......... ......... ......... ......... ......... ......... .........

......... ......... ......... ......... ......... ......... ......... ......... ......... ......... ......... .........

......... .........

SG44

+ ......... ......... ......... ......... ......... ......... ......... ......... ......... ......... ......... .........

......... ......... ......... ......... ......... ......... ......... ......... ......... ......... ......... .........

......... ......... ......... ......... ......... ......... ......... ......... ......... ......... ......... .........

......... ......... ......... ......... ......... ......... ......... ......... ......... ......... ......... .........

......... ......... ......... ......... ......... ......... ......... ......... ......... ......... ......... .........

......... ......... .........

1

************************************************************************************************************************

******************** ********************

******************** FIRST ORDER CONDITIONAL ESTIMATION WITH INTERACTION ********************

******************** CORRELATION MATRIX OF ESTIMATE ********************

******************** ********************

************************************************************************************************************************

TH 1 TH 2 TH 3 TH 4 TH 5 TH 6 TH 7 TH 8 TH 9 TH10 TH11 TH12

TH13 TH14 TH15 TH16 TH17 OM11 OM12 OM13 OM14 OM15 OM16 OM17

OM18 OM22 OM23 OM24 OM25 OM26 OM27 OM28 OM33 OM34 OM35 OM36

OM37 OM38 OM44 OM45 OM46 OM47 OM48 OM55 OM56 OM57 OM58 OM66

OM67 OM68 OM77 OM78 OM88 SG11 SG12 SG13 SG14 SG22 SG23 SG24

SG33 SG34 SG44

TH 1

+ 3.60E-01

TH 2

+ 8.19E-01 1.10E+01

TH 3

+ 4.29E-01 6.26E-01 9.92E-02

TH 4

+ ......... ......... ......... .........

TH 5

+ 5.54E-01 5.52E-01 1.62E-01 ......... 4.89E-01

TH 6

+ 8.91E-01 6.83E-01 4.65E-01 ......... 4.22E-01 1.82E+02

TH 7

+ 5.17E-01 5.00E-01 3.36E-01 ......... 4.79E-01 5.45E-01 3.15E+01

TH 8

+ -4.10E-01 -2.04E-01 3.58E-01 ......... -2.18E-01 -3.03E-01 -5.65E-02 1.77E+01

TH 9

+ -9.75E-01 -7.44E-01 -4.16E-01 ......... -4.89E-01 -8.91E-01 -4.32E-01 4.42E-01 3.30E-01

TH10

+ -8.73E-01 -6.63E-01 -5.01E-01 ......... -3.67E-01 -9.71E-01 -4.10E-01 2.95E-01 9.01E-01 4.33E-01

TH11

+ -8.15E-02 2.19E-01 3.67E-01 ......... -8.98E-02 2.59E-02 1.69E-01 3.98E-01 1.47E-01 -2.36E-02 2.18E-02

TH12

+ 3.47E-01 2.96E-01 -3.19E-01 ......... 3.62E-01 5.02E-02 1.78E-01 -5.90E-01 -2.94E-01 -1.25E-02 -2.34E-01 1.92E-02

TH13

+ -7.99E-02 2.37E-02 -2.39E-01 ......... -8.94E-03 -1.82E-01 1.19E-01 -2.27E-02 1.22E-01 2.04E-01 4.94E-01 3.41E-01

2.27E-02

TH14

+ -3.88E-01 -9.50E-02 1.98E-01 ......... -1.49E-01 -4.81E-01 7.26E-02 5.69E-01 4.35E-01 4.70E-01 5.78E-01 -3.41E-02

4.60E-01 4.19E-02

TH15

+ 5.91E-01 3.19E-01 -3.89E-02 ......... 3.24E-01 3.23E-01 2.39E-01 -3.66E-01 -5.59E-01 -2.40E-01 -4.86E-01 4.97E-01

-1.33E-01 -3.45E-01 1.39E-01

TH16

+ -1.43E-01 -1.41E-01 -3.25E-02 ......... -3.45E-01 -9.50E-02 -7.52E-02 1.69E-02 1.27E-01 8.05E-02 6.23E-02 -1.10E-01

9.61E-03 1.69E-02 -8.59E-02 7.22E-02

TH17

+ 9.96E-02 6.92E-02 -5.24E-02 ......... 8.81E-02 8.92E-02 -6.37E-03 -7.87E-02 -1.29E-01 -1.01E-01 -7.13E-02 5.60E-02

-2.24E-02 -6.96E-02 7.36E-02 -2.44E-02 4.82E-01

OM11

+ 7.82E-01 6.81E-01 1.26E-01 ......... 5.04E-01 6.28E-01 3.22E-01 -5.00E-01 -7.56E-01 -6.21E-01 -1.88E-01 5.70E-01

9.09E-02 -3.50E-01 5.19E-01 -1.43E-01 2.18E-01 2.90E-02

1

TH 1 TH 2 TH 3 TH 4 TH 5 TH 6 TH 7 TH 8 TH 9 TH10 TH11 TH12

TH13 TH14 TH15 TH16 TH17 OM11 OM12 OM13 OM14 OM15 OM16 OM17

OM18 OM22 OM23 OM24 OM25 OM26 OM27 OM28 OM33 OM34 OM35 OM36

OM37 OM38 OM44 OM45 OM46 OM47 OM48 OM55 OM56 OM57 OM58 OM66

OM67 OM68 OM77 OM78 OM88 SG11 SG12 SG13 SG14 SG22 SG23 SG24

SG33 SG34 SG44

OM12

+ 7.96E-01 6.42E-01 7.06E-02 ......... 5.24E-01 6.62E-01 3.90E-01 -5.48E-01 -7.49E-01 -6.43E-01 -1.05E-01 6.35E-01

1.42E-01 -3.25E-01 4.81E-01 -1.44E-01 1.98E-01 9.55E-01 1.63E-02

OM13

+ ......... ......... ......... ......... ......... ......... ......... ......... ......... ......... ......... .........

......... ......... ......... ......... ......... ......... ......... .........

OM14

+ ......... ......... ......... ......... ......... ......... ......... ......... ......... ......... ......... .........

......... ......... ......... ......... ......... ......... ......... ......... .........

OM15

+ ......... ......... ......... ......... ......... ......... ......... ......... ......... ......... ......... .........

......... ......... ......... ......... ......... ......... ......... ......... ......... .........

OM16

+ ......... ......... ......... ......... ......... ......... ......... ......... ......... ......... ......... .........

......... ......... ......... ......... ......... ......... ......... ......... ......... ......... .........

OM17

+ ......... ......... ......... ......... ......... ......... ......... ......... ......... ......... ......... .........

......... ......... ......... ......... ......... ......... ......... ......... ......... ......... ......... .........

OM18

+ ......... ......... ......... ......... ......... ......... ......... ......... ......... ......... ......... .........

......... ......... ......... ......... ......... ......... ......... ......... ......... ......... ......... .........

.........

OM22

+ 3.43E-01 1.67E-01 -1.93E-01 ......... 3.03E-01 2.90E-01 1.99E-02 -5.35E-01 -3.15E-01 -2.59E-01 -4.20E-01 5.05E-01

-2.61E-01 -5.82E-01 4.06E-01 -5.11E-02 1.47E-01 6.06E-01 6.58E-01 ......... ......... ......... ......... .........

......... 1.02E-02

OM23

+ ......... ......... ......... ......... ......... ......... ......... ......... ......... ......... ......... .........

......... ......... ......... ......... ......... ......... ......... ......... ......... ......... ......... .........

......... ......... .........

OM24

+ ......... ......... ......... ......... ......... ......... ......... ......... ......... ......... ......... .........

......... ......... ......... ......... ......... ......... ......... ......... ......... ......... ......... .........

......... ......... ......... .........

OM25

+ ......... ......... ......... ......... ......... ......... ......... ......... ......... ......... ......... .........

......... ......... ......... ......... ......... ......... ......... ......... ......... ......... ......... .........

......... ......... ......... ......... .........

OM26

+ ......... ......... ......... ......... ......... ......... ......... ......... ......... ......... ......... .........

......... ......... ......... ......... ......... ......... ......... ......... ......... ......... ......... .........

......... ......... ......... ......... ......... .........

OM27

+ ......... ......... ......... ......... ......... ......... ......... ......... ......... ......... ......... .........

......... ......... ......... ......... ......... ......... ......... ......... ......... ......... ......... .........

......... ......... ......... ......... ......... ......... .........

1

TH 1 TH 2 TH 3 TH 4 TH 5 TH 6 TH 7 TH 8 TH 9 TH10 TH11 TH12

TH13 TH14 TH15 TH16 TH17 OM11 OM12 OM13 OM14 OM15 OM16 OM17

OM18 OM22 OM23 OM24 OM25 OM26 OM27 OM28 OM33 OM34 OM35 OM36

OM37 OM38 OM44 OM45 OM46 OM47 OM48 OM55 OM56 OM57 OM58 OM66

OM67 OM68 OM77 OM78 OM88 SG11 SG12 SG13 SG14 SG22 SG23 SG24

SG33 SG34 SG44

OM28

+ ......... ......... ......... ......... ......... ......... ......... ......... ......... ......... ......... .........

......... ......... ......... ......... ......... ......... ......... ......... ......... ......... ......... .........

......... ......... ......... ......... ......... ......... ......... .........

OM33

+ -3.20E-01 -1.11E-01 2.06E-01 ......... -2.93E-01 -1.68E-02 -4.25E-02 4.06E-01 3.45E-01 3.59E-02 6.08E-01 -5.08E-01

1.19E-01 6.46E-02 -5.48E-01 1.50E-01 -6.26E-03 -3.47E-01 -3.16E-01 ......... ......... ......... ......... .........

......... -1.02E-01 ......... ......... ......... ......... ......... ......... 2.18E-01

OM34

+ ......... ......... ......... ......... ......... ......... ......... ......... ......... ......... ......... .........

......... ......... ......... ......... ......... ......... ......... ......... ......... ......... ......... .........

......... ......... ......... ......... ......... ......... ......... ......... ......... .........

OM35

+ ......... ......... ......... ......... ......... ......... ......... ......... ......... ......... ......... .........

......... ......... ......... ......... ......... ......... ......... ......... ......... ......... ......... .........

......... ......... ......... ......... ......... ......... ......... ......... ......... ......... .........

OM36

+ ......... ......... ......... ......... ......... ......... ......... ......... ......... ......... ......... .........

......... ......... ......... ......... ......... ......... ......... ......... ......... ......... ......... .........

......... ......... ......... ......... ......... ......... ......... ......... ......... ......... ......... .........

OM37

+ ......... ......... ......... ......... ......... ......... ......... ......... ......... ......... ......... .........

......... ......... ......... ......... ......... ......... ......... ......... ......... ......... ......... .........

......... ......... ......... ......... ......... ......... ......... ......... ......... ......... ......... .........

.........

OM38

+ ......... ......... ......... ......... ......... ......... ......... ......... ......... ......... ......... .........

......... ......... ......... ......... ......... ......... ......... ......... ......... ......... ......... .........

......... ......... ......... ......... ......... ......... ......... ......... ......... ......... ......... .........

......... .........

OM44

+ -1.23E-03 -9.75E-02 -1.22E-01 ......... 8.43E-02 -1.31E-01 -8.30E-02 1.32E-01 -5.33E-03 9.91E-02 -2.51E-01 1.72E-01

4.79E-02 1.09E-01 1.43E-01 -2.24E-01 -2.11E-02 -6.52E-02 -6.21E-02 ......... ......... ......... ......... .........

......... -1.98E-01 ......... ......... ......... ......... ......... ......... -4.26E-01 ......... ......... .........

......... ......... 4.94E-03

OM45

+ ......... ......... ......... ......... ......... ......... ......... ......... ......... ......... ......... .........

......... ......... ......... ......... ......... ......... ......... ......... ......... ......... ......... .........

......... ......... ......... ......... ......... ......... ......... ......... ......... ......... ......... .........

......... ......... ......... .........

OM46

+ ......... ......... ......... ......... ......... ......... ......... ......... ......... ......... ......... .........

......... ......... ......... ......... ......... ......... ......... ......... ......... ......... ......... .........

......... ......... ......... ......... ......... ......... ......... ......... ......... ......... ......... .........

......... ......... ......... ......... .........

1

TH 1 TH 2 TH 3 TH 4 TH 5 TH 6 TH 7 TH 8 TH 9 TH10 TH11 TH12

TH13 TH14 TH15 TH16 TH17 OM11 OM12 OM13 OM14 OM15 OM16 OM17

OM18 OM22 OM23 OM24 OM25 OM26 OM27 OM28 OM33 OM34 OM35 OM36

OM37 OM38 OM44 OM45 OM46 OM47 OM48 OM55 OM56 OM57 OM58 OM66

OM67 OM68 OM77 OM78 OM88 SG11 SG12 SG13 SG14 SG22 SG23 SG24

SG33 SG34 SG44

OM47

+ ......... ......... ......... ......... ......... ......... ......... ......... ......... ......... ......... .........

......... ......... ......... ......... ......... ......... ......... ......... ......... ......... ......... .........

......... ......... ......... ......... ......... ......... ......... ......... ......... ......... ......... .........

......... ......... ......... ......... ......... .........

OM48

+ ......... ......... ......... ......... ......... ......... ......... ......... ......... ......... ......... .........

......... ......... ......... ......... ......... ......... ......... ......... ......... ......... ......... .........

......... ......... ......... ......... ......... ......... ......... ......... ......... ......... ......... .........

......... ......... ......... ......... ......... ......... .........

OM55

+ 5.51E-01 4.68E-01 3.93E-01 ......... 1.79E-01 6.10E-01 1.88E-01 -1.82E-01 -5.81E-01 -6.36E-01 -6.80E-02 -6.47E-02

-2.75E-01 -3.98E-01 1.79E-01 2.02E-02 1.67E-01 4.74E-01 4.10E-01 ......... ......... ......... ......... .........

......... 2.30E-01 ......... ......... ......... ......... ......... ......... 1.42E-03 ......... ......... .........

......... ......... -2.37E-01 ......... ......... ......... ......... 2.43E-02

OM56

+ ......... ......... ......... ......... ......... ......... ......... ......... ......... ......... ......... .........

......... ......... ......... ......... ......... ......... ......... ......... ......... ......... ......... .........

......... ......... ......... ......... ......... ......... ......... ......... ......... ......... ......... .........

......... ......... ......... ......... ......... ......... ......... ......... .........

OM57

+ ......... ......... ......... ......... ......... ......... ......... ......... ......... ......... ......... .........

......... ......... ......... ......... ......... ......... ......... ......... ......... ......... ......... .........

......... ......... ......... ......... ......... ......... ......... ......... ......... ......... ......... .........

......... ......... ......... ......... ......... ......... ......... ......... ......... .........

OM58

+ ......... ......... ......... ......... ......... ......... ......... ......... ......... ......... ......... .........

......... ......... ......... ......... ......... ......... ......... ......... ......... ......... ......... .........

......... ......... ......... ......... ......... ......... ......... ......... ......... ......... ......... .........

......... ......... ......... ......... ......... ......... ......... ......... ......... ......... .........

OM66

+ 5.01E-01 3.85E-01 3.84E-01 ......... 2.03E-01 6.07E-01 2.11E-01 -6.90E-02 -5.37E-01 -5.93E-01 -3.59E-02 -7.54E-02

-3.46E-01 -1.57E-01 2.13E-01 -3.69E-02 1.48E-01 4.32E-01 4.04E-01 ......... ......... ......... ......... .........

......... 2.16E-01 ......... ......... ......... ......... ......... ......... -1.43E-01 ......... ......... .........

......... ......... -6.72E-02 ......... ......... ......... ......... 5.40E-01 ......... ......... ......... 5.90E-02

OM67

+ ......... ......... ......... ......... ......... ......... ......... ......... ......... ......... ......... .........

......... ......... ......... ......... ......... ......... ......... ......... ......... ......... ......... .........

......... ......... ......... ......... ......... ......... ......... ......... ......... ......... ......... .........

......... ......... ......... ......... ......... ......... ......... ......... ......... ......... ......... .........

.........

OM68

+ ......... ......... ......... ......... ......... ......... ......... ......... ......... ......... ......... .........

......... ......... ......... ......... ......... ......... ......... ......... ......... ......... ......... .........

......... ......... ......... ......... ......... ......... ......... ......... ......... ......... ......... .........

......... ......... ......... ......... ......... ......... ......... ......... ......... ......... ......... .........

......... .........

1

TH 1 TH 2 TH 3 TH 4 TH 5 TH 6 TH 7 TH 8 TH 9 TH10 TH11 TH12

TH13 TH14 TH15 TH16 TH17 OM11 OM12 OM13 OM14 OM15 OM16 OM17

OM18 OM22 OM23 OM24 OM25 OM26 OM27 OM28 OM33 OM34 OM35 OM36

OM37 OM38 OM44 OM45 OM46 OM47 OM48 OM55 OM56 OM57 OM58 OM66

OM67 OM68 OM77 OM78 OM88 SG11 SG12 SG13 SG14 SG22 SG23 SG24

SG33 SG34 SG44

OM77

+ 6.69E-01 5.20E-01 3.75E-01 ......... 2.87E-01 6.99E-01 2.08E-01 -2.80E-01 -7.19E-01 -7.31E-01 -9.04E-02 3.20E-02

-1.90E-01 -3.01E-01 2.47E-01 -1.21E-01 1.46E-01 5.97E-01 5.62E-01 ......... ......... ......... ......... .........

......... 2.40E-01 ......... ......... ......... ......... ......... ......... -2.19E-01 ......... ......... .........

......... ......... -1.09E-01 ......... ......... ......... ......... 6.37E-01 ......... ......... ......... 7.99E-01

......... ......... 3.27E-02

OM78

+ ......... ......... ......... ......... ......... ......... ......... ......... ......... ......... ......... .........

......... ......... ......... ......... ......... ......... ......... ......... ......... ......... ......... .........

......... ......... ......... ......... ......... ......... ......... ......... ......... ......... ......... .........

......... ......... ......... ......... ......... ......... ......... ......... ......... ......... ......... .........

......... ......... ......... .........

OM88

+ 8.13E-01 7.37E-01 5.06E-01 ......... 3.68E-01 8.29E-01 5.10E-01 -4.03E-01 -8.03E-01 -8.43E-01 2.92E-01 2.02E-01

6.45E-02 -1.77E-01 1.64E-01 -8.04E-02 5.98E-02 5.18E-01 6.11E-01 ......... ......... ......... ......... .........

......... 1.17E-01 ......... ......... ......... ......... ......... ......... -3.42E-02 ......... ......... .........

......... ......... -7.53E-02 ......... ......... ......... ......... 4.23E-01 ......... ......... ......... 4.02E-01

......... ......... 5.58E-01 ......... 1.36E+00

SG11

+ ......... ......... ......... ......... ......... ......... ......... ......... ......... ......... ......... .........

......... ......... ......... ......... ......... ......... ......... ......... ......... ......... ......... .........

......... ......... ......... ......... ......... ......... ......... ......... ......... ......... ......... .........

......... ......... ......... ......... ......... ......... ......... ......... ......... ......... ......... .........

......... ......... ......... ......... ......... .........

SG12

+ ......... ......... ......... ......... ......... ......... ......... ......... ......... ......... ......... .........

......... ......... ......... ......... ......... ......... ......... ......... ......... ......... ......... .........

......... ......... ......... ......... ......... ......... ......... ......... ......... ......... ......... .........

......... ......... ......... ......... ......... ......... ......... ......... ......... ......... ......... .........

......... ......... ......... ......... ......... ......... .........

SG13

+ ......... ......... ......... ......... ......... ......... ......... ......... ......... ......... ......... .........

......... ......... ......... ......... ......... ......... ......... ......... ......... ......... ......... .........

......... ......... ......... ......... ......... ......... ......... ......... ......... ......... ......... .........

......... ......... ......... ......... ......... ......... ......... ......... ......... ......... ......... .........

......... ......... ......... ......... ......... ......... ......... .........

SG14

+ ......... ......... ......... ......... ......... ......... ......... ......... ......... ......... ......... .........

......... ......... ......... ......... ......... ......... ......... ......... ......... ......... ......... .........

......... ......... ......... ......... ......... ......... ......... ......... ......... ......... ......... .........

......... ......... ......... ......... ......... ......... ......... ......... ......... ......... ......... .........

......... ......... ......... ......... ......... ......... ......... ......... .........

SG22

+ ......... ......... ......... ......... ......... ......... ......... ......... ......... ......... ......... .........

......... ......... ......... ......... ......... ......... ......... ......... ......... ......... ......... .........

......... ......... ......... ......... ......... ......... ......... ......... ......... ......... ......... .........

......... ......... ......... ......... ......... ......... ......... ......... ......... ......... ......... .........

......... ......... ......... ......... ......... ......... ......... ......... ......... .........

1

TH 1 TH 2 TH 3 TH 4 TH 5 TH 6 TH 7 TH 8 TH 9 TH10 TH11 TH12

TH13 TH14 TH15 TH16 TH17 OM11 OM12 OM13 OM14 OM15 OM16 OM17

OM18 OM22 OM23 OM24 OM25 OM26 OM27 OM28 OM33 OM34 OM35 OM36

OM37 OM38 OM44 OM45 OM46 OM47 OM48 OM55 OM56 OM57 OM58 OM66

OM67 OM68 OM77 OM78 OM88 SG11 SG12 SG13 SG14 SG22 SG23 SG24

SG33 SG34 SG44

SG23

+ ......... ......... ......... ......... ......... ......... ......... ......... ......... ......... ......... .........

......... ......... ......... ......... ......... ......... ......... ......... ......... ......... ......... .........

......... ......... ......... ......... ......... ......... ......... ......... ......... ......... ......... .........

......... ......... ......... ......... ......... ......... ......... ......... ......... ......... ......... .........

......... ......... ......... ......... ......... ......... ......... ......... ......... ......... .........

SG24

+ ......... ......... ......... ......... ......... ......... ......... ......... ......... ......... ......... .........

......... ......... ......... ......... ......... ......... ......... ......... ......... ......... ......... .........

......... ......... ......... ......... ......... ......... ......... ......... ......... ......... ......... .........

......... ......... ......... ......... ......... ......... ......... ......... ......... ......... ......... .........

......... ......... ......... ......... ......... ......... ......... ......... ......... ......... ......... .........

SG33

+ ......... ......... ......... ......... ......... ......... ......... ......... ......... ......... ......... .........

......... ......... ......... ......... ......... ......... ......... ......... ......... ......... ......... .........

......... ......... ......... ......... ......... ......... ......... ......... ......... ......... ......... .........

......... ......... ......... ......... ......... ......... ......... ......... ......... ......... ......... .........

......... ......... ......... ......... ......... ......... ......... ......... ......... ......... ......... .........

.........

SG34

+ ......... ......... ......... ......... ......... ......... ......... ......... ......... ......... ......... .........

......... ......... ......... ......... ......... ......... ......... ......... ......... ......... ......... .........

......... ......... ......... ......... ......... ......... ......... ......... ......... ......... ......... .........

......... ......... ......... ......... ......... ......... ......... ......... ......... ......... ......... .........

......... ......... ......... ......... ......... ......... ......... ......... ......... ......... ......... .........

......... .........

SG44

+ ......... ......... ......... ......... ......... ......... ......... ......... ......... ......... ......... .........

......... ......... ......... ......... ......... ......... ......... ......... ......... ......... ......... .........

......... ......... ......... ......... ......... ......... ......... ......... ......... ......... ......... .........

......... ......... ......... ......... ......... ......... ......... ......... ......... ......... ......... .........

......... ......... ......... ......... ......... ......... ......... ......... ......... ......... ......... .........

......... ......... .........

1

************************************************************************************************************************

******************** ********************

******************** FIRST ORDER CONDITIONAL ESTIMATION WITH INTERACTION ********************

******************** INVERSE COVARIANCE MATRIX OF ESTIMATE ********************

******************** ********************

************************************************************************************************************************

TH 1 TH 2 TH 3 TH 4 TH 5 TH 6 TH 7 TH 8 TH 9 TH10 TH11 TH12

TH13 TH14 TH15 TH16 TH17 OM11 OM12 OM13 OM14 OM15 OM16 OM17

OM18 OM22 OM23 OM24 OM25 OM26 OM27 OM28 OM33 OM34 OM35 OM36

OM37 OM38 OM44 OM45 OM46 OM47 OM48 OM55 OM56 OM57 OM58 OM66

OM67 OM68 OM77 OM78 OM88 SG11 SG12 SG13 SG14 SG22 SG23 SG24

SG33 SG34 SG44

TH 1

+ 1.25E+04

TH 2

+ -1.57E+02 2.13E+00

TH 3

+ 1.06E+03 -2.31E+01 2.02E+03

TH 4

+ ......... ......... ......... .........

TH 5

+ -1.17E+02 9.90E-01 2.66E+01 ......... 1.21E+01

TH 6

+ -8.56E-01 7.66E-03 -4.10E-01 ......... 2.78E-04 4.13E-03

TH 7

+ -3.94E-01 6.81E-03 -7.91E-03 ......... -5.77E-02 -3.46E-03 5.77E-03

TH 8

+ 2.88E+01 -3.97E-01 -1.75E+00 ......... -3.45E-01 -1.72E-03 -3.00E-04 1.71E-01

TH 9

+ 3.93E+03 -4.67E+01 2.06E+02 ......... -2.80E+01 -1.12E-01 -3.20E-01 9.77E+00 1.94E+03

TH10

+ 4.94E+03 -6.83E+01 6.01E+02 ......... -4.44E+01 7.21E-01 -1.12E+00 1.46E+01 1.13E+03 2.83E+03

TH11

+ 6.13E+03 -9.55E+01 1.57E+03 ......... -9.58E+01 1.00E+00 2.41E+00 8.99E+00 2.25E+03 2.11E+03 3.17E+04

TH12

+ 2.81E+04 -3.96E+02 5.94E+03 ......... -1.54E+02 -2.66E+00 -1.00E-01 8.27E+01 8.94E+03 1.19E+04 2.06E+04 9.69E+04

TH13

+ -7.08E+03 1.11E+02 -2.45E+03 ......... -1.48E+01 7.24E-01 5.30E-01 1.96E+00 -2.40E+02 -4.09E+03 -6.49E+03 -2.31E+04

2.73E+04

TH14

+ -1.35E+04 2.02E+02 -4.48E+03 ......... 7.98E+01 4.85E+00 -3.42E+00 -5.62E+01 -3.71E+03 -6.36E+03 -1.01E+04 -4.65E+04

7.89E+03 3.70E+04

TH15

+ -8.20E+03 1.06E+02 -7.69E+02 ......... 8.78E+01 1.92E-01 3.84E-01 -2.07E+01 -2.17E+03 -3.70E+03 -3.98E+03 -1.91E+04

5.32E+03 9.62E+03 5.82E+03

TH16

+ -4.87E+01 -6.69E-02 2.03E+01 ......... 1.52E+01 2.07E-01 -2.23E-01 -2.14E-01 -2.39E+01 6.17E+01 7.36E+01 -5.07E+01

-2.85E+02 1.85E+02 6.73E+00 2.52E+02

TH17

+ 9.40E+01 -1.12E+00 1.71E+01 ......... -1.53E+00 1.42E-02 -2.62E-03 2.26E-01 4.91E+01 2.64E+01 1.48E+02 2.55E+02

3.73E+01 -1.09E+02 -5.50E+01 -6.80E-01 6.69E+00

OM11

+ 7.59E+04 -9.87E+02 2.56E+02 ......... -7.32E+02 3.95E+00 -5.44E+00 2.34E+02 2.67E+04 3.20E+04 5.39E+04 1.77E+05

-2.84E+04 -7.95E+04 -5.02E+04 4.44E+01 6.51E+02 5.74E+05

1

TH 1 TH 2 TH 3 TH 4 TH 5 TH 6 TH 7 TH 8 TH 9 TH10 TH11 TH12

TH13 TH14 TH15 TH16 TH17 OM11 OM12 OM13 OM14 OM15 OM16 OM17

OM18 OM22 OM23 OM24 OM25 OM26 OM27 OM28 OM33 OM34 OM35 OM36

OM37 OM38 OM44 OM45 OM46 OM47 OM48 OM55 OM56 OM57 OM58 OM66

OM67 OM68 OM77 OM78 OM88 SG11 SG12 SG13 SG14 SG22 SG23 SG24

SG33 SG34 SG44

OM12

+ -1.50E+05 1.84E+03 1.28E+04 ......... 1.86E+03 -1.52E+01 1.11E+01 -4.84E+02 -5.81E+04 -5.79E+04 -1.18E+05 -3.23E+05

5.29E+03 1.23E+05 9.69E+04 4.75E+02 -1.62E+03 -1.19E+06 2.71E+06

OM13

+ ......... ......... ......... ......... ......... ......... ......... ......... ......... ......... ......... .........

......... ......... ......... ......... ......... ......... ......... .........

OM14

+ ......... ......... ......... ......... ......... ......... ......... ......... ......... ......... ......... .........

......... ......... ......... ......... ......... ......... ......... ......... .........

OM15

+ ......... ......... ......... ......... ......... ......... ......... ......... ......... ......... ......... .........

......... ......... ......... ......... ......... ......... ......... ......... ......... .........

OM16

+ ......... ......... ......... ......... ......... ......... ......... ......... ......... ......... ......... .........

......... ......... ......... ......... ......... ......... ......... ......... ......... ......... .........

OM17

+ ......... ......... ......... ......... ......... ......... ......... ......... ......... ......... ......... .........

......... ......... ......... ......... ......... ......... ......... ......... ......... ......... ......... .........

OM18

+ ......... ......... ......... ......... ......... ......... ......... ......... ......... ......... ......... .........

......... ......... ......... ......... ......... ......... ......... ......... ......... ......... ......... .........

.........

OM22

+ 2.17E+03 9.40E+01 -2.14E+04 ......... -7.31E+02 1.69E+01 -3.72E+00 7.29E+01 7.78E+03 -2.35E+03 2.35E+04 -4.99E+04

7.28E+04 4.68E+04 1.21E+01 -3.02E+02 3.75E+02 1.63E+05 -6.36E+05 ......... ......... ......... ......... .........

......... 5.28E+05

OM23

+ ......... ......... ......... ......... ......... ......... ......... ......... ......... ......... ......... .........

......... ......... ......... ......... ......... ......... ......... ......... ......... ......... ......... .........

......... ......... .........

OM24

+ ......... ......... ......... ......... ......... ......... ......... ......... ......... ......... ......... .........

......... ......... ......... ......... ......... ......... ......... ......... ......... ......... ......... .........

......... ......... ......... .........

OM25

+ ......... ......... ......... ......... ......... ......... ......... ......... ......... ......... ......... .........

......... ......... ......... ......... ......... ......... ......... ......... ......... ......... ......... .........

......... ......... ......... ......... .........

OM26

+ ......... ......... ......... ......... ......... ......... ......... ......... ......... ......... ......... .........

......... ......... ......... ......... ......... ......... ......... ......... ......... ......... ......... .........

......... ......... ......... ......... ......... .........

OM27

+ ......... ......... ......... ......... ......... ......... ......... ......... ......... ......... ......... .........

......... ......... ......... ......... ......... ......... ......... ......... ......... ......... ......... .........

......... ......... ......... ......... ......... ......... .........

1

TH 1 TH 2 TH 3 TH 4 TH 5 TH 6 TH 7 TH 8 TH 9 TH10 TH11 TH12

TH13 TH14 TH15 TH16 TH17 OM11 OM12 OM13 OM14 OM15 OM16 OM17

OM18 OM22 OM23 OM24 OM25 OM26 OM27 OM28 OM33 OM34 OM35 OM36

OM37 OM38 OM44 OM45 OM46 OM47 OM48 OM55 OM56 OM57 OM58 OM66

OM67 OM68 OM77 OM78 OM88 SG11 SG12 SG13 SG14 SG22 SG23 SG24

SG33 SG34 SG44

OM28

+ ......... ......... ......... ......... ......... ......... ......... ......... ......... ......... ......... .........

......... ......... ......... ......... ......... ......... ......... ......... ......... ......... ......... .........

......... ......... ......... ......... ......... ......... ......... .........

OM33

+ 5.36E+02 -7.44E+00 3.64E+02 ......... 1.16E+01 -5.52E-01 8.70E-02 -2.57E+00 -1.71E+02 2.56E+02 -1.34E+03 1.40E+03

-2.74E+03 -4.59E+02 -3.79E+02 7.06E+00 -1.60E+01 -1.80E+03 1.11E+04 ......... ......... ......... ......... .........

......... -1.29E+04 ......... ......... ......... ......... ......... ......... 5.73E+02

OM34

+ ......... ......... ......... ......... ......... ......... ......... ......... ......... ......... ......... .........

......... ......... ......... ......... ......... ......... ......... ......... ......... ......... ......... .........

......... ......... ......... ......... ......... ......... ......... ......... ......... .........

OM35

+ ......... ......... ......... ......... ......... ......... ......... ......... ......... ......... ......... .........

......... ......... ......... ......... ......... ......... ......... ......... ......... ......... ......... .........

......... ......... ......... ......... ......... ......... ......... ......... ......... ......... .........

OM36

+ ......... ......... ......... ......... ......... ......... ......... ......... ......... ......... ......... .........

......... ......... ......... ......... ......... ......... ......... ......... ......... ......... ......... .........

......... ......... ......... ......... ......... ......... ......... ......... ......... ......... ......... .........

OM37

+ ......... ......... ......... ......... ......... ......... ......... ......... ......... ......... ......... .........

......... ......... ......... ......... ......... ......... ......... ......... ......... ......... ......... .........

......... ......... ......... ......... ......... ......... ......... ......... ......... ......... ......... .........

.........

OM38

+ ......... ......... ......... ......... ......... ......... ......... ......... ......... ......... ......... .........

......... ......... ......... ......... ......... ......... ......... ......... ......... ......... ......... .........

......... ......... ......... ......... ......... ......... ......... ......... ......... ......... ......... .........

......... .........

OM44

+ -3.00E+04 4.11E+02 -2.02E+03 ......... 1.98E+02 6.62E+00 2.65E-01 -1.57E+02 -1.08E+04 -1.29E+04 4.13E+02 -9.38E+04

-7.29E+02 6.22E+04 2.03E+04 1.22E+03 -2.57E+02 -2.08E+05 4.08E+05 ......... ......... ......... ......... .........

......... 9.96E+02 ......... ......... ......... ......... ......... ......... 1.46E+03 ......... ......... .........

......... ......... 2.28E+05

OM45

+ ......... ......... ......... ......... ......... ......... ......... ......... ......... ......... ......... .........

......... ......... ......... ......... ......... ......... ......... ......... ......... ......... ......... .........

......... ......... ......... ......... ......... ......... ......... ......... ......... ......... ......... .........

......... ......... ......... .........

OM46

+ ......... ......... ......... ......... ......... ......... ......... ......... ......... ......... ......... .........

......... ......... ......... ......... ......... ......... ......... ......... ......... ......... ......... .........

......... ......... ......... ......... ......... ......... ......... ......... ......... ......... ......... .........

......... ......... ......... ......... .........

1

TH 1 TH 2 TH 3 TH 4 TH 5 TH 6 TH 7 TH 8 TH 9 TH10 TH11 TH12

TH13 TH14 TH15 TH16 TH17 OM11 OM12 OM13 OM14 OM15 OM16 OM17

OM18 OM22 OM23 OM24 OM25 OM26 OM27 OM28 OM33 OM34 OM35 OM36

OM37 OM38 OM44 OM45 OM46 OM47 OM48 OM55 OM56 OM57 OM58 OM66

OM67 OM68 OM77 OM78 OM88 SG11 SG12 SG13 SG14 SG22 SG23 SG24

SG33 SG34 SG44

OM47

+ ......... ......... ......... ......... ......... ......... ......... ......... ......... ......... ......... .........

......... ......... ......... ......... ......... ......... ......... ......... ......... ......... ......... .........

......... ......... ......... ......... ......... ......... ......... ......... ......... ......... ......... .........

......... ......... ......... ......... ......... .........

OM48

+ ......... ......... ......... ......... ......... ......... ......... ......... ......... ......... ......... .........

......... ......... ......... ......... ......... ......... ......... ......... ......... ......... ......... .........

......... ......... ......... ......... ......... ......... ......... ......... ......... ......... ......... .........

......... ......... ......... ......... ......... ......... .........

OM55

+ -3.87E+03 5.25E+01 -1.38E+03 ......... 3.42E+01 1.27E+00 -8.39E-01 -6.62E+00 -9.31E+02 -1.43E+03 -2.81E+03 -1.17E+04

4.66E+03 7.27E+03 2.64E+03 -2.81E+01 -3.91E+01 -1.92E+04 2.73E+04 ......... ......... ......... ......... .........

......... 1.74E+04 ......... ......... ......... ......... ......... ......... -5.50E+02 ......... ......... .........

......... ......... 1.16E+04 ......... ......... ......... ......... 6.01E+03

OM56

+ ......... ......... ......... ......... ......... ......... ......... ......... ......... ......... ......... .........

......... ......... ......... ......... ......... ......... ......... ......... ......... ......... ......... .........

......... ......... ......... ......... ......... ......... ......... ......... ......... ......... ......... .........

......... ......... ......... ......... ......... ......... ......... ......... .........

OM57

+ ......... ......... ......... ......... ......... ......... ......... ......... ......... ......... ......... .........

......... ......... ......... ......... ......... ......... ......... ......... ......... ......... ......... .........

......... ......... ......... ......... ......... ......... ......... ......... ......... ......... ......... .........

......... ......... ......... ......... ......... ......... ......... ......... ......... .........

OM58

+ ......... ......... ......... ......... ......... ......... ......... ......... ......... ......... ......... .........

......... ......... ......... ......... ......... ......... ......... ......... ......... ......... ......... .........

......... ......... ......... ......... ......... ......... ......... ......... ......... ......... ......... .........

......... ......... ......... ......... ......... ......... ......... ......... ......... ......... .........

OM66

+ 4.83E+03 -6.27E+01 1.14E+03 ......... -3.11E+01 -2.64E+00 1.41E+00 1.36E+01 1.15E+03 1.77E+03 -1.46E+02 1.29E+04

-2.30E+03 -1.01E+04 -3.27E+03 -1.85E+02 1.52E+01 2.23E+04 -3.40E+04 ......... ......... ......... ......... .........

......... -1.80E+04 ......... ......... ......... ......... ......... ......... 6.11E+02 ......... ......... .........

......... ......... -1.89E+04 ......... ......... ......... ......... -2.44E+03 ......... ......... ......... 4.90E+03

OM67

+ ......... ......... ......... ......... ......... ......... ......... ......... ......... ......... ......... .........

......... ......... ......... ......... ......... ......... ......... ......... ......... ......... ......... .........

......... ......... ......... ......... ......... ......... ......... ......... ......... ......... ......... .........

......... ......... ......... ......... ......... ......... ......... ......... ......... ......... ......... .........

.........

OM68

+ ......... ......... ......... ......... ......... ......... ......... ......... ......... ......... ......... .........

......... ......... ......... ......... ......... ......... ......... ......... ......... ......... ......... .........

......... ......... ......... ......... ......... ......... ......... ......... ......... ......... ......... .........

......... ......... ......... ......... ......... ......... ......... ......... ......... ......... ......... .........

......... .........

1

TH 1 TH 2 TH 3 TH 4 TH 5 TH 6 TH 7 TH 8 TH 9 TH10 TH11 TH12

TH13 TH14 TH15 TH16 TH17 OM11 OM12 OM13 OM14 OM15 OM16 OM17

OM18 OM22 OM23 OM24 OM25 OM26 OM27 OM28 OM33 OM34 OM35 OM36

OM37 OM38 OM44 OM45 OM46 OM47 OM48 OM55 OM56 OM57 OM58 OM66

OM67 OM68 OM77 OM78 OM88 SG11 SG12 SG13 SG14 SG22 SG23 SG24

SG33 SG34 SG44

OM77

+ 1.07E+03 -1.59E+01 -4.31E+02 ......... -1.08E+01 1.45E+00 -4.92E-01 5.66E+00 7.62E+02 7.02E+02 3.52E+03 4.33E+03

-1.53E+03 1.99E+02 -5.79E+02 2.52E+02 2.73E+01 1.40E+04 -3.49E+04 ......... ......... ......... ......... .........

......... 1.27E+04 ......... ......... ......... ......... ......... ......... -2.91E+02 ......... ......... .........

......... ......... 8.30E+02 ......... ......... ......... ......... -6.42E+02 ......... ......... ......... -2.36E+03

......... ......... 6.41E+03

OM78

+ ......... ......... ......... ......... ......... ......... ......... ......... ......... ......... ......... .........

......... ......... ......... ......... ......... ......... ......... ......... ......... ......... ......... .........

......... ......... ......... ......... ......... ......... ......... ......... ......... ......... ......... .........

......... ......... ......... ......... ......... ......... ......... ......... ......... ......... ......... .........

......... ......... ......... .........

OM88

+ 7.04E+02 -9.54E+00 -4.40E+01 ......... -6.18E+00 -4.83E-03 -6.40E-02 3.52E+00 2.54E+02 3.47E+02 1.48E+02 1.74E+03

-2.95E+01 -1.10E+03 -4.82E+02 -2.55E+00 4.90E+00 5.74E+03 -1.18E+04 ......... ......... ......... ......... .........

......... 1.84E+03 ......... ......... ......... ......... ......... ......... -4.38E+01 ......... ......... .........

......... ......... -3.18E+03 ......... ......... ......... ......... -1.24E+02 ......... ......... ......... 2.91E+02

......... ......... 1.27E+02 ......... 8.20E+01

SG11

+ ......... ......... ......... ......... ......... ......... ......... ......... ......... ......... ......... .........

......... ......... ......... ......... ......... ......... ......... ......... ......... ......... ......... .........

......... ......... ......... ......... ......... ......... ......... ......... ......... ......... ......... .........

......... ......... ......... ......... ......... ......... ......... ......... ......... ......... ......... .........

......... ......... ......... ......... ......... .........

SG12

+ ......... ......... ......... ......... ......... ......... ......... ......... ......... ......... ......... .........

......... ......... ......... ......... ......... ......... ......... ......... ......... ......... ......... .........

......... ......... ......... ......... ......... ......... ......... ......... ......... ......... ......... .........

......... ......... ......... ......... ......... ......... ......... ......... ......... ......... ......... .........

......... ......... ......... ......... ......... ......... .........

SG13

+ ......... ......... ......... ......... ......... ......... ......... ......... ......... ......... ......... .........

......... ......... ......... ......... ......... ......... ......... ......... ......... ......... ......... .........

......... ......... ......... ......... ......... ......... ......... ......... ......... ......... ......... .........

......... ......... ......... ......... ......... ......... ......... ......... ......... ......... ......... .........

......... ......... ......... ......... ......... ......... ......... .........

SG14

+ ......... ......... ......... ......... ......... ......... ......... ......... ......... ......... ......... .........

......... ......... ......... ......... ......... ......... ......... ......... ......... ......... ......... .........

......... ......... ......... ......... ......... ......... ......... ......... ......... ......... ......... .........

......... ......... ......... ......... ......... ......... ......... ......... ......... ......... ......... .........

......... ......... ......... ......... ......... ......... ......... ......... .........

SG22

+ ......... ......... ......... ......... ......... ......... ......... ......... ......... ......... ......... .........

......... ......... ......... ......... ......... ......... ......... ......... ......... ......... ......... .........

......... ......... ......... ......... ......... ......... ......... ......... ......... ......... ......... .........

......... ......... ......... ......... ......... ......... ......... ......... ......... ......... ......... .........

......... ......... ......... ......... ......... ......... ......... ......... ......... .........

1

TH 1 TH 2 TH 3 TH 4 TH 5 TH 6 TH 7 TH 8 TH 9 TH10 TH11 TH12

TH13 TH14 TH15 TH16 TH17 OM11 OM12 OM13 OM14 OM15 OM16 OM17

OM18 OM22 OM23 OM24 OM25 OM26 OM27 OM28 OM33 OM34 OM35 OM36

OM37 OM38 OM44 OM45 OM46 OM47 OM48 OM55 OM56 OM57 OM58 OM66

OM67 OM68 OM77 OM78 OM88 SG11 SG12 SG13 SG14 SG22 SG23 SG24

SG33 SG34 SG44

SG23

+ ......... ......... ......... ......... ......... ......... ......... ......... ......... ......... ......... .........

......... ......... ......... ......... ......... ......... ......... ......... ......... ......... ......... .........

......... ......... ......... ......... ......... ......... ......... ......... ......... ......... ......... .........

......... ......... ......... ......... ......... ......... ......... ......... ......... ......... ......... .........

......... ......... ......... ......... ......... ......... ......... ......... ......... ......... .........

SG24

+ ......... ......... ......... ......... ......... ......... ......... ......... ......... ......... ......... .........

......... ......... ......... ......... ......... ......... ......... ......... ......... ......... ......... .........

......... ......... ......... ......... ......... ......... ......... ......... ......... ......... ......... .........

......... ......... ......... ......... ......... ......... ......... ......... ......... ......... ......... .........

......... ......... ......... ......... ......... ......... ......... ......... ......... ......... ......... .........

SG33

+ ......... ......... ......... ......... ......... ......... ......... ......... ......... ......... ......... .........

......... ......... ......... ......... ......... ......... ......... ......... ......... ......... ......... .........

......... ......... ......... ......... ......... ......... ......... ......... ......... ......... ......... .........

......... ......... ......... ......... ......... ......... ......... ......... ......... ......... ......... .........

......... ......... ......... ......... ......... ......... ......... ......... ......... ......... ......... .........

.........

SG34

+ ......... ......... ......... ......... ......... ......... ......... ......... ......... ......... ......... .........

......... ......... ......... ......... ......... ......... ......... ......... ......... ......... ......... .........

......... ......... ......... ......... ......... ......... ......... ......... ......... ......... ......... .........

......... ......... ......... ......... ......... ......... ......... ......... ......... ......... ......... .........

......... ......... ......... ......... ......... ......... ......... ......... ......... ......... ......... .........

......... .........

SG44

+ ......... ......... ......... ......... ......... ......... ......... ......... ......... ......... ......... .........

......... ......... ......... ......... ......... ......... ......... ......... ......... ......... ......... .........

......... ......... ......... ......... ......... ......... ......... ......... ......... ......... ......... .........

......... ......... ......... ......... ......... ......... ......... ......... ......... ......... ......... .........

......... ......... ......... ......... ......... ......... ......... ......... ......... ......... ......... .........

......... ......... .........

#CPUT: Total CPU Time in Seconds, 4845.142

Stop Time:

13/10/2020

07:54
